# Supplementary figures and images for: Trajectory-centric framework TrajAtlas reveals multi-scale differentiation heterogeneity among cells, genes, and gene modules in osteogenesis
Source: PLoS Genet. 2024 Oct 22;20(10):e1011319. doi: 10.1371/journal.pgen.1011319 (PMC11530032; doi:10.1371/journal.pgen.1011319)

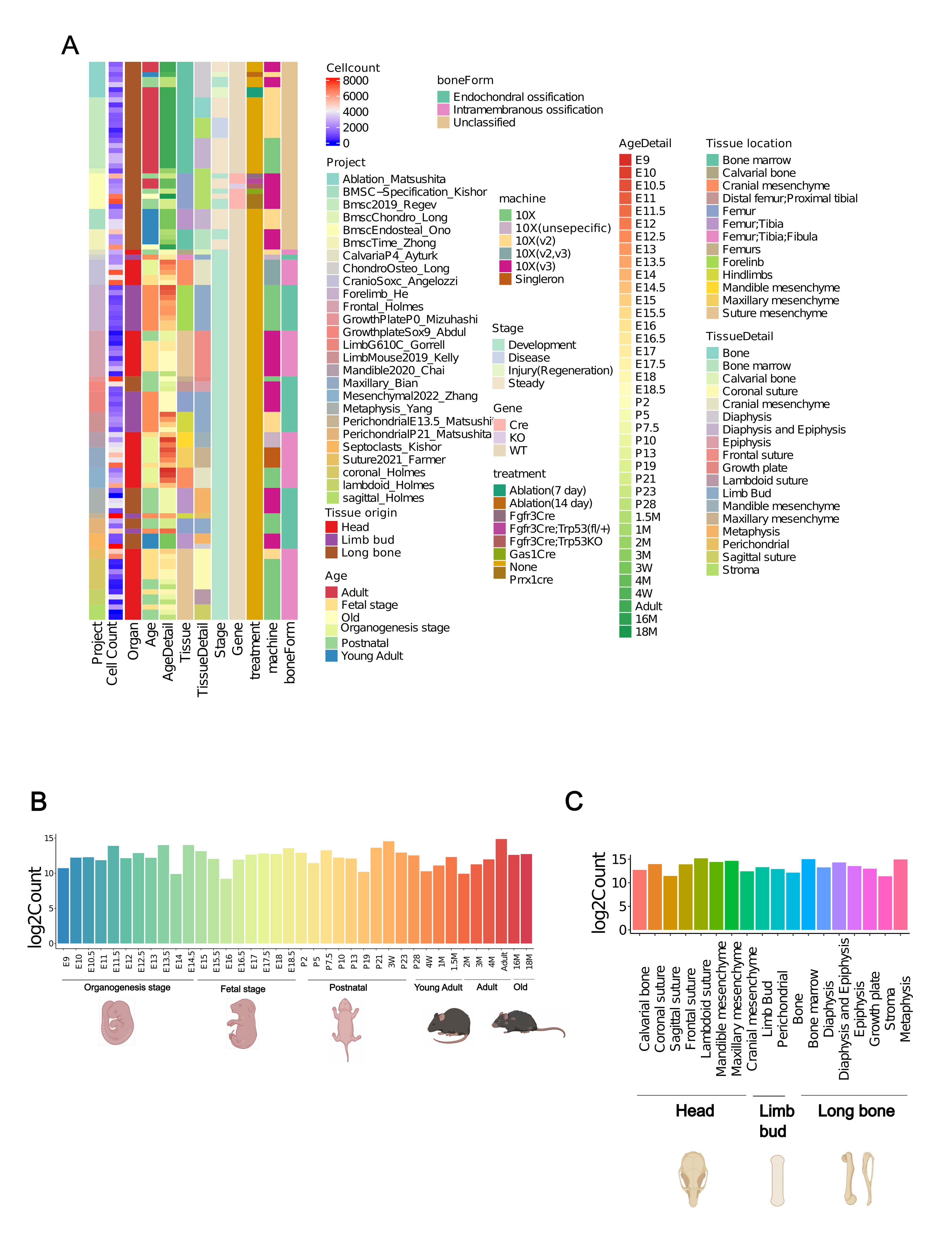

Supplement: S1 Fig — A, Heatmap visualization of cell count (second columns) and metadata of Differentiation Atlas. B,C, Barplot shows cell count (log2 scale) of (B) Age group and (C) tissue origin group. This figure was created with BioRender.com. (TIF) [file pgen.1011319.s001.tif]

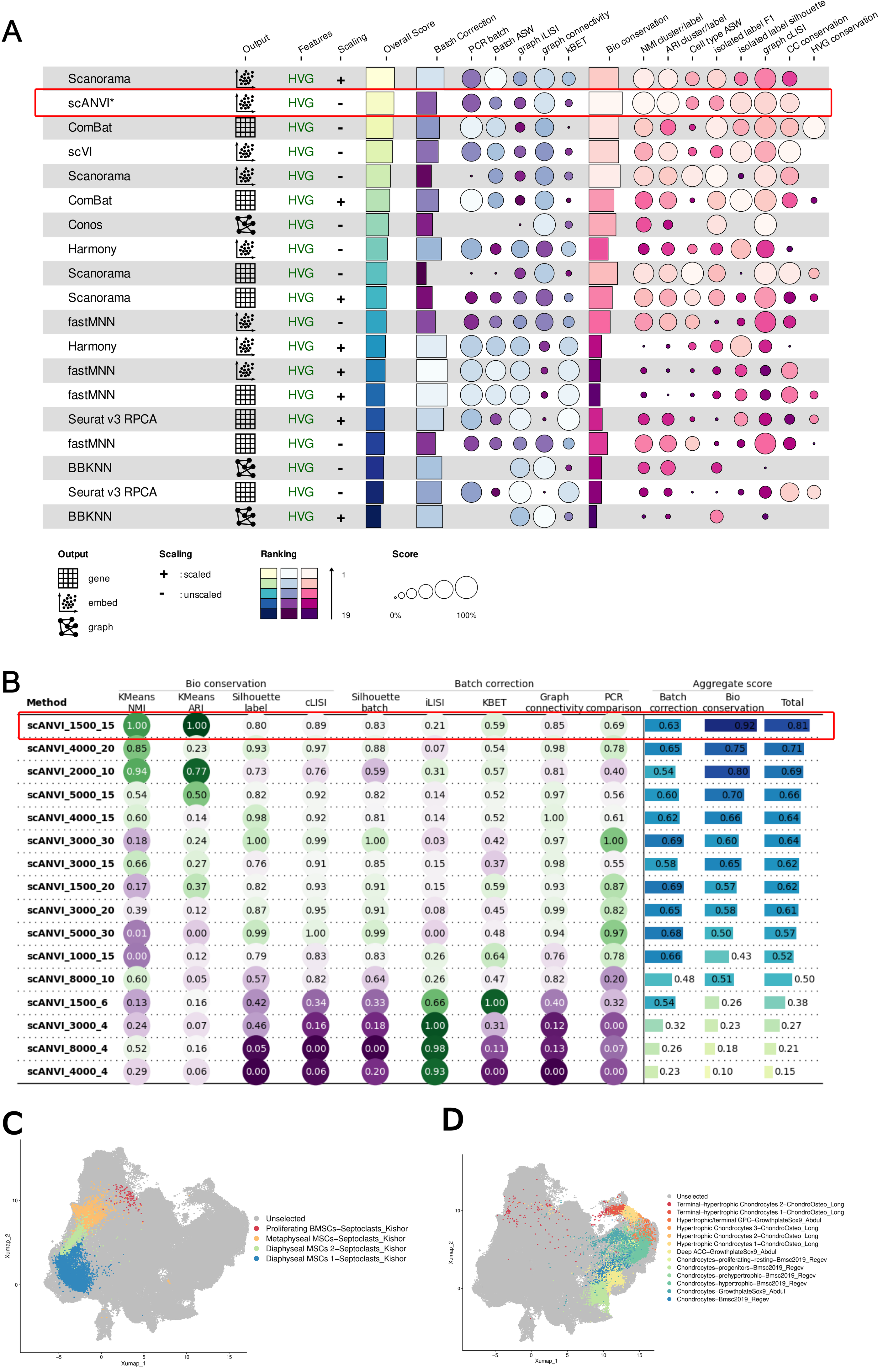

Supplement: S2 Fig — A, Result of data integration benchmarking. The rows represent methods tested, using a particular preprocessing and output. Preprocessing is summarized by "Scaling" (specifying whether or not gene values were scaled to mean 0 and standard deviation 1 across cells). Methods are sorted by overall score. The overall score is a weighted mean of the batch correction score and the bio-conservation score, which in turn are a mean of the individual metrics within the category. The output column specifies whether a method has corrected gene counts, an integrated embedding, or an integrated graph as output. B, Result of hyperparameters (HVG, n_latent) of scANVI benchmarking. The rows represent hyperparameters tested. C,D, Cell clusters annotated with previous studies are well separated with UMAP reduction in scANVI latent space. (TIF) [file pgen.1011319.s002.tif]

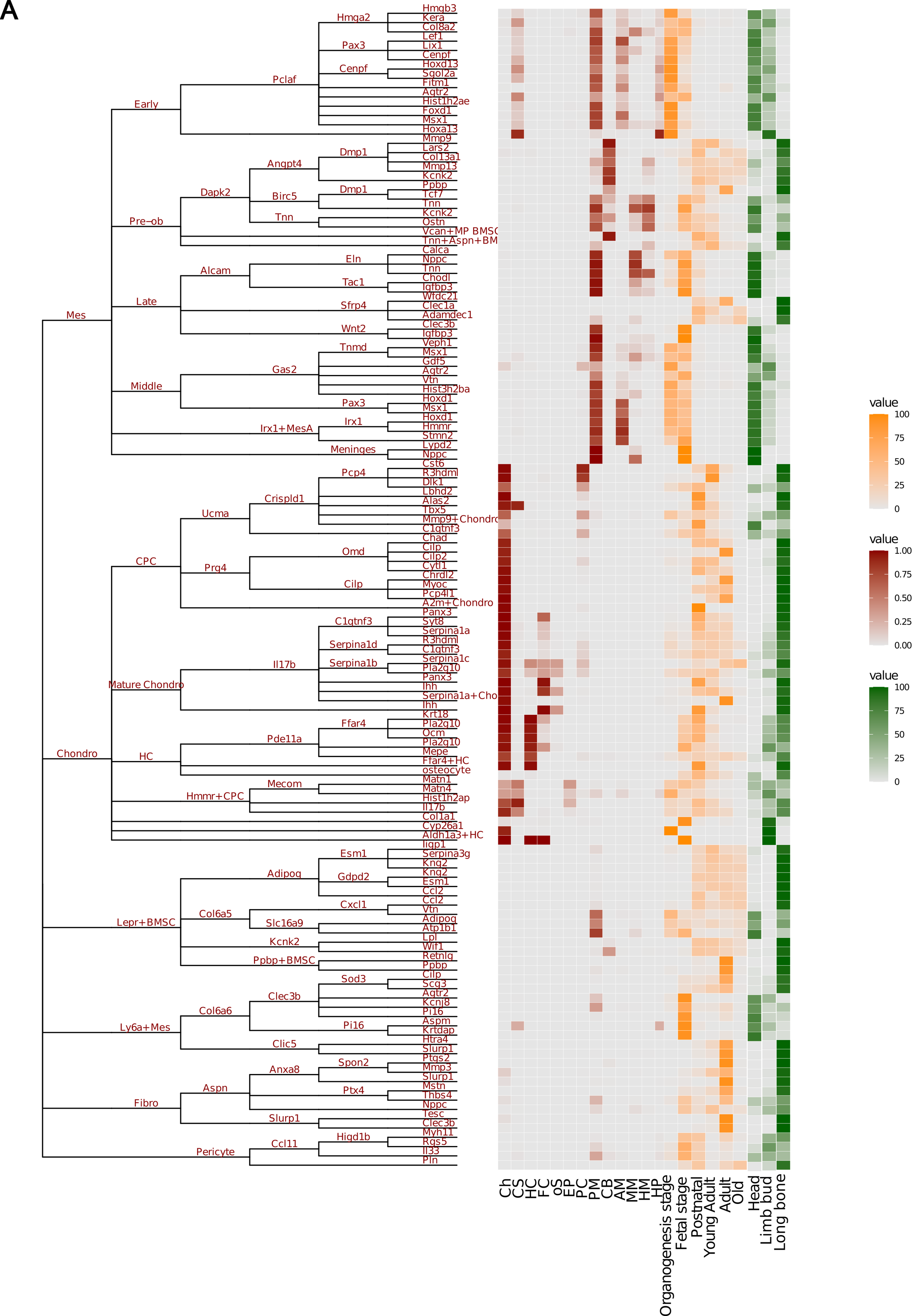

Supplement: S3 Fig — The first five levels with up to 49 clusters are presented, emphasizing the diverse nature of OPCs across various tissues and age groups. The left heatmap (red) depicts the overlapping of experimentally validated OPCs with clusters in the lowest tree level in the Differentiation Atlas. The middle heatmap (orange) depicts the relative percentage contribution of each cluster at the lowest tree level to the age group. The right heatmap (dark green) illustrates the relative percentage contribution of each cluster to the tissue origin group. (TIF) [file pgen.1011319.s003.tif]

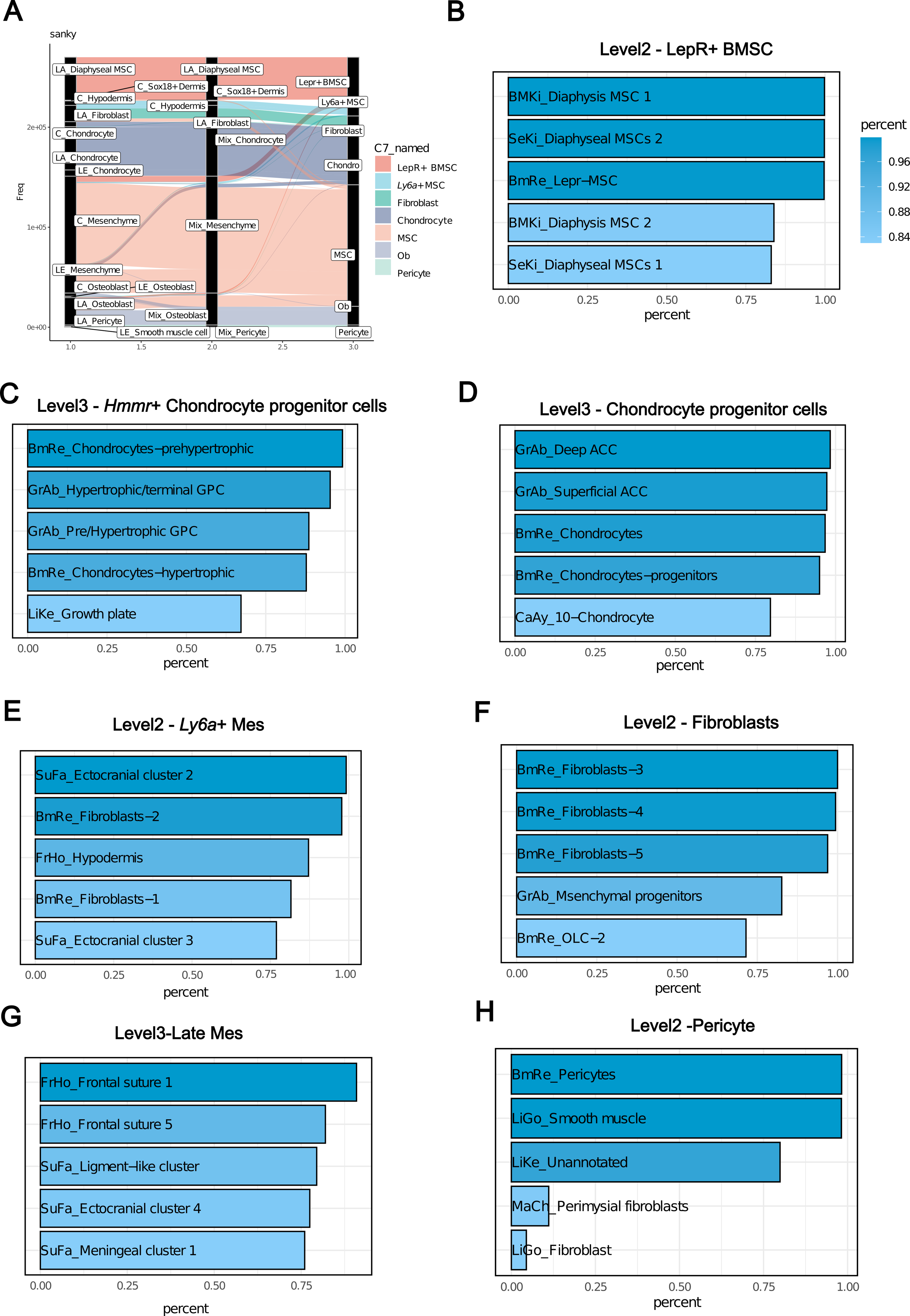

Supplement: S4 Fig — A, Sankey plot shows how level-2 annotations harmonize cell types from different tissues (LA: long bone; LE: Limb bud, C: Head). B-H, Barplot shows that level-2 annotations and level-3 annotations harmonize cell types from different studies. (TIF) [file pgen.1011319.s004.tif]

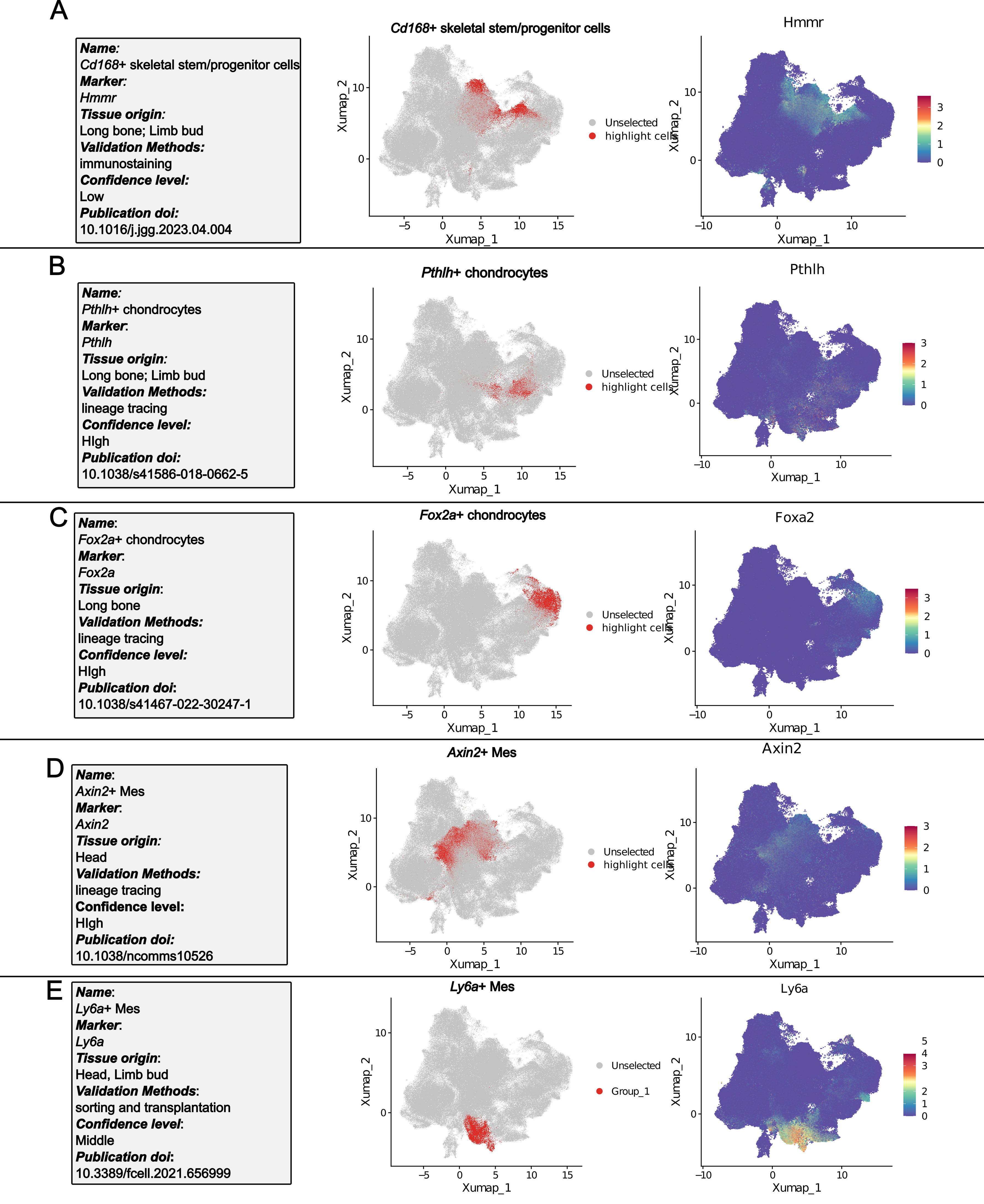

Supplement: S5 Fig — A-E, Examples of mapping experimentally validated OPCs to Differential Atlas. Left panels show the information on experimentally validated OPCs. The middle panels illustrate the cells mapped based on marker expression and tissue locations with UMAP visualization. The left panels show the expression of OPCs’ markers with UMAP visualization. (TIF) [file pgen.1011319.s005.tif]

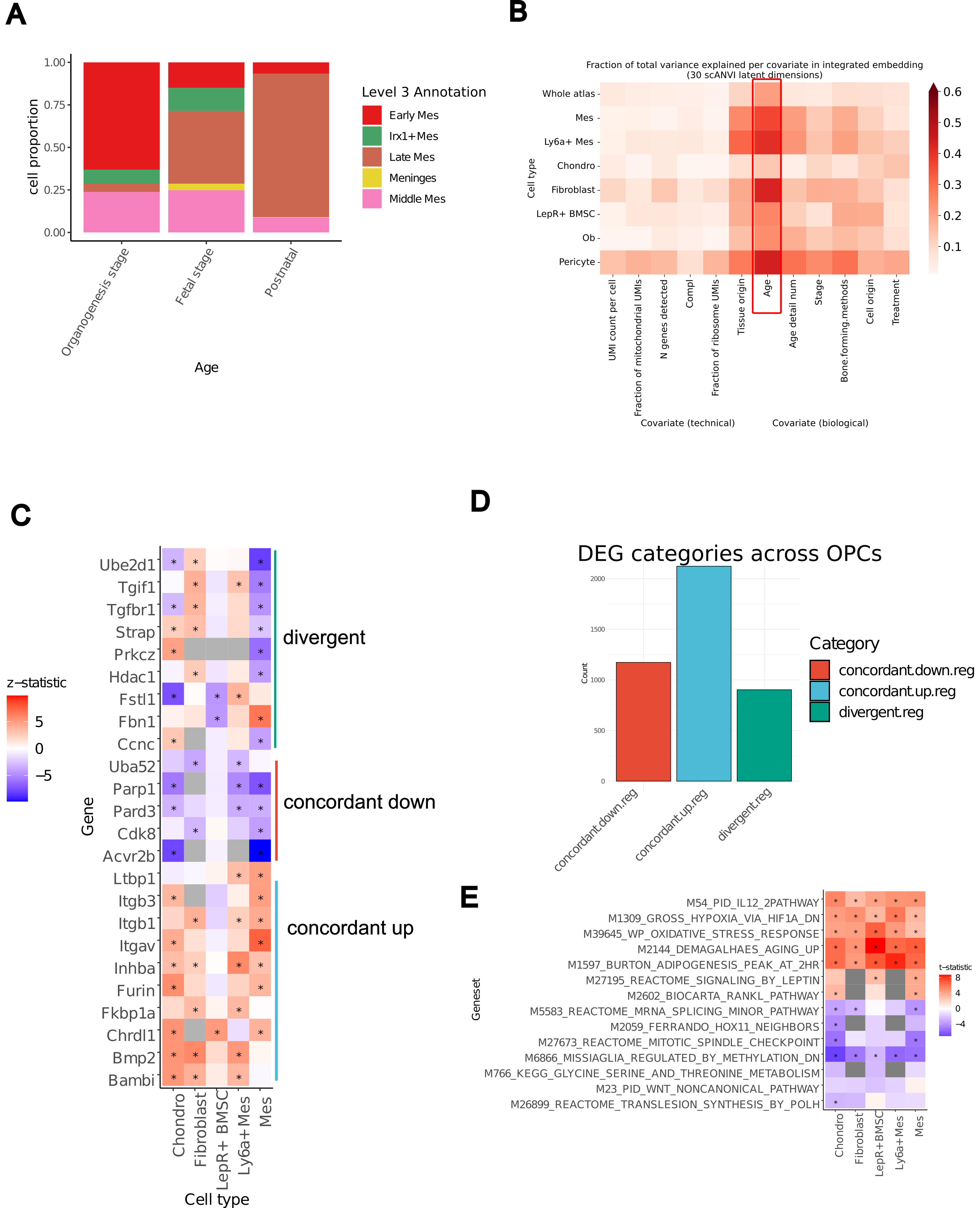

Supplement: S6 Fig — A, Barplots show the cell proportion of Mes subpopulations across different age groups. B, Fraction of total inter-sample variance in the Differential Atlas embedding that correlates with specific covariates. Covariates are split into technical (left) and biological covariates (right). Cell types at second annotation levels are shown. C, Heatmap shows differential expression z-statistic for genes in each cell cluster. ‘*’ indicates study-wide FDR < 5% in all panels. Grey boxs indicates a gene did not pass the expression cutoff in that cell cluster. Genes are categorized based on whether they are concordantly different across five OPCs. D, Barplot shows the number of genes in three categories in (C). E, Gene set analysis using the full spectrum of test statistics shows cell type conserved signatures Study-wide FDR < 0.05 is indicated by ‘*’. (TIF) [file pgen.1011319.s006.tif]

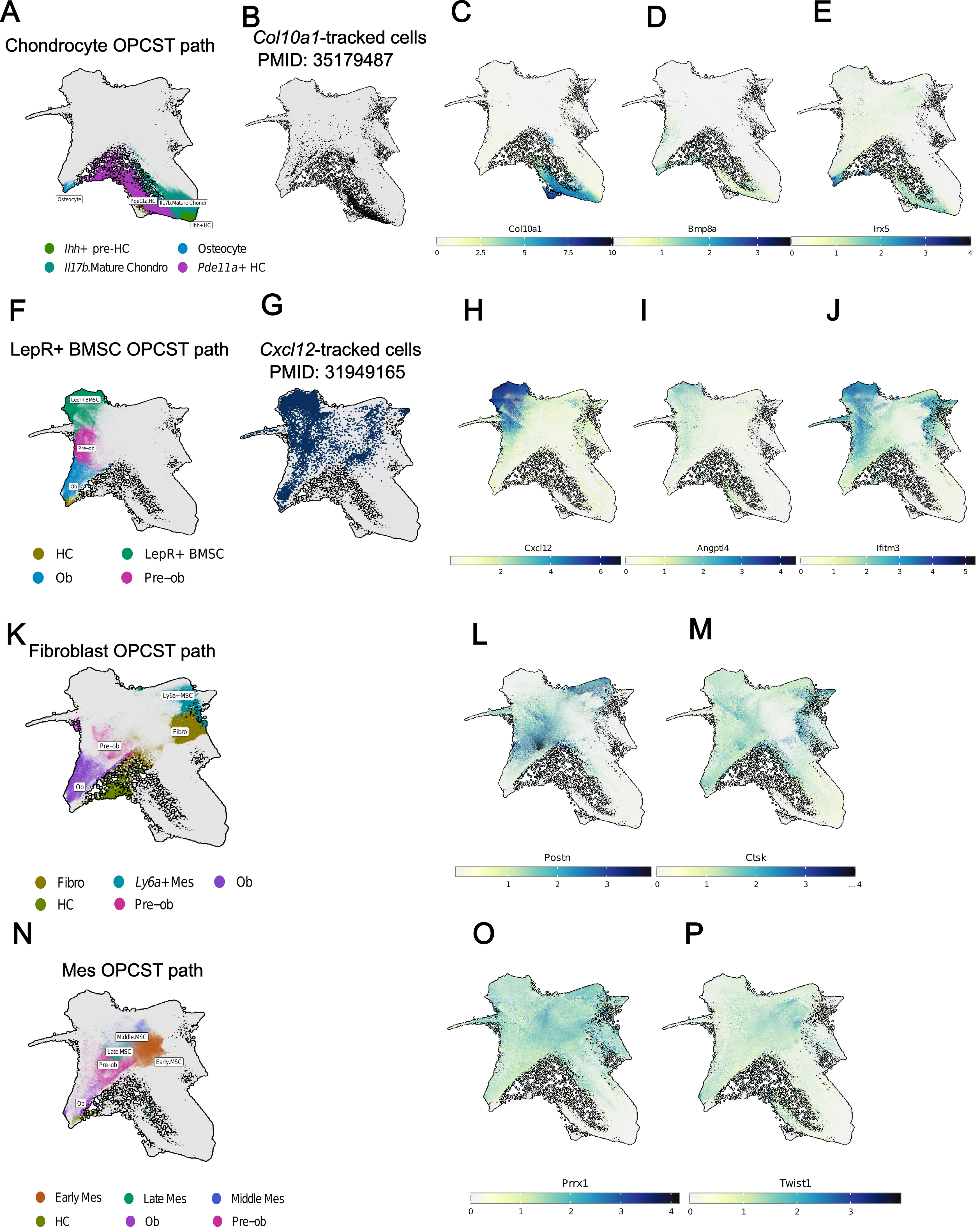

Supplement: S7 Fig — A,F,K,N, Force-directed graph visualization of differentiation path of (A) Chondrocyte OPCST, (F) LepR+ BMSC OPCST, (K) Fibroblast OPCST and (N) Mes OPCST. B,G, Force-directed graph highlighted lineage-tracked cells of (B) Col10a1, (G) Cxcl12, C-E,H,J,L-M,O-P Force-directed graph visualization of gene expression that is high along the differential path. (TIF) [file pgen.1011319.s007.tif]

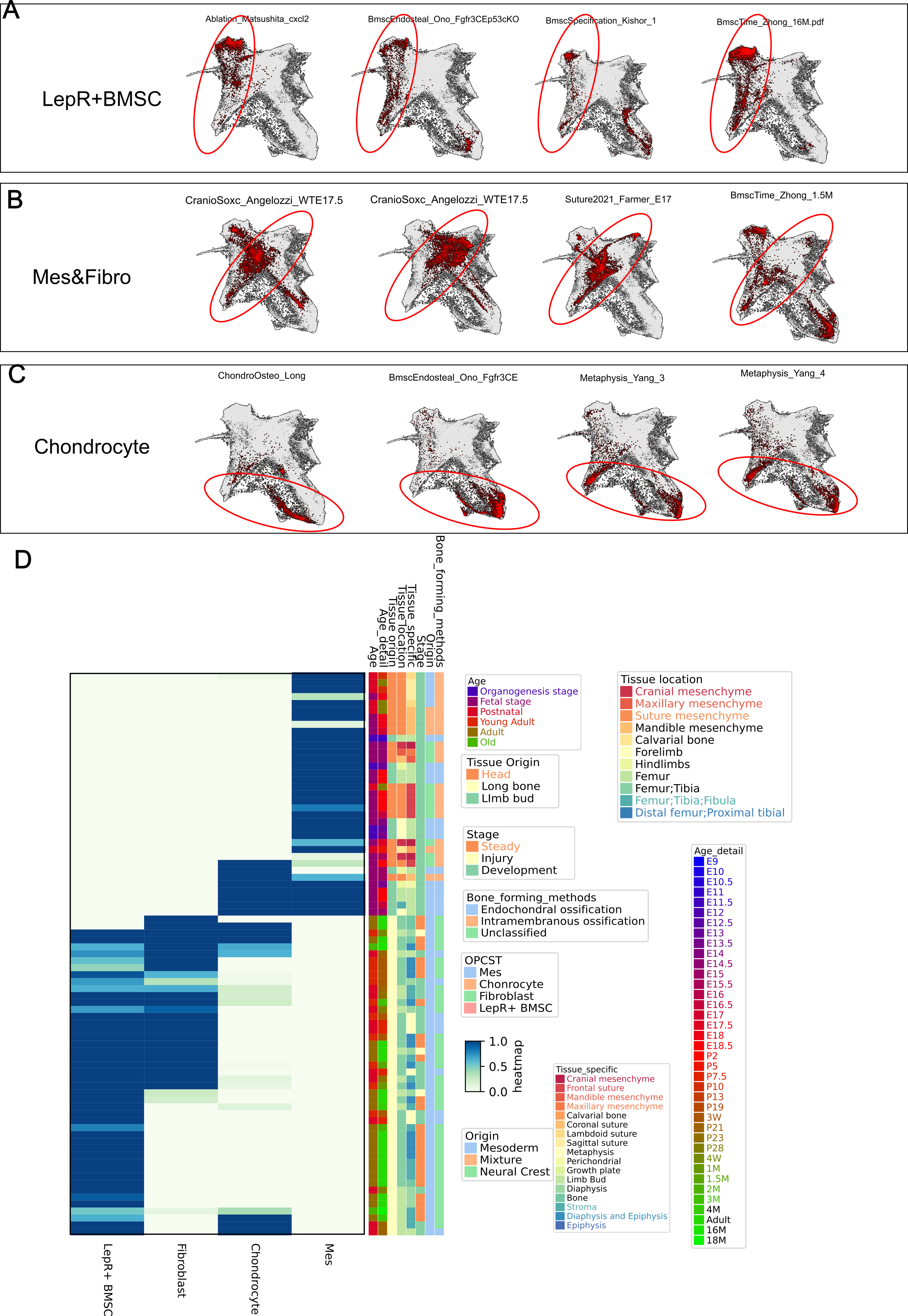

Supplement: S8 Fig — A-C, Transition processes from specific osteoprogenitors are similar across samples.D, Heatmap shows PAGA connectivity between four OPCs (column) and osteoblasts in samples (row). (TIF) [file pgen.1011319.s008.tif]

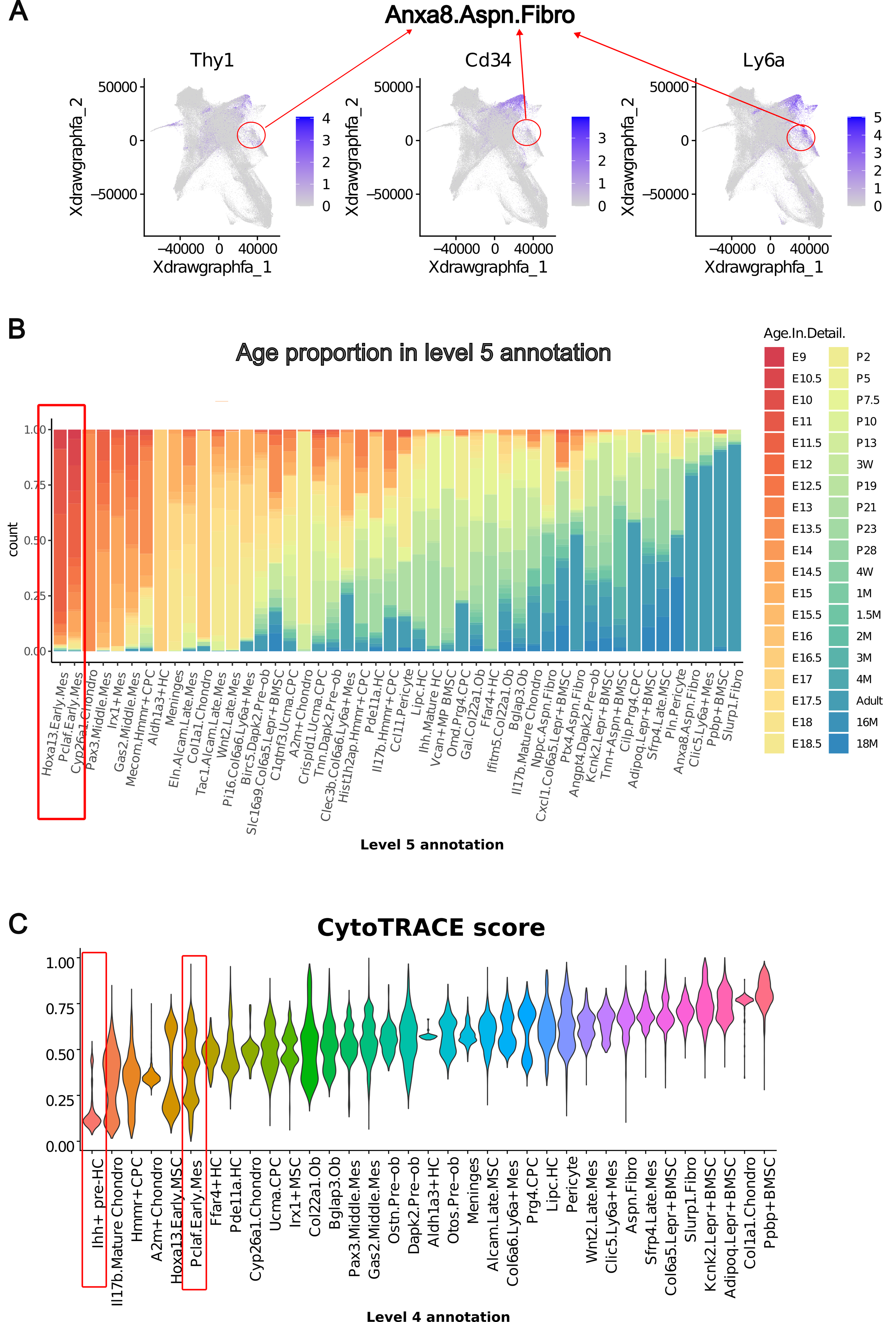

Supplement: S9 Fig — A, Expression of adult stem cell markers visualized with force-directed graph B, Barplot showing cell proportion in different ages across level-5 annotation C, Vlnplot shows the developmental potential predicted by CytoTRACE. Lower values indicate higher potential. (TIF) [file pgen.1011319.s009.tif]

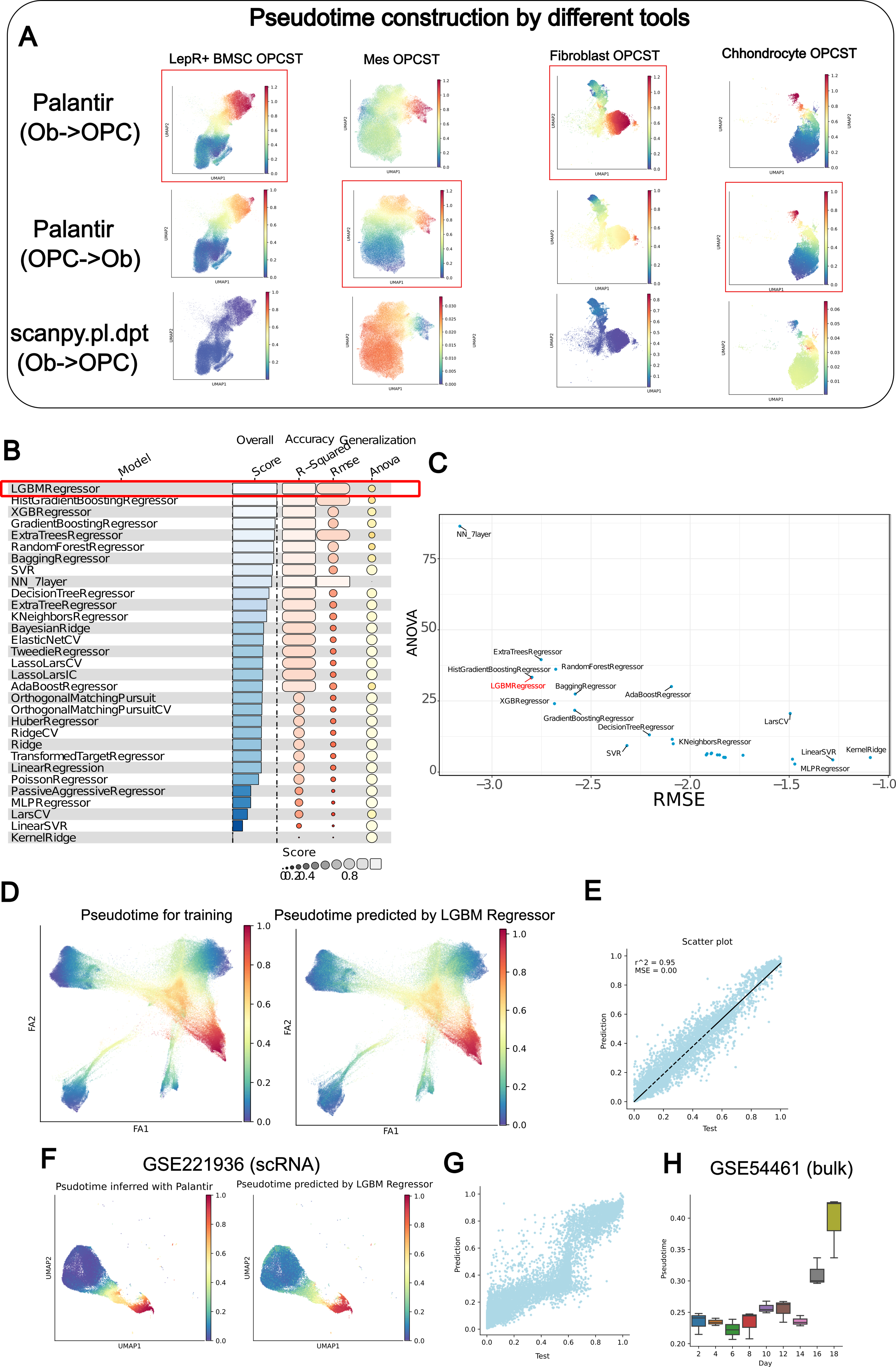

Supplement: S10 Fig — A, Expression of adult stem cell markers visualized with force-directed graph B, Barplot showing cell proportion in different ages across level-5 annotation C, Vlnplot shows the developmental potential predicted by CytoTRACE. Lower values indicate higher potential. (TIF) [file pgen.1011319.s010.tif]

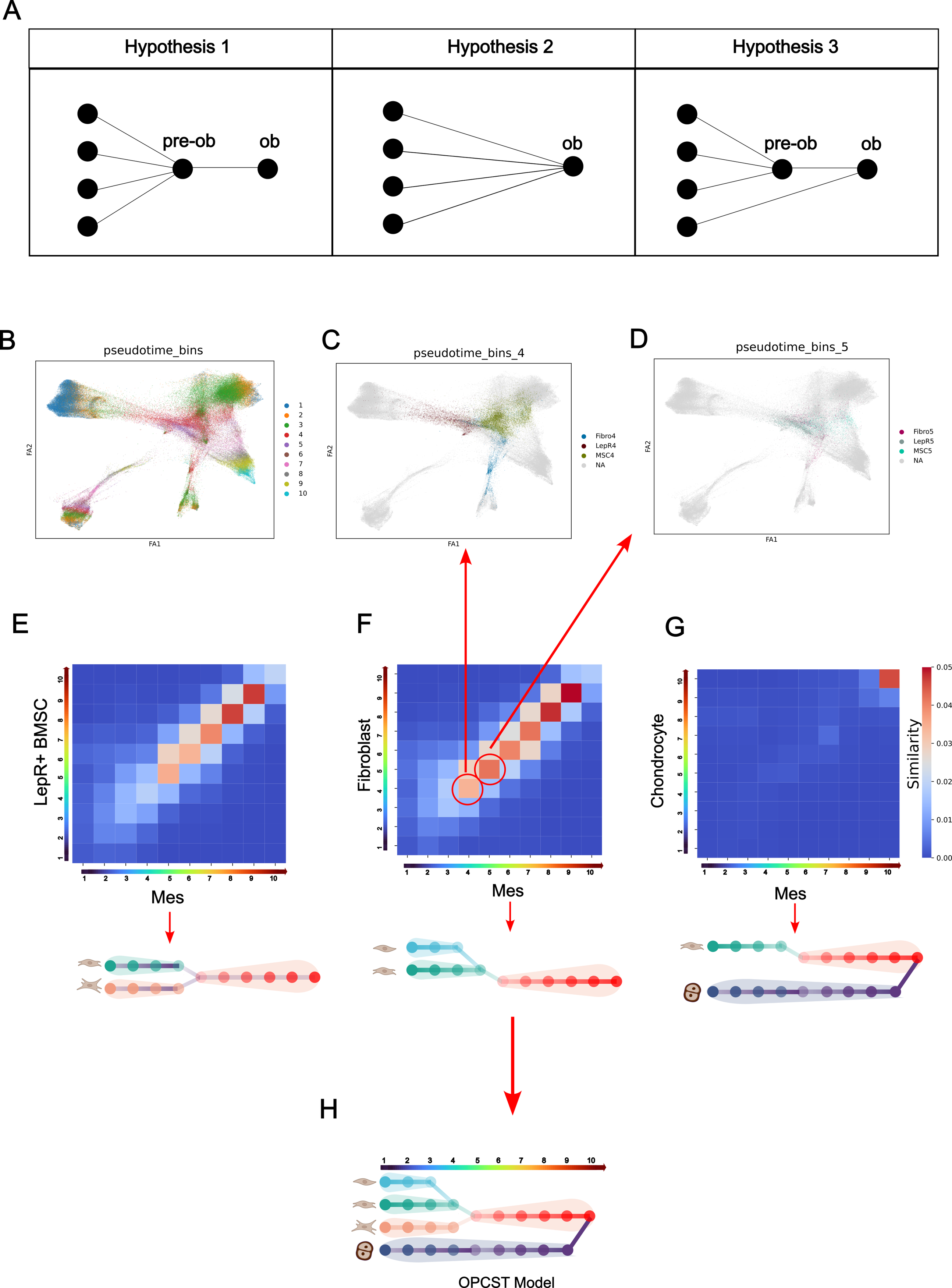

Supplement: S11 Fig — A,Diagram of the three hypotheses. B-D, Force-directed graph visualization colored by (B) 10 pseudotime bins, (C) pseudotime bin 4 in OPCSTs, and (D) pseudotime bin 5 in OPCSTs. E-G, Pseudotime bins across different OPCSTs with similar states are merged. H.,Final Differentiation Model inferred by (E-G). This figure was created with BioRender.com. (TIF) [file pgen.1011319.s011.tif]

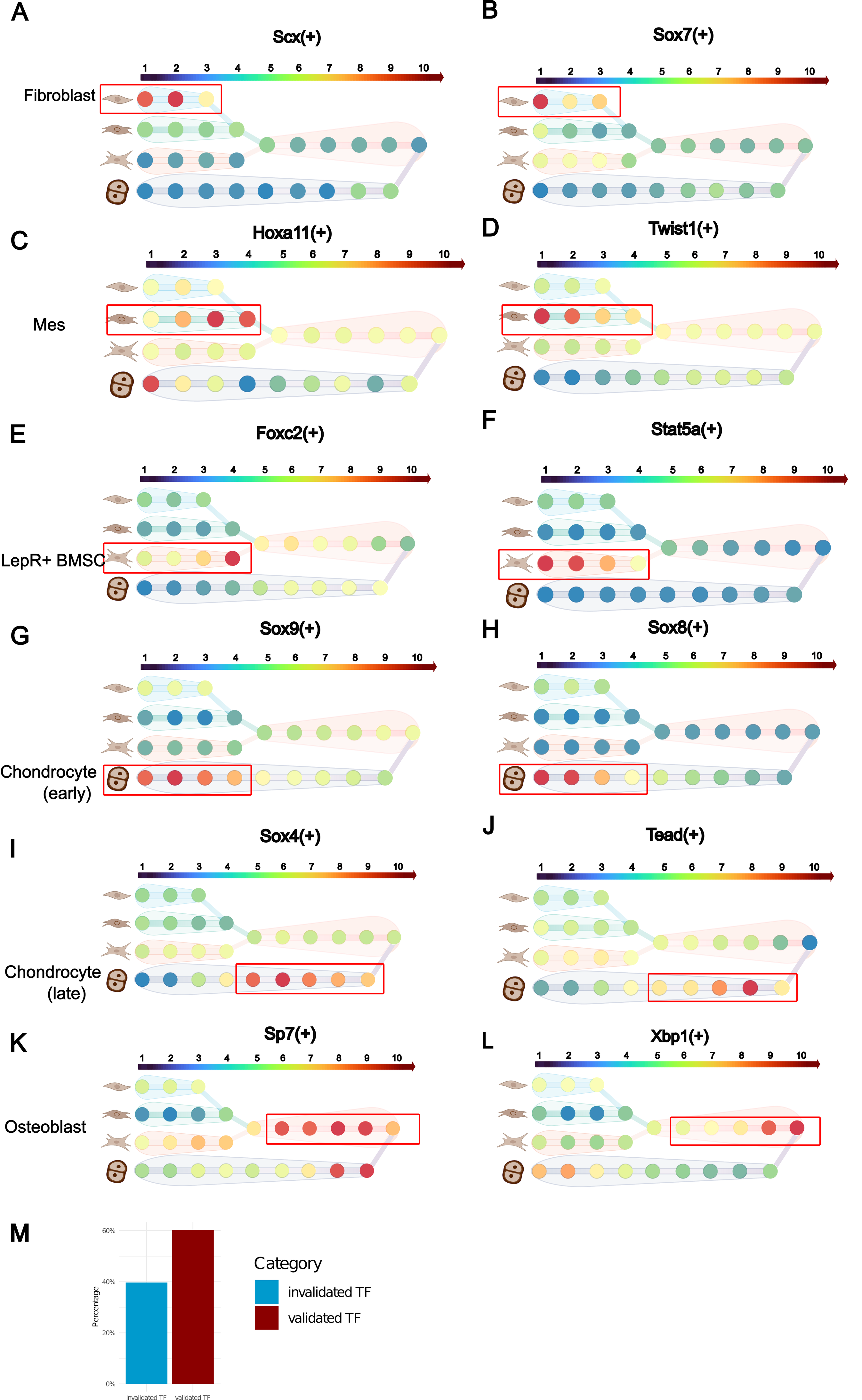

Supplement: S12 Fig — A-L, The left panel illustrates the activity of transcription factors across four OPCSTs. The colors represent pathway activity. The transcription factors to show were selected from Fig 3E. M, Barplot shows that most transcription factors identified in the Differentiation Model were validated to be related to bone formation (S5 Table). This figure was created with BioRender.com. (TIF) [file pgen.1011319.s012.tif]

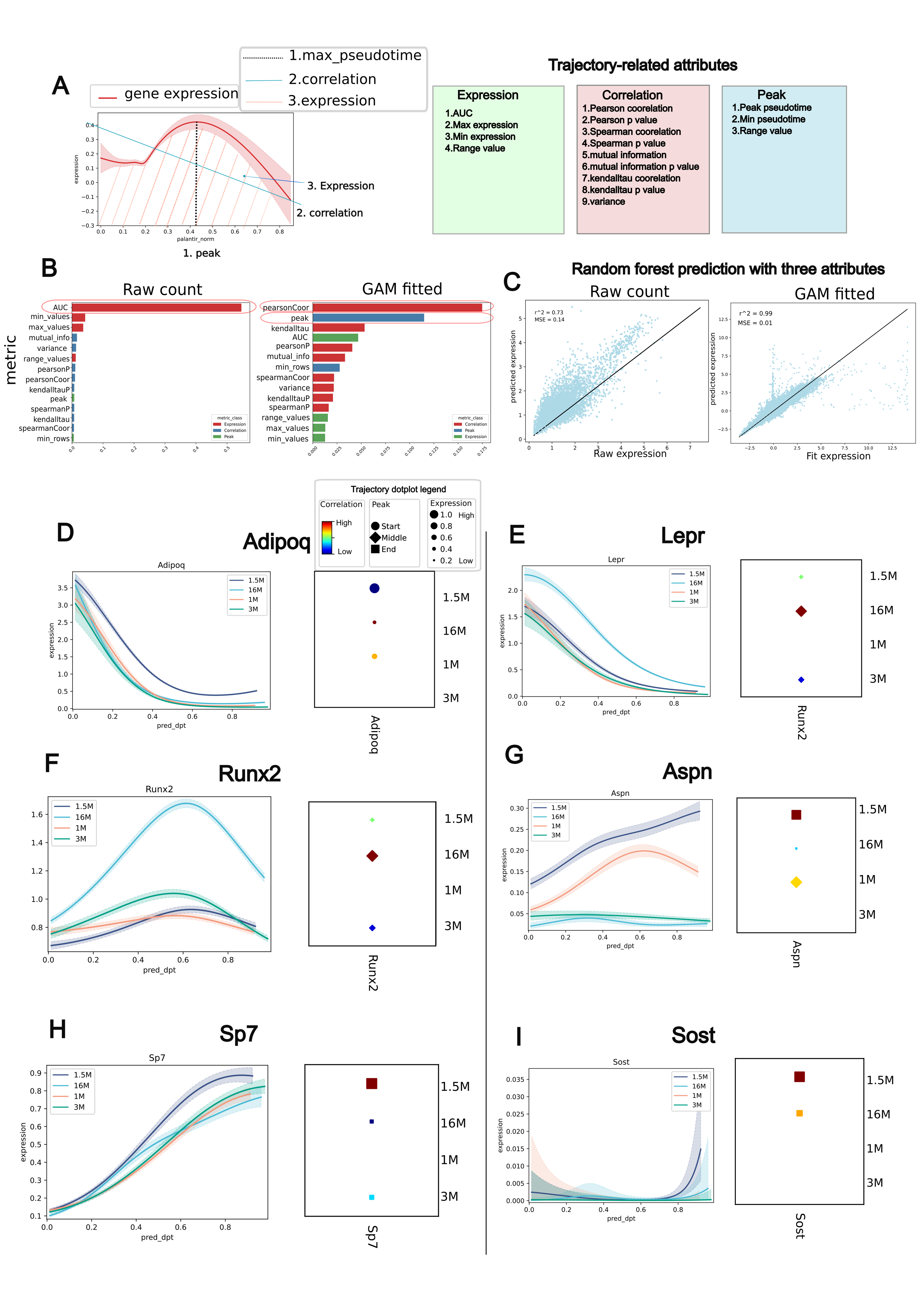

Supplement: S13 Fig — A, Sixteen attributes were extracted from gene expression along the pseudotime. B, The barplot shows the random forest-predicted importance of attributes in reconstructing gene expression. C, Scatter plot demonstrates that selected attributes can reconstruct GAM-fitted expression (left) and raw count (right). D-I, Trajectory dotplot intuitively reflects the different pseudotemporal expression patterns of genes. Pseudotemporal gene expression was visualized with CellRank (left panel) and trajectory dotplot (right panel). (TIF) [file pgen.1011319.s013.tif]

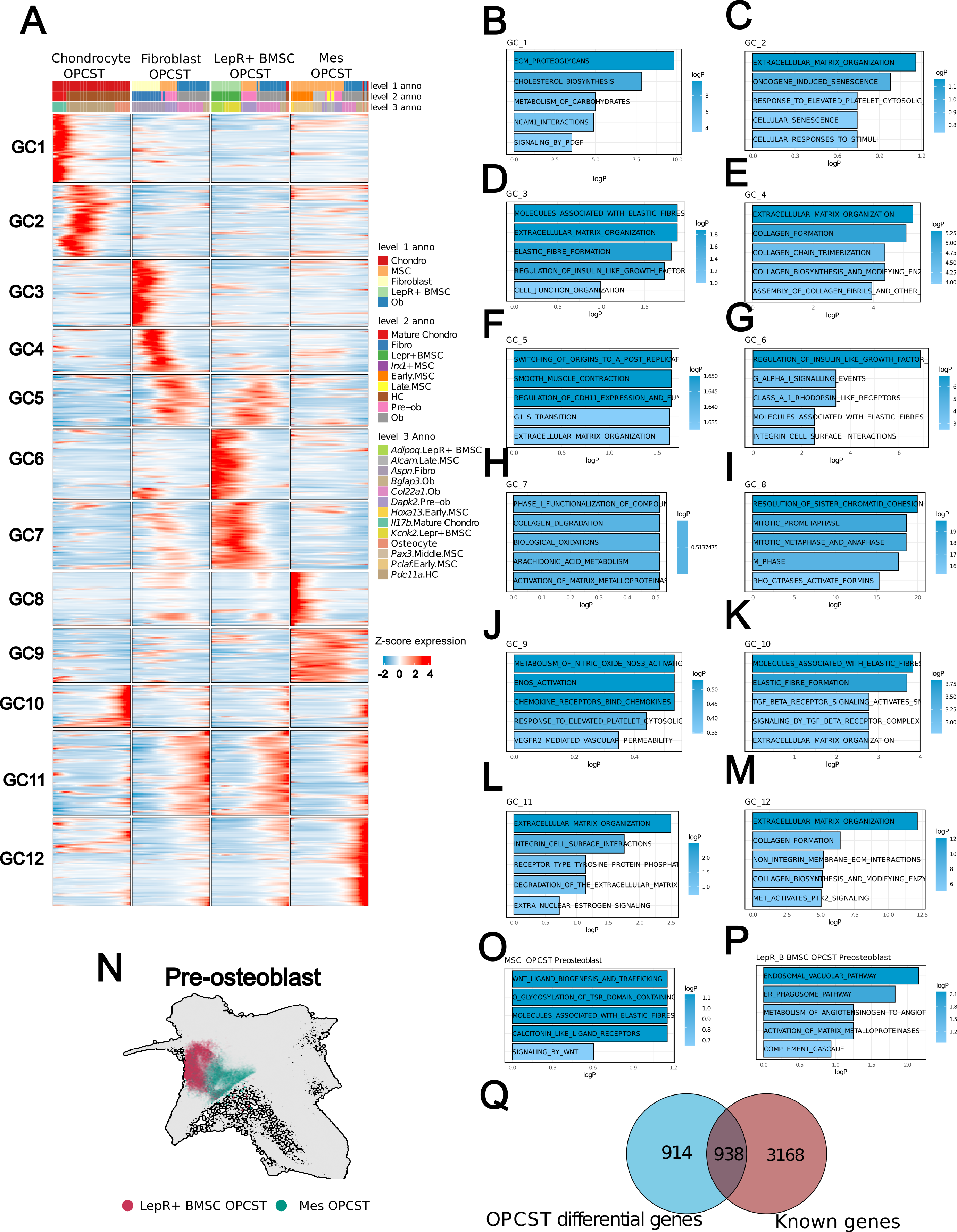

Supplement: S14 Fig — A, Multiway heatmap of changes of pseudotemporal gene expression for four OPCST. Genes to show were selected with the associationTest procedure from tradeSeq. Gene clusters were grouped by k-means. B-M, The barplots shows the top five Reactome enrichment results of gene clusters in (a) arranged by p-value. N, Pre-osteoblasts between Lepr+ BMSC OPCST and Mes OPCST exhibit different states. O,P, Barplots show the top five Reactome enrichment results of differential genes between two states of pre-osteoblast in (N) arranged by p-value Q, Venn plot shows the major proportion of differential genes identified in Differentiation Models are annotated in other bone databases (S6 Table). (TIF) [file pgen.1011319.s014.tif]

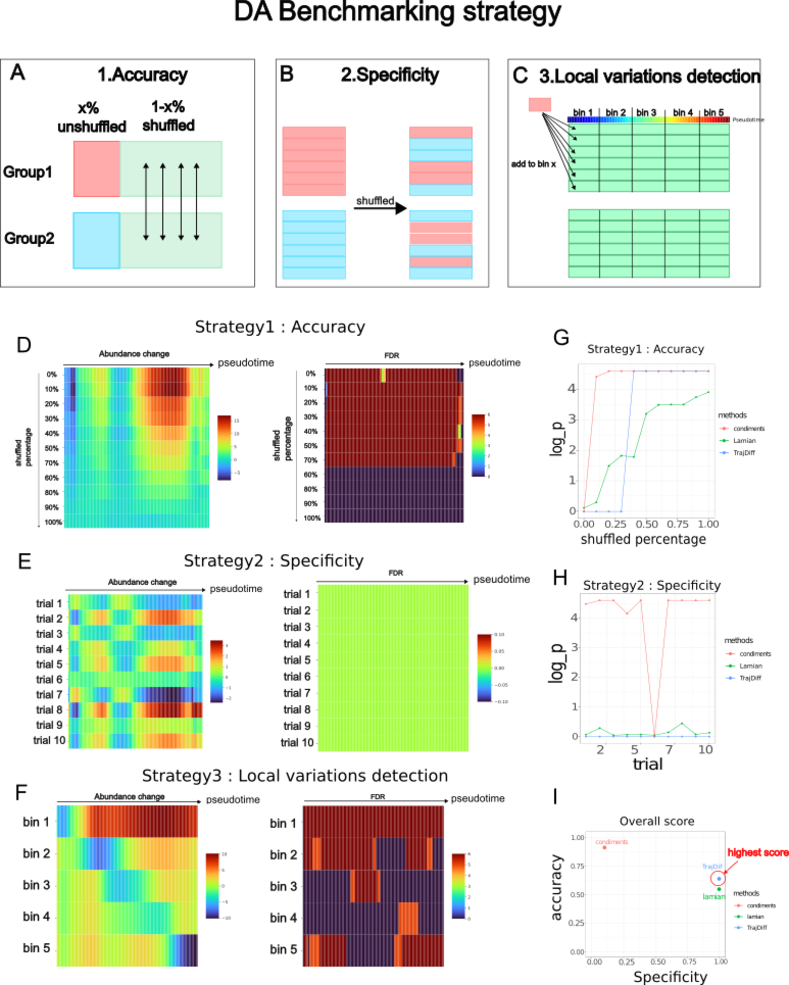

Supplement: S15 Fig — A-C, Overview of benchmarking strategy for (A) accuracy, (B) specificity, and (C) local variation detection. D, Heatmap reveals that TrajDiff can identify subtle changes in cell abundance. E, Heatmap reveals that TrajDiff exhibits robustness to false positives. F, Heatmap reveals that TrajDiff identifies local variations in cell abundance. G-I, TrajDiff demonstrates both high accuracy and specificity compared to Lamian and Condiment. (TIF) [file pgen.1011319.s015.tif]

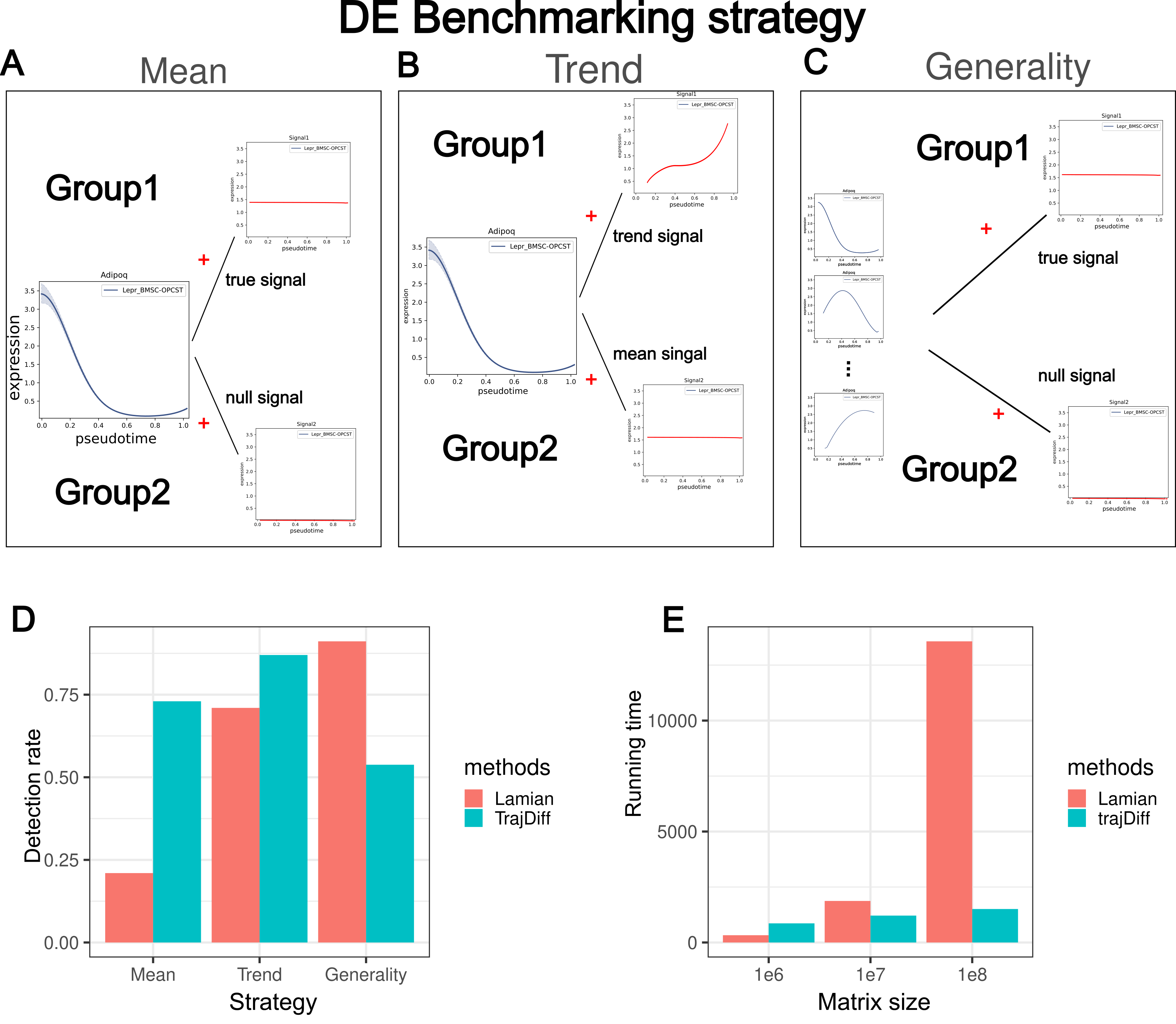

Supplement: S16 Fig — A-C, Overview of benchmarking strategy for (A) mean difference, (B) trend difference, and (C) generality. B, TrajDiff performs better in detecting mean difference and trend difference than Lamian. C, TrajDiff takes significantly less time compared to Lamian. (TIF) [file pgen.1011319.s016.tif]

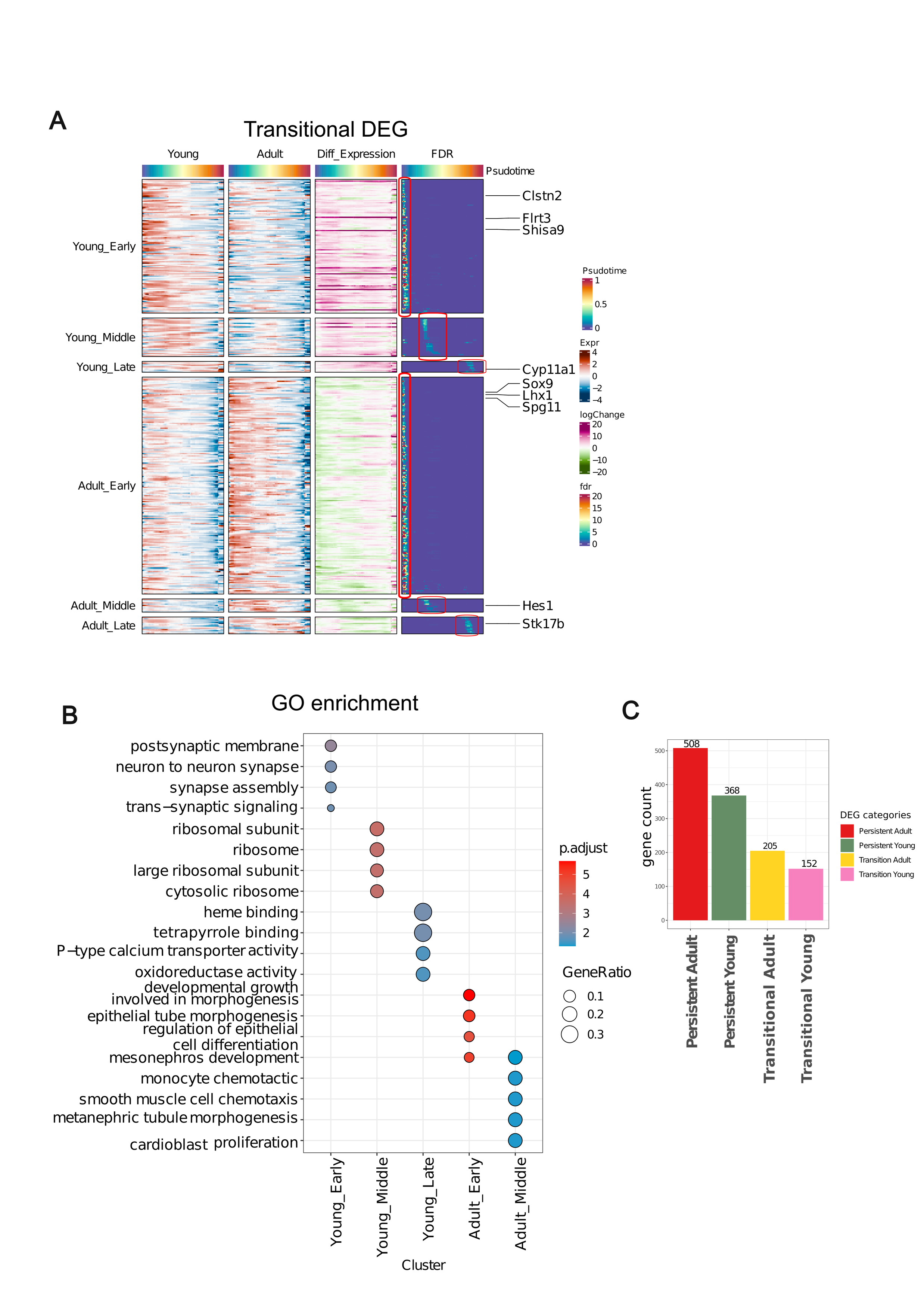

Supplement: S17 Fig — A, Heatmaps are presented in four vertical panels to illustrate transit differential genes expression of the Young group (first panel) and the Adult group (second panel), expression differences between the two groups (third panel), and FDR (fourth panel). In each panel, rows represent genes, while columns represent pseudotime. Genes are categorized into 8 clusters based on their expression patterns. B, GO enrichment of six gene clusters in (A), visualized with dotplot. C, The number of genes in different differential patterns (persistent or transitional). (TIF) [file pgen.1011319.s017.tif]

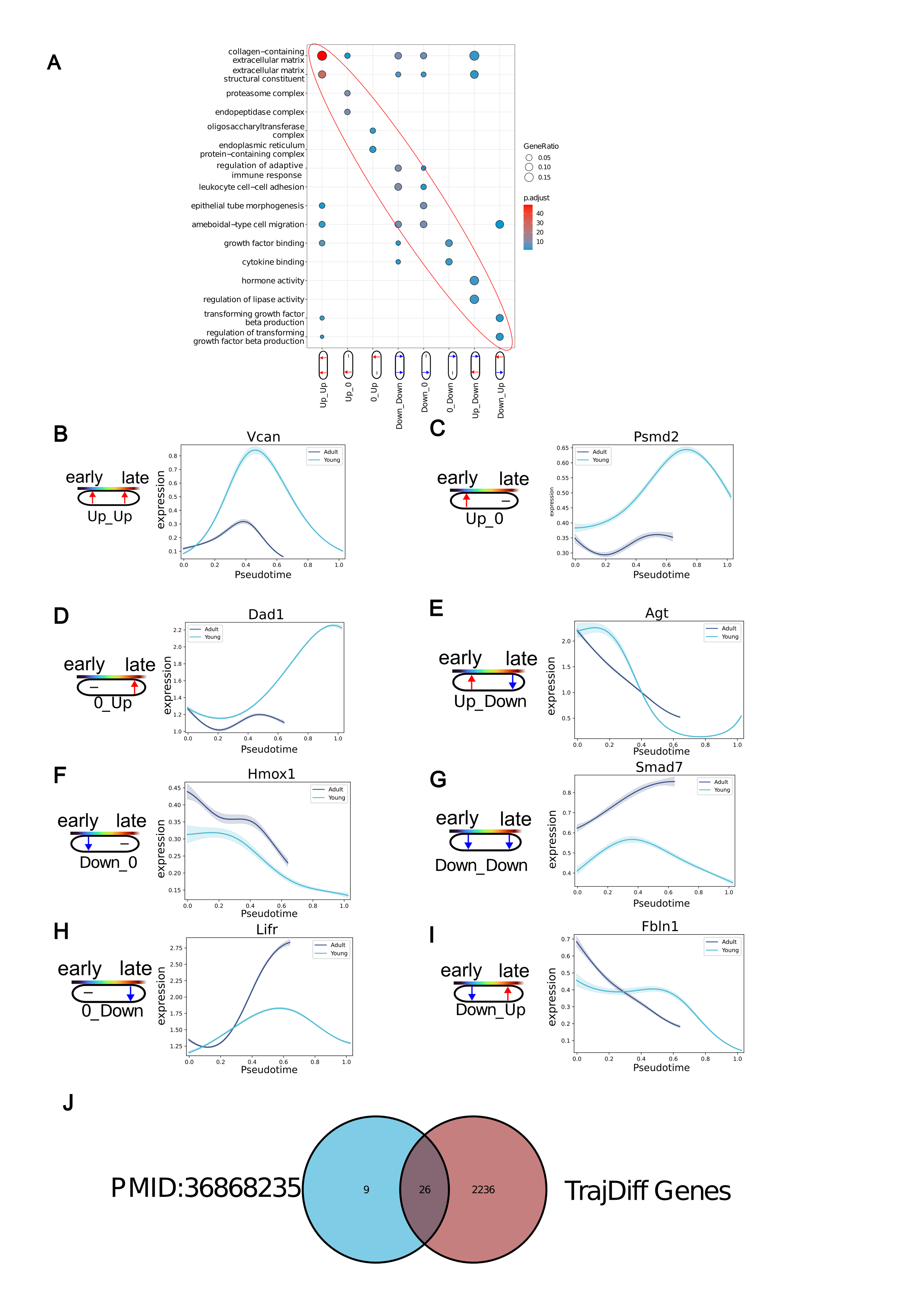

Supplement: S18 Fig — A, GO enrichment of seven clusters in Fig 4D, visualized with dotplot. B-I, Pseudotemporal gene expression visualized with CellRank to validate differential genes in Fig 4E. J, Venn plot shows the overlap between differential genes in TrajDiff and differential genes provided in the previous study. (TIF) [file pgen.1011319.s018.tif]

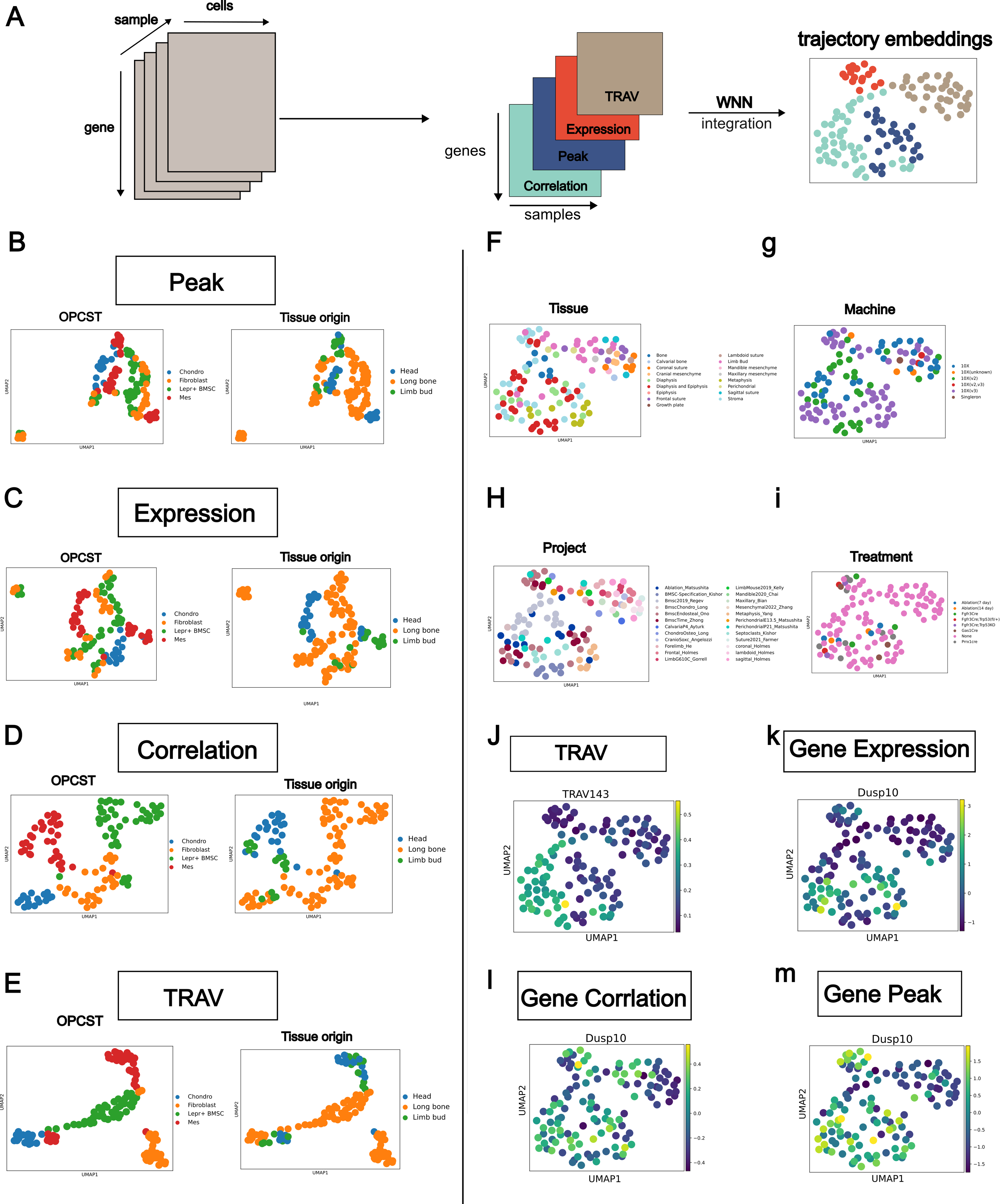

Supplement: S19 Fig — A, A schematic for trajectory reduction. TRAV activity matrix, gene expression, gene peak, and gene correlation were treated as four modalities of trajectories, which were integrated by weighted nearest neighbor (WNN) to get trajectory embeddings. B-E, Independent analysis of (B) peak, (C) expression, (D) correlation, and (E) TRAV modalities. F-I, Trajectory reduction after integrating four modalities with WNN, colored by (F) Tissue location, (G) Machine, (H) Project, (I) Treatment. J-M, Trajectory reduction enables visualization of (J) TRAV activities, (K) gene expression, (L) gene correlation, and (M) gene peak. (TIF) [file pgen.1011319.s019.tif]

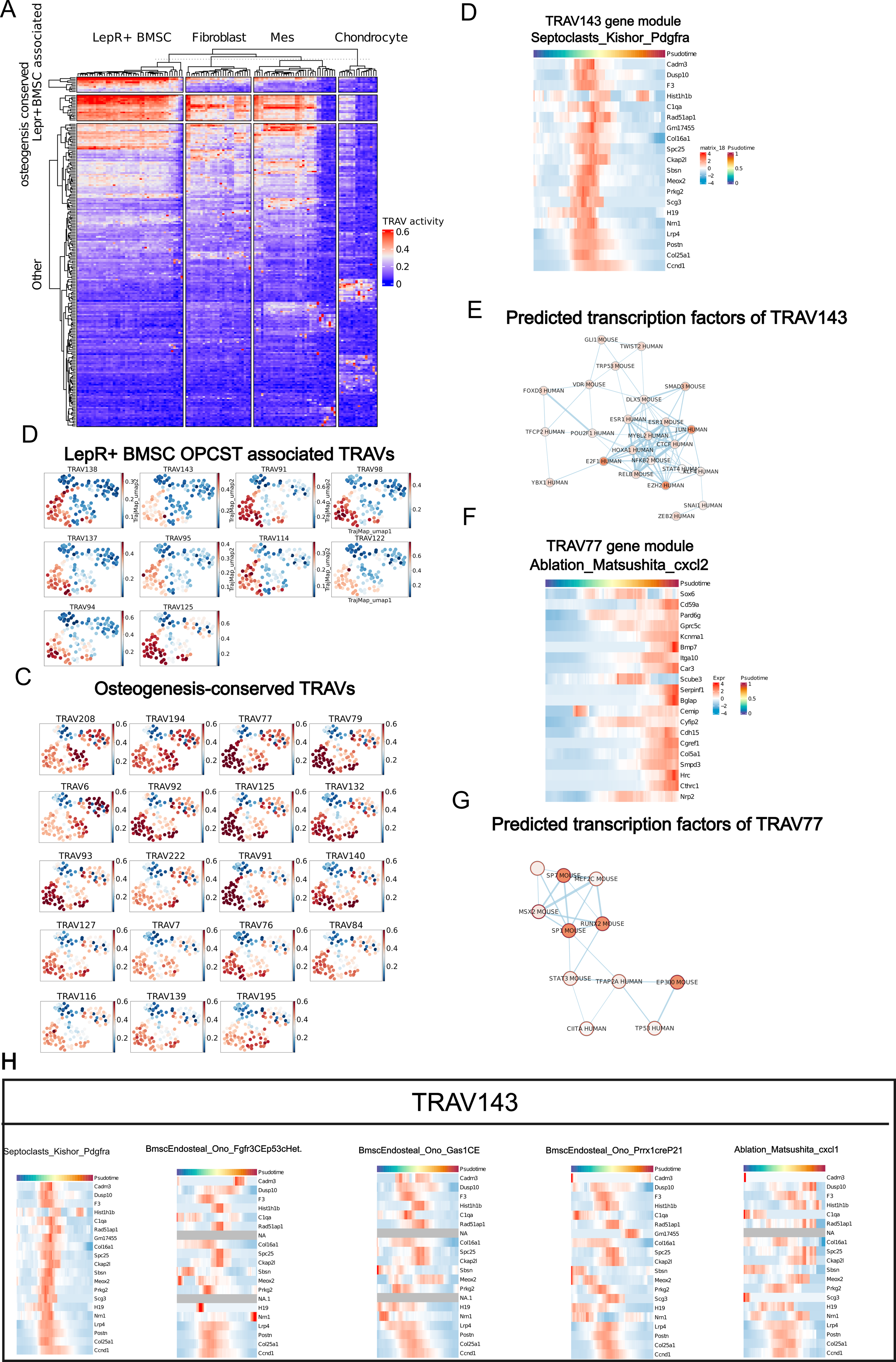

Supplement: S20 Fig — a, Heatmap shows activity of TRAVs (row) across trajectories (column) of four OPCST. b,c, TRAV activity visualized with trajectory embeddings, colored by (b) LepR+ BMSC OPCST TRAVs, (c) osteogenesis-conserved TRAVs. d,f, Heatmap shows pseudotemporal gene expression of (d) TRAV143 and (f) TRAV77 in the sample with the highest TRAV activity. e,f, Predicted transcription factors that regulate (e) TRAV143, (g) TRAV77. h, Heatmaps shows shows conserved pseudotemporal gene expression of TRAV143 in multiple samples. (TIF) [file pgen.1011319.s020.tif]

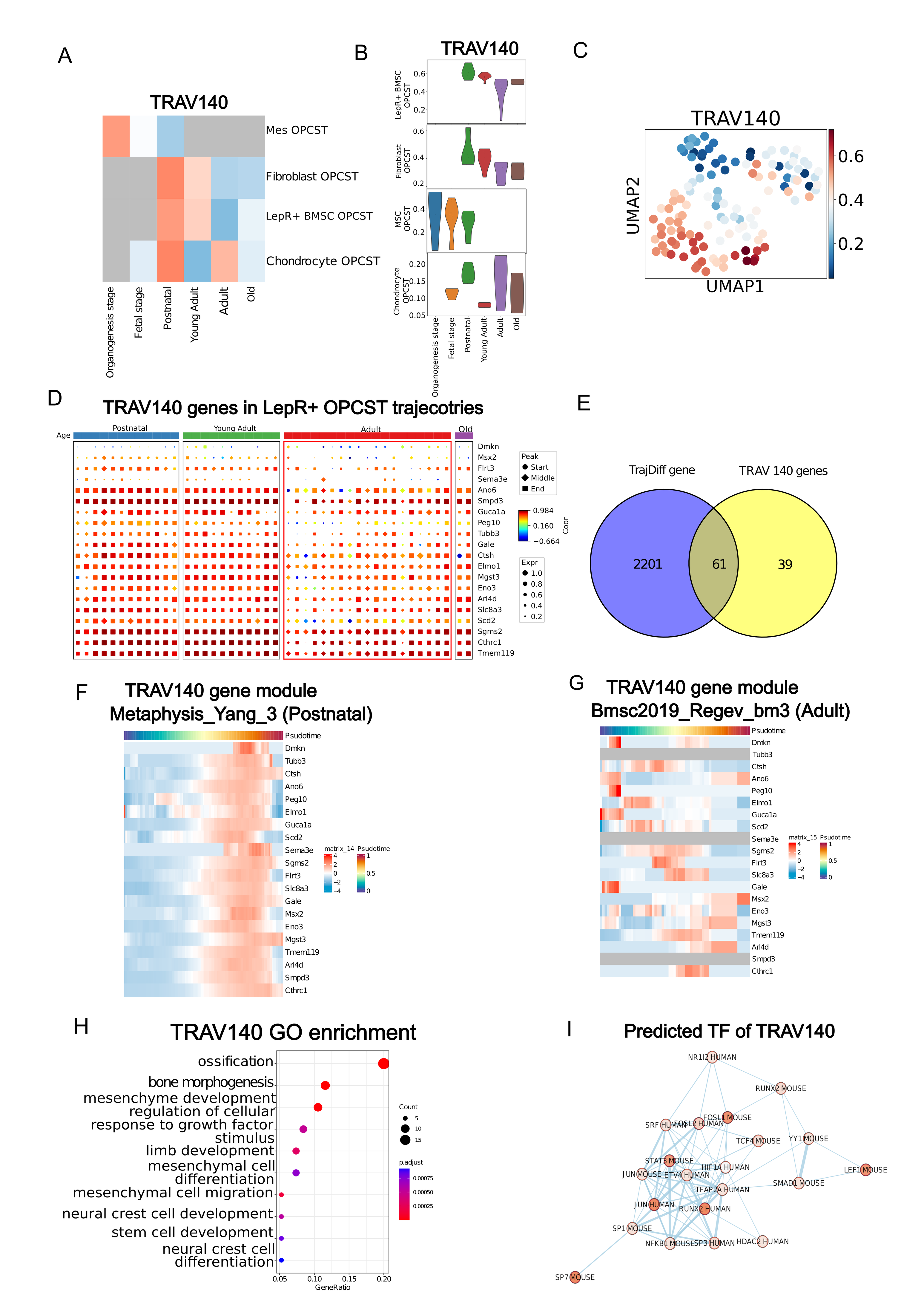

Supplement: S21 Fig — A, Heatmap shows the activity of TRAV140 across OPCSTs (row) and Ages (column). B, Violin plots show TRAV activity across OPCSTs and Ages (row). C, TRAV140 activity visualized with trajectory embeddings. D, Trajectory dotplot illustrates the genes expression patterns of the TRAV140 gene module (row) across LepR+ OPCST trajectories (column). E, Venn plot shows overlapping between genes in TRAV140 gene module and genes identified with TrajDiff in Fig 4D. F,G, Heatmap shows distinct pseudotemporal gene expression of TRAV140 gene module in trajectories in (F) postnatal group and (G) adult group. H, GO enrichment of TRAV140 gene module, visualized with dotplot. i, Predicted Transcript factors that regulate TRAV140. (TIF) [file pgen.1011319.s021.tif]

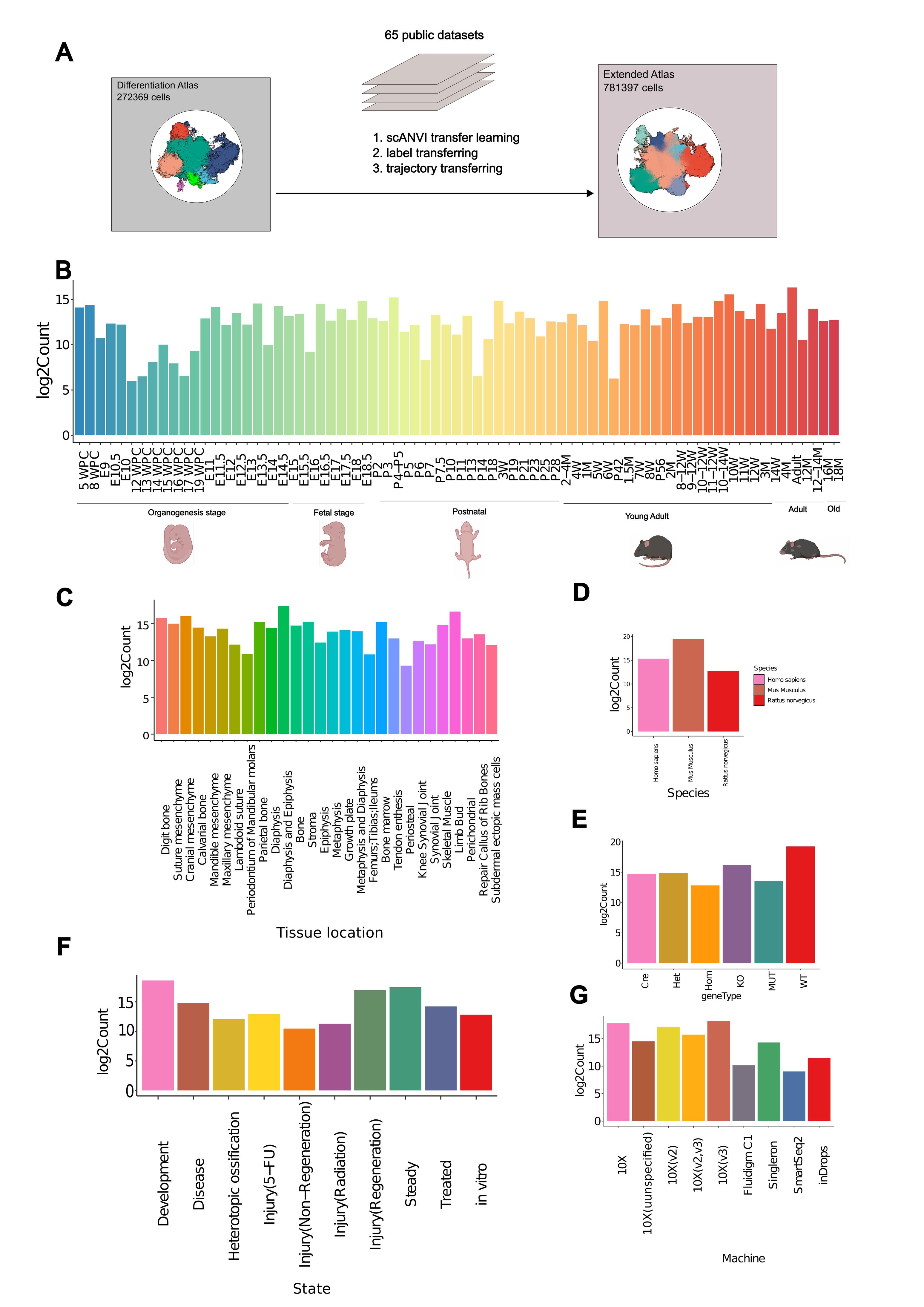

Supplement: S22 Fig — A-F, Barplots show cell count (log2 scale) of (A) Age group, (B) Tissue location group, (C) Species group, (D) State group, (E) Gene type group, and (F) Machine group. This figure was created with BioRender.com. (TIF) [file pgen.1011319.s022.tif]

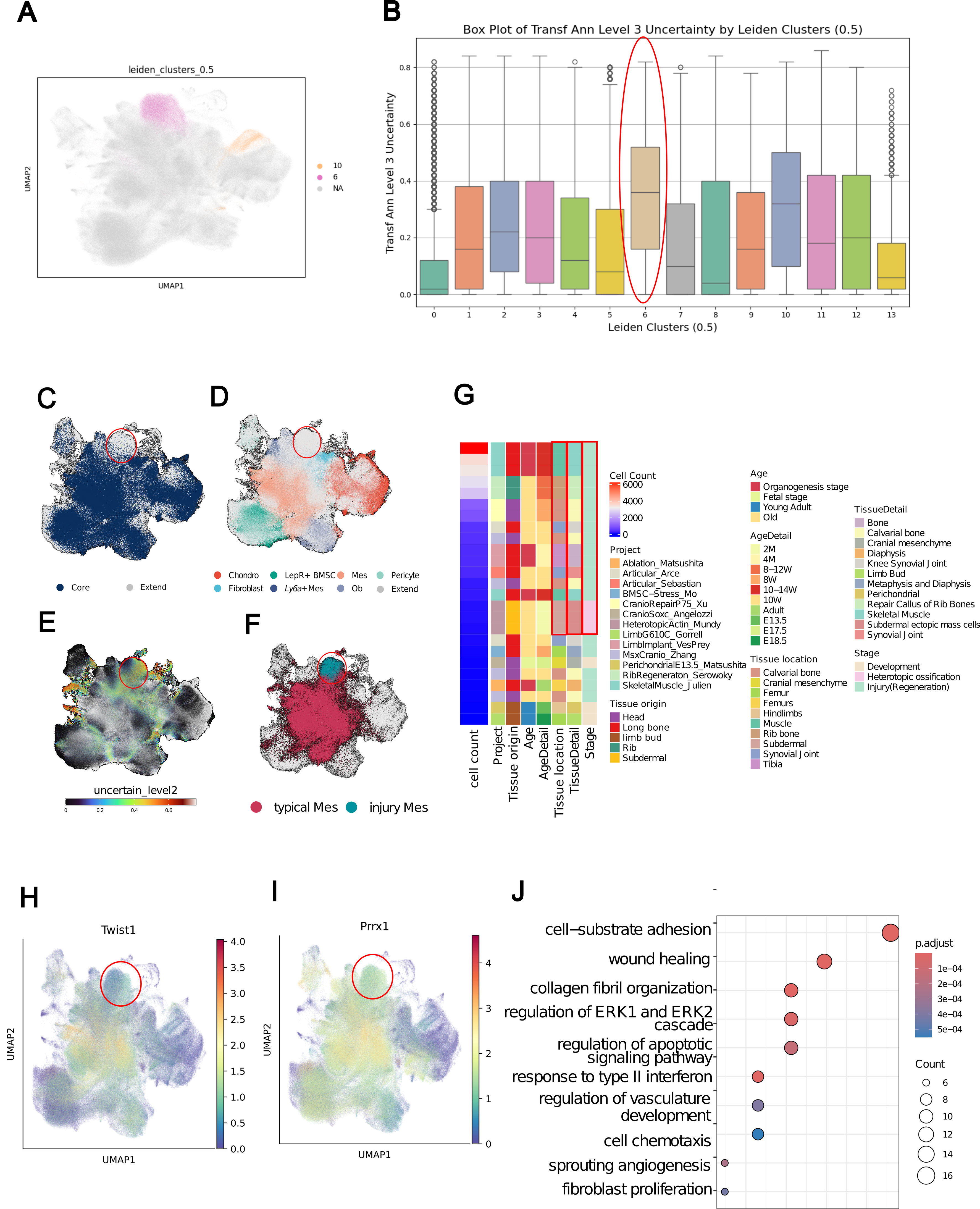

Supplement: S23 Fig — a, UMAP visualization of jointly embedded Differential Atlas (core) and the projected datasets (Extend) b, UMAP visualization of level-2 annotation in Differential Atlas. c, UMAP visualization of uncertainty score (Methods) d, UMAP visualization to highlight typical Mes and injury Mes. g, Heatmap visualization of cell count (fist columns) and metadata of injury Mes in (d). f,h, UMAP visualization of typical Mes marker. i, GO enrichment of differential genes between injury Mes and typical Mes, visualized with dotplot. (TIF) [file pgen.1011319.s023.tif]

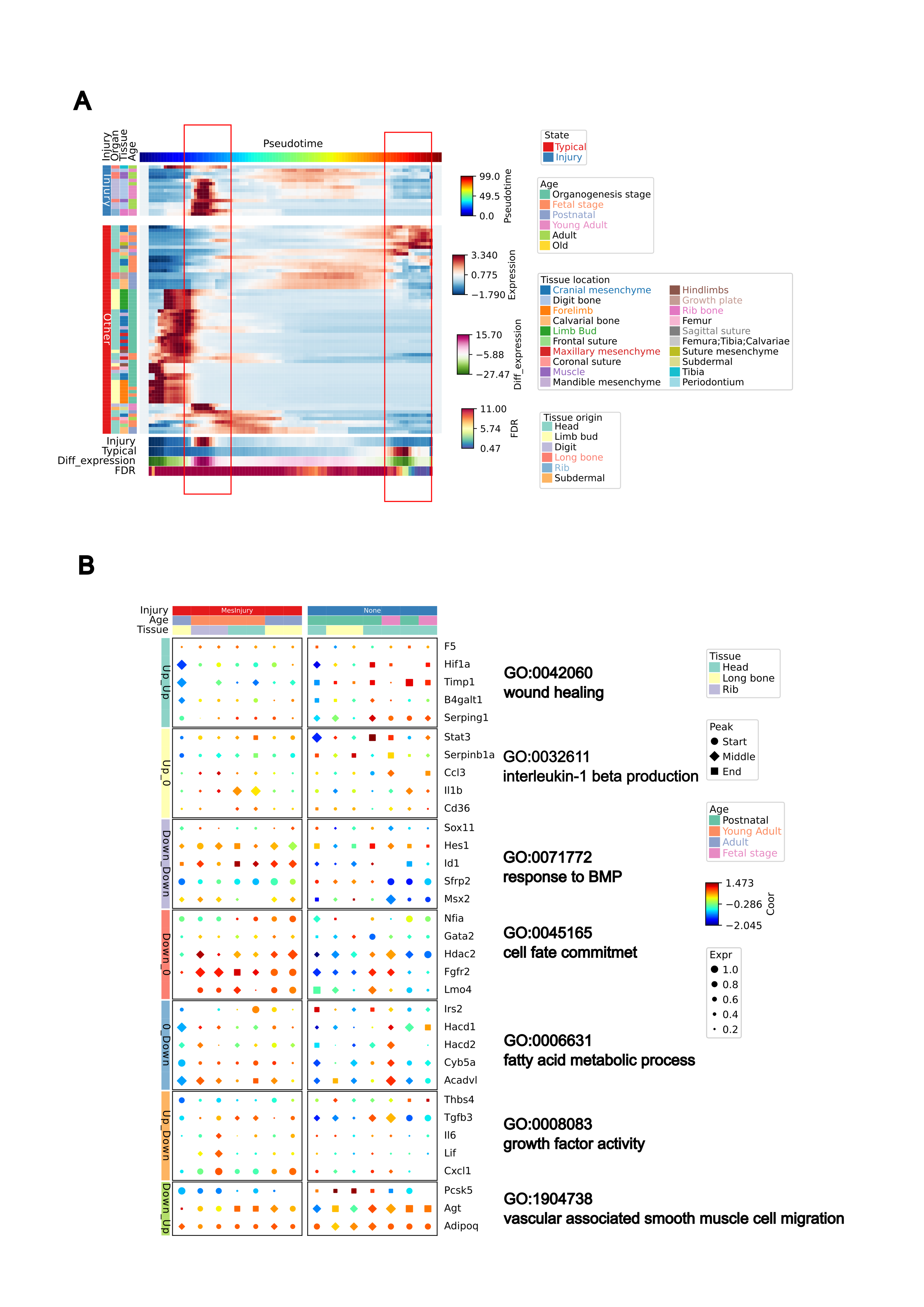

Supplement: S24 Fig — A, Heatmap illustrates difference in cell abundance along pseudotime (column) in Mes OPCST trajectories (row) between injury and typical state. The four rows of bottom annotation represent: Mean cell abundance of the two groups (row 1, row 2), Differential abundance (row 3), and False discovery rate (FDR) (row 4). B, Trajectory dotplot illustrates expression of GO-enriched genes from seven gene cluster in Fig 6F across Mes OPCST trajectories. (TIF) [file pgen.1011319.s024.tif]

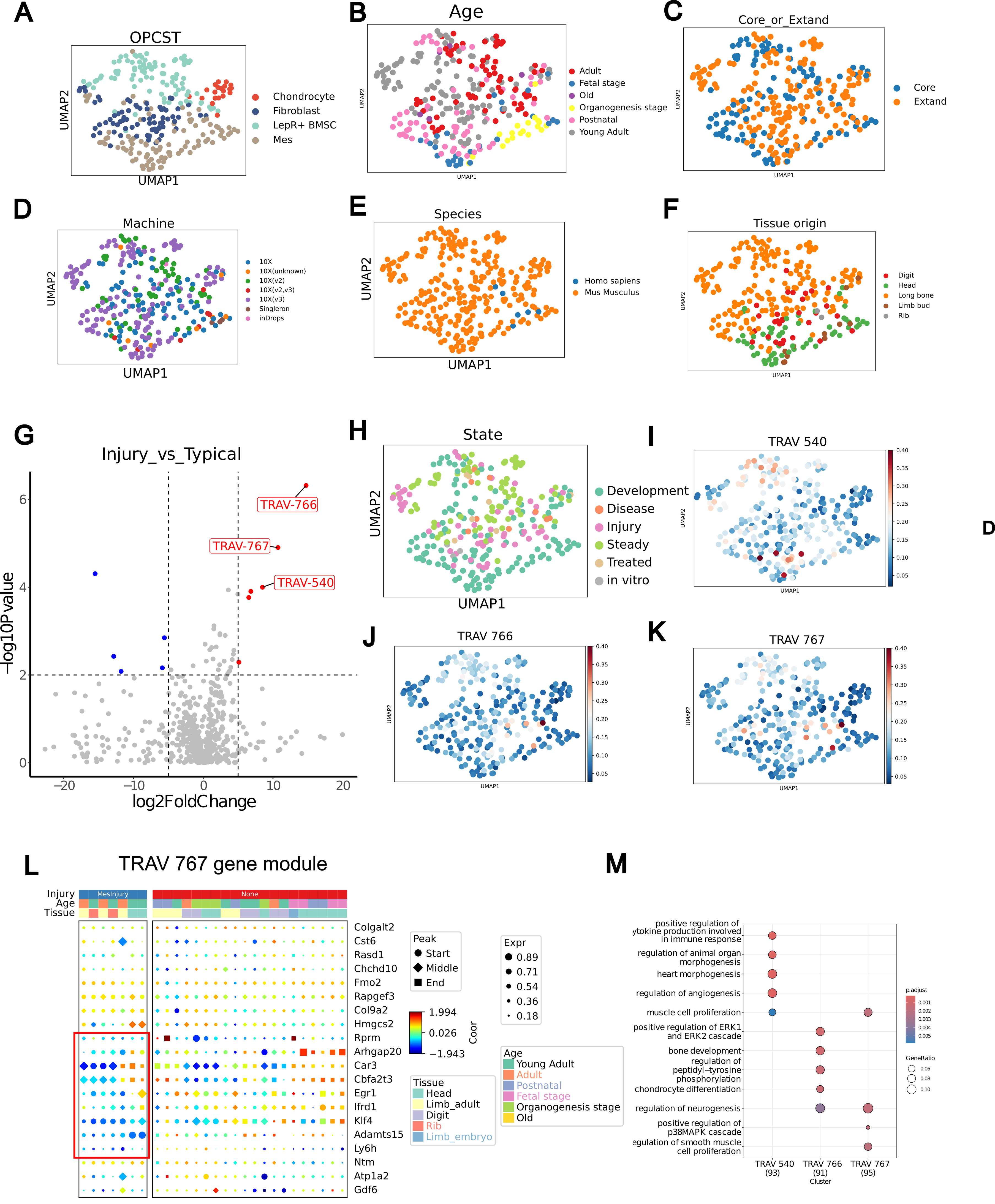

Supplement: S25 Fig — a-f,h, Trajectory embeddings visualized with UMAP, colored by (a) OPCST, (b) Age, (c) deriving from the Differentiation Atlas or projected datasets, (d) Machine group, (e) Species (f) Tissue origin, and (h) State. g, Volcano plot shows injury-related TRAVs. i-f,TRAV activity visualized with trajectory embeddings, colored by injury-related TRAV. l, Trajectory dotplot illustrates expression of genes of TRAV 767 gene module across Mes OPCST. mGO enrichment of three TRAV gene modules, visualized with dotplot. (TIF) [file pgen.1011319.s025.tif]
