# Supplementary material for: Trajectory-centric framework TrajAtlas reveals multi-scale differentiation heterogeneity among cells, genes, and gene modules in osteogenesis
Source: PLoS Genet. 2024 Oct 22;20(10):e1011319. doi: 10.1371/journal.pgen.1011319 (PMC11530032; doi:10.1371/journal.pgen.1011319)
Supplement: S1 Text — (DOCX) [file pgen.1011319.s032.docx]

**Supplementary Notes**

Note 1: Metadata template

**Sample**: Sample name. The format should be "[unique name]_[author]_[sample id]" like "BmscTime_Zhong_16M".

**Project**: Project name. The format should be "[unique name]_[author]_" like "BmscTime_Zhong".

**Tissue origin**: Tissue of sample origin. In the osteogenesis atlas, we divide it based on the mode of ossification into Head (craniofacial region), Limb bud (limb buds during embryonic stage), and Long bone(long bones after birth).

**Tissue location**: Tissue location of sample origin. This is more anatomically oriented, such as specific bone names: frontal bone, tibia, and femur.

**Tissue(Histology)**: Detailed tissue of sample origin. This is more histologically oriented, such as Diaphysis, Metaphysis, Perichondrial, etc.

**Stage**: Divided into developmental, regenerative, steady-state stages, etc.

**Gene type**: WT, KO, MUT, etc. genotypes.

**Treatment**: Any treatments applied, otherwise None.

**Age**: Divided into Organogenesis stage (E8-E14), Fetal stage (E14.5-E18.5), Postnatal (P0-P30), Young Adult (1M-3M), Adult (3M-12M), Old (>12M).

**Age.In.Detail.**: Use E for Embryo, P for Postnatal, W for Week, M for Month.

**Machine**: 10X (divided into v2, v3), SmartSeq2, etc.

**Species**: Mus musculus, Rattus norvegicus, Homo sapiens or other.

**Bone.Forming.Methods**: Conventional modes of bone formation, such as intramembranous ossification and endochondral ossification. If there is controversy, mark it as Unclassified.

**Cell origin**: Cell origin, Neural Crest or Mesoderm. If both, mark it as Mixture.

**Related.Assay**: If the article has been published, fill in the full name of the article.

**Data.Source**: Fill in the GSE accession or Facebase accession, etc.

Note 2: Unified annotation

We roughly use the following gene markers for unified cell annotation across tissues and datasets:

Chondrocyte: *Sox9*, *Acan*.[^[[1]](#endnote-0)^,^[[2]](#endnote-1)^]

LepR+ BMSC: *Cxcl12*, *Lepr* [2]

Fibroblast: *Postn*, *S100a4* [1]

Meninges: *Foxd1*, *Cldn11*, *Lama1* [1]

Derm: Twist2 [1]

Pericytes: *Acta2*, *Rgs5*. [1 2]

Mes: *Prrx1*, *Twist1*. [1]

Osteoblast: *Alpl*, *Sp7*, *Bglap*. [1 2]

Note 3: Endpoint identification

Our Differentiation Model aims to construct a unified trajectory model. However, when dealing with various OPCs, theoretically, there could be millions of trajectories. To simplify this issue, we categorize OPCs using level-2 annotations. Based on Waddington's theory [^[[3]](#endnote-2)^,^[[4]](#endnote-3)^], there exists a single highest potential OPC state from which other OPCs can be derived in each type of OPC. There are several methods to identify the endpoint, or alternatively, the highest potential OPC state. For instance, **CytoTRACE** utilizes transcriptional diversity measurements to predict developmental potential, while **SCENT [**^[[5]](#endnote-4)^**]** relies on entropy to estimate differentiation potency.^[[6]](#endnote-5)^ These approaches help in identifying the endpoint or the most significant state in the trajectory of OPCs. For developing tissues, earlier time points typically possess higher developmental potential. [^[[7]](#endnote-6)^]

Mes: In our atlas, Mes are mostly composed of head and limb bud mesenchyme. We identified the endpoint based on **CytoTRACE** and development time points. In our atlas, Pclaf+ early Mes exhibit the highest **CytoTRACE** potential and are observed at the earliest time points, predominantly around E8-E11 (S8B Fig).

Chondrocytes: There are two models for the transformation of chondrocytes into osteoblasts. In the first model, immature chondrocytes in the growth plate can differentiate into transient osteoprogenitor cells in the metaphysis. In the latter model, hypertrophic chondrocytes redifferentiate into osteoblasts. Here, we will focus only on the latter model, as the former involves an intermediate stage known as the osteogenic precursor. There was evidence that hypertrophy chondrocytes process high innate potential for plasticity [^[[8]](#endnote-7)^], and serve as a reservoir for osteoblasts.[^[[9]](#endnote-8)^] And **CytoTRACE** identified *Ihh*+ hypertrophic chondrocytes as the highest potential cells (S8B Fig). So we identified *Ihh*+ pre-hypertrophic chondrocytes as an endpoint.

Fibroblast: For fibroblasts, we discovered that one subcluster exhibits high expression of adult stem cell markers such as *Ly6a*, *Thy1*, and *Cd34*.(S8A Fig) This cell group overlaps with early mesenchymal progenitors labeled in ref.[^[[10]](#endnote-9)^]. So we identified *Anxa8*+ *Aspn*+ fibroblasts as an endpoint.

LepR+ BMSC: The osteogenic potential of LepR+ BMSCs remains a subject of debate within the scientific community. Different research groups have proposed various interpretations regarding the identity and function of these cells. Some research groups have identified LepR+ BMSCs as OPCs. [2,^[[11]](#endnote-10)^] Other groups have proposed that only a small subset of LepR+ BMSCs possess osteogenic potential, implying heterogeneity within this cell population.^[[12]](#endnote-11)^ ^[[13]](#endnote-12)^Additionally, there are assertions suggesting that LepR+ BMSCs predominantly give rise to adipose lineage cells, implying their role in adipogenesis [10]. In our approach, we adopted the perspective proposed by Yuki et al [^[[14]](#endnote-13)^], whose study in 2020 demonstrated that quiescent Cxcl12-creER+ BMSCs (LepR+ BMSCs in our atlas) can convert their identity into a skeletal stem cell-like state in response to injury. Thus, we identified LepR+ BMSCs as the endpoint based on this evidence.

Note 4: Psuedotime construction

One characteristic of our Differentiation Models is the presence of multiple starting points (Fibroblast, LepR+ BMSC, Mes, Chondrocyte) transitioning to a single ending point(Osteoblast) (Fig 3A). However, existing pseudotime construction tools don't align well with this scenario.[^[[15]](#endnote-14)^,^[[16]](#endnote-15)^] Constructing pseudotime separately for each OPCST renders the final pseudotime from different OPCSTs incomparable. While there are numerous trajectory alignment algorithms, currently [^[[17]](#endnote-16)^,^[[18]](#endnote-17)^], there is no algorithm capable of aligning multiple trajectories and obtaining a unified pseudotime. Conversely, constructing pseudotime in reverse, from end to start, contradicts biological assumptions, and the inferred endpoint may not necessarily align with the designated starting point. Our approach entailed constructing pseudotime for each OPCST independently, followed by employing machine learning methods to generalize the pseudotime.

We aimed for the pseudotime to fulfill the following functions:

• Predict the differentiation process,

• Compare differentiation processes within and between trajectories,

• Serve as an indicator of osteogenic differentiation progress.

We compared the pseudo-time obtained from three different approaches:

1. using **Palantir [**^[[19]](#endnote-18)^**]** starting from osteoblasts

2. using **Palantir** starting from stem cells,

3. using **Scanpy**'s diffusion pseudotime [^[[20]](#endnote-19)^] starting from osteoblasts.

We compared the results of four trajectories individually:

Mes OPCST: When designating osteoblasts as the starting point, Palantir infers an endpoint (i.e., stem cells) with a significant deviation from the theoretically expected stem cell position. The results of approach 2 are closer to the expected outcomes than we anticipated. Diffusion pseudotime exhibits extreme values, deviating significantly from the anticipated expectations (S9A Fig).

LepR+ BMSC OPCST: The results of approch1 and approch2 are similar. In this situation, to ensure comparability of results, we have opted for approach 1 (S9A Fig).

Fibroblast OPCST: Approach 1 yields fewer extreme values and a more evenly distributed outcome (S9A Fig).

Chondrocyte OPCST: Approach 2 yields fewer extreme values and a more evenly distributed outcome (S9A Fig).

After obtaining results from different pseudotime construction methods, we aimed to establish comparability among them. Since there are overlapping trajectories between distinct lineages, it is essential to have a unified standard for assessing the four trajectories. Furthermore, we sought a more universal criterion to measure the process of osteoblast differentiation.

We had considered applying a regression prediction model to adjust pseudotime because we hypothesized that a model with generalization ability can correct errors in pseudotime obtained from different tools. This approach aimed to learn indicators that reflect the differentiation capacity.

We primarily utilized two metrics as criteria for assessment:

Accuracy: We measure the error concerning the labels using Root Mean Square Error (*RSME*) and R-squared (*R^2^*).

Generalization Ability: We performed one-way ANOVA on the pseudotime of different OPCSTs within neighborhoods over the k-nearest neighbor (KNN) graph. The average of the obtained F-values served as the standard for generalization ability (Equation below)(Equation below).

$$\overline{F}=\frac{1}{n}\sum_{i=1}^{n} \frac{{MS}_{between,i}}{{MS}_{within,i}}$$

We conducted performance testing using lazyPredict on 35 regression learning algorithms and a neural network model with seven layers. To visualize these metrics, we employed the **funkyheatmap** (https://github.com/funkyheatmap/funkyheatmap/) and ggplot2 in R. We observed that models with stronger predictive capabilities tend to exhibit weaker generalization abilities. In the end, we used the composite score $\frac{1}{2}R^{2}+ RMSE + \frac{1}{2}\overline{F}$and selected the **LGBMRegressor** with the highest score as our final learning model(S9B,C Fig). We validated its predictive accuracy by dividing the four lineages into 10 bins based on pseudotime and applying similarity algorithms. This process confirmed its outstanding generalization ability (S9D-F Fig).

We validated our model on independent single-cell and bulk RNA datasets, and it demonstrated excellent predictive performance in both cases. For the single-cell RNAseq dataset, we chose sequencing data from the bone marrow of diabetic mice long bones (GSE221936). The preprocessing approach aligned with the previously described method. For bulk RNA data, we selected RNA-seq datasets from pre-osteoblast cells in calvarial cells at different differentiation time points (GSE54461). This indicated that our model has the ability to make accurate predictions for osteoblast differentiation (S9G-I Fig).

Note 5: Trajectory dotplot

There are many tools available for representing the relationship between gene expression and pseudotime.[^[[21]](#endnote-20)^,^[[22]](#endnote-21)^,^[[23]](#endnote-22)^] However, none of these tools can effectively visualize genes in large-scale trajectories. This limitation makes the comparison of genes in large-scale trajectories difficult. Here, we proposed a visualization method that extracts three attributes from the trajectory, enabling the visualization of multiple genes in large-scale trajectories.

We summarized the relationship between gene expression and trajectory into a few characteristic values, such as the Pearson correlation coefficient. These characteristic values can be categorized into three categories. First is the correlation between expression and pseudotime, for example, Pearson correlation coefficient, and mutual information coefficient.

The second category is the expression, for example, area under the curve (AUC), maximal expression. The third category is expression peak, such as pseudotime at maximal expression, pseudotime at minimal expression.

To explore which characteristics retain the most information on the gene expression along the trajectory, we utilized random forest regression for feature selection.

For raw expression, we found that the area under the curve (AUC) is the most important predictor (S14B Fig). For expression fitted by a generalized additive model (GAM), we found that the Pearson correlation coefficient and pseudotime at maximal expression are the most important (S14B Fig). Therefore, we selected AUC, Pearson correlation coefficient, pseudotime at maximal expression as three characteristics. We proved that these three characteristics can well predict raw gene expression (MSE=0.14, S14C Fig) and expression fitted by GAM (MSE=0.01, S14C Fig).

We utilized a dot plot to visualize these three characteristics. The size of the dot represents the area under the curve (expression). The color of the dot represents the Pearson correlation coefficient (correlation). Pseudotime at maximal expression (peak) is represented by the shape of the dot (S14D-I Fig).

We took *Aspn* expression in LepR+ BMSC OPCST from time-series datasets (GSE145477 [10]) for example. Gene Aspn exhibited high expression (large size) at the 1.5M group and increased with pseudotime (high correlation), reaching its highest value at the end stage (peak). In the dot plot, this would be represented by a large-sized dot, colored red for high correlation, and shaped like a square to denote pseudotime at maximal expression (S14G Fig).

Note 6: Benchmarking for tools used in assessing differential abundance (DA) and differential expression (DE)

For DA benchmarking, we evaluated the performance in three ways. In all three approaches, we utilized the LepR+ BMSC datasets from our data, where **TrajDiff**, **Lamian**, and **condiments** software have both detected differential abundance. correlated with pseudotime in both the “Young” and “Adult” groups.

Approach 1 (Sensitivity): We maintained the barcodes of 0%, 10%, 20%, ..., and 100% of cells unchanged, while randomly shuffling the barcodes of the remaining cells. This approach allows us to assess the software's sensitivity to composition differences. The number of times differences are detected within 10 attempts is denoted as *s*.

Approach 2 (Specificity): Cells were randomly divided into 40 samples and 2 groups in proportion, and then the correspondence between samples and groups was randomly shuffled. This process is repeated 10 times to test for false positive rates. The number of times differences are detected within 10 attempts is denoted as *r*.

Approach 3 (Local variations detection): Cells were randomly divided into 40 samples and 2 groups in proportion. The pseudotime axis is then divided into 5 bins, and sequentially, 75% of cells from group 1 were removed in each bin. This method assesses whether the software can accurately detect pseudo-time differences.

For DE benchmarking, we evaluated using the methodology outlined in Lamian [23]. In all three approaches, we utilized the LepR+ BMSC datasets from our datasets. We randomly assigned cells equally into 10 samples, with five samples designated as Group 1 and the remaining five as Group 2.

Approch1 (Sensitivity): We replicated the expression of *Adipoq* to simulate 100 genes. We shuffled the expression of *Fn1* to generate a simulated signal. In Group 2, we added the simulated signal of Fn1 to each gene, with a signal ranging from 0.001 to 0.1. In Group 1, no modifications were made. This method allowed us to assess how sensitive the tool is in detecting subtle signal changes.

Approch2 (Trend): We replicated the expression of *Adipoq* to simulate 100 genes. In Group 2, we added the expression of *Fn1* to each gene, with a signal ranging from 0.001 to 0.1. In Group 1, we shuffled the expression of *Fn1* to generate signals indicating trend differences. In Group 2, we did not shuffle the expression of *Fn1* to generate signals indicating mean differences. This method enabled us to evaluate whether our tools can detect trend differences.

Approch1 (generality): We randomly selected 100 highly variable genes in LepR+ BMSC datasets. We shuffled the expression of *Fn1*, and multiplied 0.0 to generate a simulated signal. In Group 2, we added the simulated signal to each gene. In Group 1, no modifications were made. This method allowed us to assess whether our tools can detect differences in gene expression across any scenario.

We also compared the runtime between different software. Additionally, we generated gene-cell matrices of sizes 100 x 4000, 500 x 10000, and 2000 x 20000. Both **Lamian** and **TrajDiff** were executed with 10 cores using their default parameters.

In the differential abundance benchmarking, we observed that **TrajDiff** exhibited high accuracy and high specificity, while simultaneously being capable of detecting local variation (S15A-I Fig). Although **condiments** achieves high accuracy, it has a high false positive rate when detecting randomly shuffled groups (S15G-I Fig).

In the differential expression benchmarking, we observed that **TrajDiff** had a good performance at detecting mean and trend differences. However, it exhibited lower generality than **Lamian**. This is mainly because **TrajDiff** is designed to capture local variations, so it is not able to detect global changes in gene expression. We noticed that **TrajDiff** was much faster than **Lamian**. As the matrix size increased, the running time did not increase substantially for **TrajDiff**. However, **Lamian**’s running time increased linearly with the matrix size (See also https://github.com/Winnie09/Lamian/issues/17, https://github.com/Winnie09/Lamian/issues/28). This indicates that **TrajDiff** is more suitable than **Lamian** for application to large datasets.

Note 7: Trajectory reduction

To better visualize TRAV activity on large-scale trajectories and unveil relationships between trajectories, we conducted trajectory reduction. We have demonstrated that correlation, peak expression, and amplitude can effectively reconstruct gene expression along pseudotime previously (Notes 6 in S1 Text). Here, we derived three matrices of size *N* × *G* (number of trajectories × number of genes) by computing these trajectory attributes for each gene. Additionally, we generated a TRAV activity matrix with dimensions *N* × *T* (number of trajectories × number of TRAV). After treating each matrix as a modality, we integrated these four modalities using weighted-nearest neighbor (WNN) [^[[24]](#endnote-23)^] by **muon** (https://github.com/scverse/muon) [^[[25]](#endnote-24)^].

After WNN integration, we noticed that trajectories with with same OPCST and project tend to cluster together, confirming that our trajectory reduction can indeed restore the relatedness between trajectories. Trajectory reduction offers a straightforward method for exploring the activity of genes and TRAVs across large-scale trajectories.

### Reference

1. 1 Angelozzi M, Pellegrino da Silva R, Gonzalez MV, Lefebvre V. Single-cell atlas of craniogenesis uncovers SOXC-dependent, highly proliferative, and myofibroblast-like osteodermal progenitors. Cell Rep. 2022;40(2):111045. doi:10.1016/j.celrep.2022.111045 [↑](#endnote-ref-0)
2. 2 Baryawno N, Przybylski D, Kowalczyk MS, et al. A Cellular Taxonomy of the Bone Marrow Stroma in Homeostasis and Leukemia. Cell. 2019;177(7):1915-1932.e16. doi:10.1016/j.cell.2019.04.040 [↑](#endnote-ref-1)
3. 3 Teschendorff AE, Feinberg AP. Statistical mechanics meets single-cell biology. Nat Rev Genet. 2021;22(7):459-476. doi:10.1038/s41576-021-00341-z [↑](#endnote-ref-2)
4. 4 Moris N, Pina C, Arias AM. Transition states and cell fate decisions in epigenetic landscapes. Nat Rev Genet. 2016;17(11):693-703. doi:10.1038/nrg.2016.98 [↑](#endnote-ref-3)
5. 5 Gulati GS, Sikandar SS, Wesche DJ, et al. Single-cell transcriptional diversity is a hallmark of developmental potential. Science (New York, NY). 2020;367(6476):405. doi:10.1126/science.aax0249 [↑](#endnote-ref-4)
6. 6 Teschendorff AE, Enver T. Single-cell entropy for accurate estimation of differentiation potency from a cell’s transcriptome. Nat Commun. 2017;8(1):15599. doi:10.1038/ncomms15599 [↑](#endnote-ref-5)
7. 7 Schiebinger G, Shu J, Tabaka M, et al. Optimal-Transport Analysis of Single-Cell Gene Expression Identifies Developmental Trajectories in Reprogramming. Cell. 2019;176(4):928-943.e22. doi:10.1016/j.cell.2019.01.006 [↑](#endnote-ref-6)
8. 8 Hallett SA, Ono W, Ono N. The hypertrophic chondrocyte: To be or not to be. Histol Histopathol. 2021;36(10):1021-1036. doi:10.14670/HH-18-355 [↑](#endnote-ref-7)
9. 9 Long JT, Leinroth A, Liao Y, et al. Hypertrophic chondrocytes serve as a reservoir for marrow-associated skeletal stem and progenitor cells, osteoblasts, and adipocytes during skeletal development. eLife. 11:e76932. doi:10.7554/eLife.76932 [↑](#endnote-ref-8)
10. 10 Zhong L, Yao L, Tower RJ, et al. Single cell transcriptomics identifies a unique adipose lineage cell population that regulates bone marrow environment. Crump JG, Rosen CJ, Crump JG, eds. eLife. 2020;9:e54695. doi:10.7554/eLife.54695 [↑](#endnote-ref-9)
11. 11 Zhou BO, Yue R, Murphy MM, Peyer J, Morrison SJ. Leptin Receptor-expressing mesenchymal stromal cells represent the main source of bone formed by adult bone marrow. Cell Stem Cell. 2014;15(2):154-168. doi:10.1016/j.stem.2014.06.008 [↑](#endnote-ref-10)
12. 12 Wolock SL, Krishnan I, Tenen DE, et al. Mapping Distinct Bone Marrow Niche Populations and Their Differentiation Paths. Cell Reports. 2019;28(2):302-311.e5. doi:10.1016/j.celrep.2019.06.031 [↑](#endnote-ref-11)
13. 13 Sivaraj KK, Jeong HW, Dharmalingam B, et al. Regional specialization and fate specification of bone stromal cells in skeletal development. Cell Reports. 2021;36(2). doi:10.1016/j.celrep.2021.109352 [↑](#endnote-ref-12)
14. 14 Matsushita Y, Nagata M, Kozloff KM, et al. A Wnt-mediated transformation of the bone marrow stromal cell identity orchestrates skeletal regeneration. Nat Commun. 2020;11(1):332. doi:10.1038/s41467-019-14029-w [↑](#endnote-ref-13)
15. 15 Saelens W, Cannoodt R, Todorov H, Saeys Y. A comparison of single-cell trajectory inference methods. Nat Biotechnol. 2019;37(5):547-554. doi:10.1038/s41587-019-0071-9 [↑](#endnote-ref-14)
16. 16 Ding J, Sharon N, Bar-Joseph Z. Temporal modelling using single-cell transcriptomics. Nat Rev Genet. 2022;23(6):355-368. doi:10.1038/s41576-021-00444-7 [↑](#endnote-ref-15)
17. 17 Sumanaweera D, Suo C, Cujba AM, et al. Gene-level alignment of single cell trajectories. [Preprint]. bioRxiv. 2023. doi:10.1101/2023.03.08.531713 [↑](#endnote-ref-16)
18. 18 Sugihara R, Kato Y, Mori T, Kawahara Y. Alignment of single-cell trajectory trees with CAPITAL. Nat Commun. 2022;13(1):5972. doi:10.1038/s41467-022-33681-3 [↑](#endnote-ref-17)
19. 19 Setty M, Kiseliovas V, Levine J, Gayoso A, Mazutis L, Pe’er D. Characterization of cell fate probabilities in single-cell data with Palantir. Nat Biotechnol. 2019;37(4):451-460. doi:10.1038/s41587-019-0068-4 [↑](#endnote-ref-18)
20. 20 Haghverdi L, Büttner M, Wolf FA, Buettner F, Theis FJ. Diffusion pseudotime robustly reconstructs lineage branching. Nat Methods. 2016;13(10):845-848. doi:10.1038/nmeth.3971 [↑](#endnote-ref-19)
21. 21 Van den Berge K, Roux de Bézieux H, Street K, et al. Trajectory-based differential expression analysis for single-cell sequencing data. Nat Commun. 2020;11(1):1201. doi:10.1038/s41467-020-14766-3 [↑](#endnote-ref-20)
22. 22 Lange M, Bergen V, Klein M, et al. CellRank for directed single-cell fate mapping. Nat Methods. 2022;19(2):159-170. doi:10.1038/s41592-021-01346-6 [↑](#endnote-ref-21)
23. 23 Hou W, Ji Z, Chen Z, Wherry EJ, Hicks SC, Ji H. A statistical framework for differential pseudotime analysis with multiple single-cell RNA-seq samples. Nat Commun. 2023;14(1):7286. doi:10.1038/s41467-023-42841-y [↑](#endnote-ref-22)
24. 24 Hao Y, Hao S, Andersen-Nissen E, et al. Integrated analysis of multimodal single-cell data. Cell. 2021;184(13):3573-3587.e29. doi:10.1016/j.cell.2021.04.048 [↑](#endnote-ref-23)
25. 25 Bredikhin D, Kats I, Stegle O. MUON: multimodal omics analysis framework. Genome Biol. 2022;23(1):1-12. doi:10.1186/s13059-021-02577-8 [↑](#endnote-ref-24)
