# Supplementary material for: Trajectory-centric framework TrajAtlas reveals multi-scale differentiation heterogeneity among cells, genes, and gene modules in osteogenesis
Source: PLoS Genet. 2024 Oct 22;20(10):e1011319. doi: 10.1371/journal.pgen.1011319 (PMC11530032; doi:10.1371/journal.pgen.1011319)
Supplement: Attachment — Submitted filename: PLOS_Genetics_TrajAtlas.pdf [file pgen.1011319.s033.pdf]

Title: Trajectory-centric framework TrajAtlas reveals multi-scale differentiation heterogeneity among cells, genes and gene module in osteogenesis

Authors: Litian Han, Yaoting Ji, Yiqian Yu, Yueqi Ni, Hao Zeng, Xiaoxin Zhang, Huan Liu and Yufeng Zhang

Summary Han et al. built a cell atlas for understanding osteogenesis, by utilising both publicly available and self-generated scRNA-seq data from mouse samples. In terms of methodology, the most prominent novelty of this paper is that they developed a highly modular computational framework for analysing developmental trajectories. This framework enables a 'trajectory-centric analysis', rather than the conventional 'cell-centric analysis'. Another merit worth mentioning is that, in order to harnessing the power of recent method development in data integration, the authors benchmarked many computational methods, before choosing the best-performing ones for building their atlas. All these results will be beneficial for future investigations on cellular developmental trajectories based on atlas-scale data.

However, overall the writing of this paper has hindered the reader's appreciation of its merits. From time to time, I find myself lost in trying to understand the details. I believe this paper contains rich information, but the presentation makes it hard to extract. I also have some concerns about specific analytical steps. Due to the lack of thorough understanding, I will not divide my comments into major and minor ones. Instead I'll simply highlight points that I find hard to understand.

Comments

1. *Introduction (pp. 4–6).* I find the Conclusion section contains some better written review paragraphs, e.g. first paragraph on p. 21 gives a clear overview of the objectives. In contrast, the Introduction section lacks such clearly written paragraphs. The Introduction contains some interesting critiques of existing research on osteogenesis, including crude dichotomy of osteogenesis and failure to address differentiation heterogeneity. However, I find these critiques somewhat hollow:

   (a) It seems that the problem with the existing definition of osteoprogenitor cells (OPCs) is that the existing classification system for them is too crude? And TrajAtlas' contribution is making more refined classification of OPCs? If this

is the case then the problem of existing work is not 'ambiguous definition' but rather overly crude definition.

(b) I suppose the middle paragraph on p. 5 is talking about how existing methods have failed to characterise differentiation heterogeneity of oseteogenesis. However, the description of the limitation of existing methods is overly generalised. It's hard to understand what they are lacking and what TrajAtlas can offer.

2. *Overview of TrajAtlas (p. 7).* As I mentioned, the high modularity of TrajAtlas is a merit that worth emphasising. However, from the Overview it is still not clear what the modules do specifically and how they together form a powerful framework for uncovering differentiation heterogeneity. For example, what are the differences between Differentiation Model and TrajDiff? Why do we need to identify conserved variations in trajectories? These parts lack good explanation on motivation.

3. *OPCs heterogeneity (pp. 8–10).*

   (a) What does 'mapped' mean on p. 8? Usually mapping data A to data B means we treat B as reference and map A as query. This seems not what's being done here? In addition, the authors claimed that on Fig. 2a OPC populations are located 'radially around osteoblasts'. This seems to suggest that osteoblasts are located at the centre on UMAP. However the osteoblasts (I assume they are coloured red on Fig. 2a?) are located at top left corner on UMAP, not at the centre. Even if osteoblasts are located at the centre on UMAP, their location alone cannot suggest the existence of some sort of transition.

   (b) Fig. 2b is not helpful since there are too many colours that are hard to differentiate. If the cell type names/abbreviations can be printed on top of the UMAP it would be much more readable.

   (c) Fig. 2c is very crowded. The orange and green heatmaps don't seem to show very clear patterns as the red one.

   (d) Fig. 2d is easier to interpret. However, there is some significant within-age-group heterogeneity. For example, within fetal limb buds, some samples have a majority of chondro, some samples contain few pericyte; within postnatal long bones, some samples lack LepR+ MSC and Ob populations. Does this heterogeneity reflect some problems regarding sample prepartion or sequencing?

4. *Differentiation Model (pp. 10–13).*

   (a) Fig. 3a was referred to quite frequently in the main text, but clear interpretation was not provided. How should we interpret, e.g., the connectivity? What does higher connectivity means biologically?

   (b) It is not very clear to me what is the main message of Fig. 3f. What's the implication of merged trajectories?

5. *TrajDiff (pp. 13–15).* I find this part relatively easier to understand compared to other parts.

6. *TRAVMap (pp. 15–17).* I fint it hard to get my head around the difference between TrajDiff and TRAVMap. It seems that they are both designed to identify gene modules, and both are related to temporality. In addition, the description of what scPoli do on p. 16 is unclear. How TRAVMap differ from scPoli is also unclear.

7. *Extended atlas (pp. 18–20).* Wht is the source of the 65 datasets being 'projected' onto differentiation atlas? Are they generated by the authors, as described in Methods? Does the word 'projected' here indicate some sort of reference mapping? It seems that the goal of this section is to showcase how we can project external data unto the atlas to help discover the biology in the external data. If so, this point should be made clearer to the reader.

8. *Conclusion (pp. 21–23).* As I mentioned above, the Conclusion is more clearly written than the Introcution. I could follow the flow and picked up key contributions.

9. *Materials and methods (pp. 23–35).*

   (a) In the first round of pre-processing (p. 25), 2000 highly variable genes (HVGs) were selected. Although 2000 is the default number of HVGs in Seurat, it is a rather small number of genes and is by no means the optimal number of HVGs in any given context. Were some of the marker genes pertaining to osteogenesis being left out in this HVG selection step?

   (b) A doublet finder was used in the second round of pre-processing. It seems that doublet identification was also needed in the first round for more accurate clustering?

(c) Batch division (p. 26) doesn't seem to be a common step in scRNA-seq data analysis. What's the motivation to include it here?

(d) I had some major concerns regarding the selection of `n_latent` for training scANVI (p. 27). This parameter is the number of the latent variable used for the variation autoencoder (VAE). Usually this term should be chosen much smaller (e.g. default is 10) than the input data dimensionality, for effective dimension reduction. However, here `n_latent` was selected to be 1500, the same as the input data dimensionality. This might result in serious overfitting.

(e) In OPC mapping (p. 28) it was claimed that 'most EV-OPCs (24/28) were can be mapped to our differentiation atlas'. What was the criterion used for successful mapping?

(f) The binning procedure used in lineage model (p. 30) needs more explanations. It is not very clear how the similarity measurement was calculated.

(g) It was not clear in the main text that the authors generated their own data until p. 34.
