# Supplementary material for: Trajectory-centric framework TrajAtlas reveals multi-scale differentiation heterogeneity among cells, genes, and gene modules in osteogenesis
Source: PLoS Genet. 2024 Oct 22;20(10):e1011319. doi: 10.1371/journal.pgen.1011319 (PMC11530032; doi:10.1371/journal.pgen.1011319)
Supplement: Attachment — Submitted filename: PLOS_Genetics_TrafAtlas_2nd_round.pdf [file pgen.1011319.s035.pdf]

Title: Trajectory-centric framework TrajAtlas reveals multi-scale differentiation heterogeneity among cells, genes and gene module in osteogenesis

Authors: Litian Han, Yaoting Ji, Yiqian Yu, Yueqi Ni, Hao Zeng, Xiaoxin Zhang, Huan Liu and Yufeng Zhang

Summary The resubmitted version of Han et al. is a significantly improved version, with a much clearer abstract, introduction and more explanatory details throughout the main text. Whereas my major concern with the first submission was its unclear presentation, the current version has much improved readability. The authors have also addressed all my technical questions with a lot of additional computational experiments, providing more justification to their modelling strategy.

I have some minor comments regarding the presentation of results.

Minor comments

1. As I mentioned in the previous review, a merit of this paper is its carefully benchmarked computational workflow towards building the atlas. What I did not notice was that this work also included a shiny app and well-documented tutorial. These additional resources were highlighted in the authors' response to reviewers but not sufficiently highlighted in the paper. As a paper on building atlas, the computational methods are also of significant value to the reader. I think it's worth mentioning the computational methodology and additional resources in the introduction. In the current version, the last paragraph in Introduction is on the methodology but it's rather vague.

2. 215–219 on p. 9: the two sentences here are repetitive.

3. Overview of TrajAtlas provided a much clearer description of the four modules of TrajAtlas. I found the short descriptions in bold font easier to understand than the original module names, such as Differentiation Model. It's worth repeating these short descriptions in subsequent sections to remind the reader what each module is about, e.g. at p/ 11 line 279.

4. Line 530 p. 20: I guess the transfer learning method here meant scANVI?

5. Lines 547–562. This paragraph singled out a subset of cells from injury datasets to analyse since they had high uncertainty when projected to the atlas. However, by examining Fig S23C, it seems that this group of cells did not have the highest uncertainty? Some explanations are needed for why choosing this particular subset of cells to analyse.
