# Supplementary material for: Trajectory-centric framework TrajAtlas reveals multi-scale differentiation heterogeneity among cells, genes, and gene modules in osteogenesis
Source: PLoS Genet. 2024 Oct 22;20(10):e1011319. doi: 10.1371/journal.pgen.1011319 (PMC11530032; doi:10.1371/journal.pgen.1011319)
Supplement: Attachment — Submitted filename: 24.7_responses to_the_Reviewers_revised_final.docx [file pgen.1011319.s034.docx]

Dear Editors and Reviewers:

Thank you for your letter and detailed comments concerning our manuscript (PGENETICS-D-24-00584) entitled **“Trajectory-centric framework TrajAtlas reveals multi-scale differentiation heterogeneity among cells, genes, and gene modules in osteogenesis”**. Those comments are all valuable and very helpful for revising and improving our paper, as well as the important guiding significance to our researchers. We have carefully considered the reviewers’ comments and tried to modify the manuscript accordingly.

The point-by-point responses addressing the reviewers’ comments are listed below. Our responses and corresponding changes are highlighted in the text in both this letter and the revised manuscript. The original high-resolution figures referenced in this letter have been deposited under “Revision_Figure” folder in Figshare (https://figshare.com/articles/dataset/Differential_Atlas/25422688).

We are looking forward to receiving your favorable reply.

Yours sincerely,

Yufeng Zhang, Ph.D., DDS

School & Hospital of Stomatology, Wuhan University

237 Luoyu Road, Hongshan District, Wuhan 430079, China

E-mail: zyf@whu.edu.cn

Huan Liu, Ph.D., DDS

School & Hospital of Stomatology, Wuhan University

237 Luoyu Road, Wuhan 430079, China

E-mail: liu.huan@whu.edu.cn

**Responses to the Reviewers’ Comments and the Corresponding Revisions**

## Reviewer #1:

#### Summary

Han et al. built a cell atlas for understanding osteogenesis, by utilising both publicly available and self-generated scRNA-seq data from mouse samples. In terms of methodology, the most prominent novelty of this paper is that they developed a highly modular computational framework for analysing developmental trajectories. This framework enables a ‘trajectory-centric analysis’, rather than the conventional ‘cell-centric analysis’. Another merit worth mentioning is that, in order to harnessing the power of recent method development in data integration, the authors benchmarked many computational methods, before choosing the best-performing ones for building their atlas. All these results will be beneficial for future investigations on cellular developmental trajectories based on atlas-scale data.

However, overall the writing of this paper has hindered the reader’s appreciation of its merits. From time to time, I find myself lost in trying to understand the details. I believe this paper contains rich information, but the presentation makes it hard to extract. I also have some concerns about specific analytical steps. Due to the lack of thorough understanding, I will not divide my comments into major and minor ones. Instead I’ll simply highlight points that I find hard to understand.

Thank you for your encouraging feedback and constructive suggestions. We acknowledge that some aspects of our manuscript lacked clear explanations. To address this, we have thoroughly revised the manuscript, with particular focus on the abstract and introduction sections. We have also created a series of figures in the responses and revised most of the figure in the manuscript, to provide readers and reviewers with a clearer and more intuitive understanding of the content.

Given the complexity of the subject, we found it challenging to cover every detail within the manuscript. Therefore, we have published additional articles on our website [here](https://trajatlas.readthedocs.io/en/stable/introduction/index.html) for readers who wish to explore the topic in greater depth.

Regarding the technical concerns you raised, we have conducted a series of experiments for validation. The code used for these validations is available on [GitHub](https://github.com/GilbertHan1011/TrajAtlasManuscript/tree/main/revision). We hope these revisions address your concerns and enhance the clarity of our work.

#### 1. Introduction (pp. 4–6)

#### 1.a

It seems that the problem with the existing definition of osteoprogenitor cells (OPCs) is that the existing classification system for them is too crude? And TrajAtlas’ contribution is making more refined classification of OPCs? If this is the case then the problem of existing work is not ‘ambiguous definition’ but rather overly crude definition.

Thank you for your detailed critique and valuable insights, which are crucial for refining our manuscript. Indeed, the current definition of OPCs is overly **crude**, and one of our key contributions is the development of a more refined classification system for OPCs. The term ‘**crude’** more accurately reflects the issues with the existing definitions than ‘**ambiguous**’. We have updated the manuscript accordingly. In the following sections, we will address three aspects to discuss the limitations of the current classification system and how TrajAtlas’ approach aims to resolve these issues.

Osteoprogenitor cells are defined with two distinct approaches: scRNA and lineage-tracing^[1]^ (Fig 1), however, their definitions are often too crude.

1) Lineage-tracing-defined classification of OPCs is crude (left panel in Fig 1). The overlap between different lineage-tracing-defined OPCs is unclear. For example, it’s uncertain whether LepR+ BMSCs and *Grem1*+ BMSCs, both discovered in long bones but labeled with different markers, overlap. However, incorporating of these cells into our Differentiation Atlas shows many cells highly express both markers in long bones, suggesting significant overlap between these groups. We have presented these results on our website: <https://zyflab.shinyapps.io/TrajAtlas_shiny/.>
2) Classification defined by scRNA is crude. As illustrated in the right panel of Fig 1, which summarizes annotations from various studies, there is notable variability in how OPCs are defined. For example, Sivaraj et al defined one BMSC population in the long bone as dpMSC based on their spatial expression pattern^[2]^, Baryawno defined a similar population as Lepr-MSC based on cell markers in a unique cell population from the scRNA dataset^[3]^, and Zhong et al defined them as adipocytes based on their potential of adipogenesis^[4]^. However, in our Differentiation Atlas, we find cells with these characteristics are literally the same. Such inconsistent annotations can lead to confusion. By label tranferring with TrajAtlas, we can easily solve this problem, without downloading and integrating with huge datasets. We show this process in the website <https://trajatlas.readthedocs.io/en/stable/tutorial/1_OPCST_projecting.html.>
3) The relationship between scRNA-based OPCs and lineage-tracing-based OPCs is crude (Fig 1). It is crucial to integrate lineage-tracing-based OPCs into single-cell datasets to gain a more comprehensive understanding. However, the limited size of scRNA-seq datasets may obscure certain cellular states. With the Differentiation Atlas, which includes a reference atlas of 270,000 osteogenesis-related cells, we are able to uncover rare cell states that might be hidden in smaller scRNA-seq datasets.

Fig 1 The crudeness of the existing OPCs classification system. The left panel shows OPCs defined through lineage tracing, while the right panel presents OPCs identified via single-cell RNA sequencing (scRNA-seq). We highlight three key issues with the current classification system. By incorporating experimental data and using reference mapping to the Differentiation Atlas, TrajAtlas addresses these issues, providing a more accurate and comprehensive classification system for OPCs.

##### 1.b

I suppose the middle paragraph on p. 5 is talking about how existing methods have failed to characterise differentiation heterogeneity of oseteogenesis. However, the description of the limitation of existing methods is overly generalised. It’s hard to understand what they are lacking and what TrajAtlas can offer.

Thank you for your attentive review and valuable feedback. A thorough literature revision along with the benchmark in our study showed that the current published methods always focused on one or two specific aspects. For example, Slingshot^[5]^ emphasizes the cellular perspective, addressing the order of cells during differentiation and their fates. In contrast, tradeSeq^[6]^ focuses on how gene expression patterns change throughout differentiation (Fig 2). Indeed cell differentiation is a multifaceted process, including the classification of progenitor cells, numerous pathways and transcriptional regulation changes throughout time (Fig 2). The multifaceted process, which is also discussed in detail in **Section** 4.a (Reviewer #2), could be addressed well in our TrajAtlas model, an all-in-one framework for analyzing differentiation. (Fig 2, Fig 27)

We have revised our manuscript to clearly address these issues and highlight how TrajAtlas provides a more integrated approach to understanding differentiation.

*Original version (Fifth paragraph in Introduction):*

In recent years, single-cell technologies have revealed cellular states with unprecedented detail, providing detailed insights that facilitate the reconstruction of differentiation processes^[7]^. Most single-cell atlases^[8]^ ^[9]^ and tools^[10]^ ^[11]^ have focused on cellular state, thereby enabling the identification of cell-type-specific genes, pathways, and gene modules. However, current cell-centric analysis do not fully address the complexities of differentiation heterogeneity. While there are tools designed for trajectory inference and pseudotime analysis, which offer some insights into the temporal aspects of cellular differentiation^[12]^ ^[13]^ ^[14]^, they fall short of providing a comprehensive exploration of cellular dynamics. Specifically, a critical gap exists in the repertoire of available tools. These tools cannot to systematically investigate cells, genes, and gene modules across the diverse spectrum of differentiation trajectories in a dynamic manner. This limitation underscores the need for more sophisticated analytical frameworks that can integrate and interpret the vast and intricate data generated by single-cell technologies, offering a more nuanced and holistic view of cellular differentiation processes.

*Revised version (Fifth paragraph in Introduction):*

In recent years, single-cell technologies have revealed cellular states with unprecedented detail, providing insights that facilitate the reconstruction of differentiation processes. Numerous algorithms have emerged, each aiming to reconstruct differentiation from different perspectives [^[15]^,^[16]^]. However, these methods are often isolated and lack a comprehensive view of differentiation. Each method raises distinct questions—such as the sequence and fate of cells during differentiation (Slingshot [16], Monocles [14]), the changes in gene expression patterns over time (tradeSeq [13]) , and the influence of covariates on this process (Lamian [12], Condiments [^[17]^]). However, to fully understand the dynamics of differentiation, it is essential to examine cells, genes, and gene modules across the diverse spectrum of differentiation trajectories. This limitation underscores the need for more sophisticated analytical frameworks that can integrate and interpret the vast and intricate data generated by single-cell technologies, ultimately providing a more nuanced and holistic understanding of cellular differentiation processes.

.

Fig 2 Existing methods typically focus on one or two specific aspects of differentiation. In contrast, TrajAtlas provides a holistic view, integrating multiple perspectives to offer a more complete understanding of the differentiation process.

##### 2. Overview of TrajAtlas (p. 7).

As I mentioned, the high modularity of TrajAtlas is a merit that worth emphasising. However, from the Overview it is still not clear what the modules do specifically and how they together form a powerful framework for uncovering differentiation heterogeneity. For example, what are the differences between Differentiation Model and TrajDiff? Why do we need to identify conserved variations in trajectories? These parts lack good explanation on motivation.

Thank you for your professional and detailed suggestions.

1) We acknowledge that the overview lacked motivation, which made this section unclear. Therefore, we have reworked this part to make it more question-oriented.

*Previous version (Overview):*

TrajAtlas is composed of four modules, enabling the unraveling of differentiation heterogeneity in a multi-scale manner. In the first module, Differentiation Atlas, we integrated trajectories spanning a wide range of tissues and ages to establish a reference atlas for osteoblast differentiation, encompassing 110 samples and 272,369 cells. We provided a seven-level hierarchical annotation system to reconcile conflicting osteogenesis-related cell types annotated across different studies. Through mapping experimentally validated osteoprogenitor cells to our atlas, we can delve deeper into exploring the heterogeneity of osteoprogenitor cells (Fig 1A). In the second module, we introduced the Differentiation Model, which aims to reconstruct the osteoblast differentiation process. The Differentiation Model leverages four trajectories groups to effectively represent differentiation from various osteoprogenitor cells, offering valuable insights into the key signaling pathways and gene regulatory networks underlying this intricate process (Fig 1B). In the third module, TrajDiff, we presented a tool designed to detect covariate-related differential cell abundance and genes along the differentiation process across multiple trajectories, enabling assessment of the impact of covariates such as age and tissue locations on gene expression during different stages of osteoblast differentiation (Fig 1C). In the fourth module, TRAVMap was introduced, offering innovative methods for identifying conserved variations in trajectories, referred to as Trajectory-related Replicable Axes of Variation (TRAVs). Through TRAVs, trajectory-related gene modules were pinpointed, elucidating their activities within large-scale trajectories (Fig 1D).

*Revised version (Overview):*

Cell differentiation is a complex problem involving changes in cells, genes, and gene modules. To address this complexity in a more comprehensive way, TrajAtlas comprises four distinct modules, enabling the multi-scale exploration of differentiation heterogeneity. (a) **Heterogeneity of Osteoprogenitor Cells**. The first module, the Differentiation Atlas, integrates trajectories across diverse tissues and ages to create a comprehensive reference atlas of osteoblast differentiation, covering 110 samples and 272,369 cells (S1 Table). A seven-level hierarchical annotation system reconciles conflicting osteogenesis-related cell types across different studies. By mapping experimentally validated osteoprogenitor cells onto this atlas, we can more thoroughly investigate their heterogeneity (Fig 1A). (b) **Genetic Regulation and Pathways Driving Osteoprogenitor Differentiation:** the second module, the Differentiation Model, reconstructs the osteoblast differentiation process. It employs four trajectory groups to represent the differentiation pathways from various osteoprogenitor cell populations, offering insights into the critical signaling pathways and gene regulatory networks that govern this process (Fig 1B). (c) **Age and Tissue-Specific Variations in Differentiation**: the third module, TrajDiff, facilitates the detection of covariate-associated differential cell abundance and gene expression along the differentiation process across multiple trajectories. This tool allows for the assessment of factors such as age and tissue-specific location on gene expression patterns during osteoblast differentiation (Fig 1C). (d) **Identification of Gene Modules Related to Differentiation**: the fourth module, TRAVMap, introduces novel methods for identifying robust gene modules across multiple datasets. It enables the investigation of the roles these gene modules play within large-scale differentiation trajectories and their relationship to differentiation processes (Fig 1D). Through these approaches, we enable the unraveling of differentiation heterogeneity in cells, genes, and gene modules.

2) The distinction between Differentiation Model and TrajDiff lies in their focus: the Differentiation Model investigates the intrinsic mechanisms by which osteoprogenitor cells differentiate into osteoblasts, concentrating on the gene regulatory networks and signaling pathways driving this process. In contrast, TrajDiff examines the impact of external covariates—such as age and tissue-specific factors—on the differentiation process, assessing how these variables influence gene expression and cell abundance across various stages of osteoblast differentiation.
3) As for conserved variations in trajectories, genes with similar expression profiles may participate in the same biological processes^[18]^. However, current gene module identification algorithms^[19]^ ^[20]^ often encounter challenges in reliably detecting gene modules across multiple datasets. To address this limitation, we developed TRAVMap. By identifying conserved variations in trajectories, TRAVMap can effectively pinpoints reliable gene modules across different datasets. This method ensures greater reliability in module detection and is discussed in more detail in **Section** 6.g, as noted by Reviewer #2.

#### 3 OPCs heterogeneity (pp. 8–10).

##### 3.(a)

What does ‘mapped’ mean on p. 8? Usually mapping data A to data B means we treat B as reference and map A as query. This seems not what’s being done here? In addition, the authors claimed that on Fig. 2a OPC populations are located ‘radially around osteoblasts’. This seems to suggest that osteoblasts are located at the centre on UMAP. However the osteoblasts (I assume they are coloured red on Fig. 2a?) are located at top left corner on UMAP, not at the centre. Even if osteoblasts are located at the centre on UMAP, their location alone cannot suggest the existence of some sort of transition.

Thank you for your detailed critique and valuable insights, which are crucial for refining our manuscript.

1) The term ‘map’ used on p. 8 refers to the procedure of annotating experimentally validated osteoprogenitors within our atlas. In lineage-tracing experiments, cells expressing marker genes in specific tissues and ages are tracked. Since our atlas integrates tissue, age information, and gene expression profiles, we can annotate lineage-tracing osteoprogenitor cells accordingly. However, the term ‘map’ may indeed be misleading. To clarify, we will use ‘incorporate’ to describe this procedure of integrating lineage-tracing data into our atlas. The detailed incorporating process was discussed in **Section** 9.(e).
2) The sentence ‘radially around osteoblasts’ was not appropriate; in our original context, we wanted to describe the global topology of osteoblast and osteoprogenitors: osteoblasts are at one end of the spectrum, while multiple osteoprogenitors are at the other end. And between osteoprogenitors, there are various intermediate cell states that can be observed in the UMAP. The continuum of cellular states between osteoblast and osteoprogenitors in UMAP can provide visual intuition of cellular transition^[21]^.
3) The locations alone do not definitively indicate the existence of transitions. We do not claim that dimensionality reduction serves as conclusive evidence of cellular transitions between OPCs and osteoblast, as this has already been established by numerous studies^[22]^. However, dimensionality reduction provides a visual representation of how our atlas reconstruct the osteoblast differentiation process. Since reduction can distort global topology^[23]^, we used PAGA connectivity measurements to quantify cellular transitions, which are discussed in **Section** 4.(a). Additionally, we applied various reduction methods, such as force-directed graphs and TriMap^[24]^, which were consistent with those observed in UMAP (Fig 3). We have revised the sentence for clarity and moved it to the first graph in the Differentiation Model section, where it is more appropriately discussed.

*Previous version: (Paragraph 2 in Differentiation Atlas Section)*

Notably, the UMAP and the force-directed graph revealed diverse osteoprogenitor populations arranged radially around osteoblasts, suggesting a potential transition between osteoprogenitor cells and osteoblasts (Fig 2A, Fig 3A).

*Revised version: (Paragraph 1 in Differentiation Model Section)*

In our Differentiation Atlas, we observed a continuum of cellular states between osteoprogenitors and osteoblasts in both UMAP and force-directed graphs, which reconstruct the cellular transitions during osteoblast differentiation.(Fig 2A, Fig 3A).

Fig 3 The continuum of cellular states between OPCs and osteoblast was observed in different reductions.

##### 3.(b)

Fig. 2b is not helpful since there are too many colours that are hard to differentiate. If the cell type names/abbreviations can be printed on top of the UMAP it would be much more readable.

Thank you for your valuable feedback. We have revised the plot accordingly and added the abbreviations at the top of the UMAP.

Fig 4 Revised version of Fig 2B (in manuscript).

##### 3.(c)

Fig. 2c is very crowded. The orange and green heatmaps don’t seem to show very clear patterns as the red one.

Thank you for your careful examination of our figures and your insightful feedback.

1) To reduce the clutter of Fig. 2C, we have removed the fifth level of annotation (Fig 5); the full annotation tree can be found in the related supporting information.
2) The orange and green heatmaps in Fig. 2C (in manuscript), which represent tissue and age information respectively, are used primarily for annotation rather than indicating clear trends. There is a weak correlation between cell type and both age and tissue. While some cell types are specific to certain ages and tissues, others are found across multiple tissues and ages^[25]^. Additionally, even cell states that appear similar can exhibit considerable variability in tissue and age distributions, as documented in other studies^[26]^ ^[27]^. Thus, it is anticipated that the orange (age) and green (tissue) heatmaps may not form distinct patterns.
3) The rich information presented in Fig. 2C (in manuscript), including age and tissue annotations, is crucial for a comprehensive understanding of the “incorporated OPC”. Cells cannot be classified by marker genes alone, as their states are influenced by time and spatial context^[28]^. Inspired by Silvia Domcke et al.'s advocacy for a data-driven, tree-based nomenclature to build a reference cell tree^[29]^, we aimed to describe cell types from multiple perspectives: a) the consensus name of the cell type (leftmost tree), b) corresponding osteoprogenitors (middle brown heatmap), c) the age at which this cell type appears (orange heatmap), d) typical tissue locations (green heatmap), and e) highly expressed genes (rightmost dot plot). This comprehensive approach enables us to establish a robust annotation system.

Fig 5 Revised version of Fig 2C (in manuscript). Cell types are described in multiple perspectives: 1.the consensus name of the cell type (leftmost tree), 2. corresponding osteoprogenitors (middle brown heatmap), 3. the age at which this cell type appears (orange heatmap), 4. typical tissue locations (green heatmap), and 5. highly expressed genes (rightmost dot plot).

##### 3.(d)

Fig. 2d is easier to interpret. However, there is some significant within-age-group heterogeneity. For example, within fetal limb buds, some samples have a majority of chondro, some samples contain few pericyte; within postnatal long bones, some samples lack LepR+ MSC and Ob populations. Does this heterogeneity reflect some problems regarding sample prepartion or sequencing?

Thank you for your thorough inquiry regarding the cell abundance heterogeneity in the differentiation atlas.

1) Yes, sample preparation and sequencing will have a sound influence on cell abundance. One of the most important factor is the isolation approach, particularly in long bones. Taking exclusion of hematopoietic cells and enrich for stromal cells as example, Regev et al. utilized Ter119(low), CD71 (low), CD45-, CD3-, B220-, CD19-, Gr-1-, and Cd11b- to isolate BMSCs^[30]^, while Wolock et al. employed CD45 (low) and Ter119 (low), along with CD31- to isolate BMSCs^[31]^. These differing approaches may influence cell abundance due to varying marker expression across cell types.
2) Besides age and tissue origin in Fig. 2D, as well as sample preparation biases, there are additional covariates that can influence cell abundance. Details on these potential covariates are provided in S1 Table and Note 1 in S1 Text. Key factors include: **a) Tissue Location and Histology**: Different tissue locations within long bones harbor various niches with distinct cellular compositions. For instance, the metaphysis tends to be enriched with *Postn*+ BMSCs, whereas the diaphysis is more abundant in *Lepr*+ BMSCs^[32]^. **b) Age (In Detail)**: Although age is categorized into five time points in Fig. 2D (in manuscript), it remains a continuous variable, and cell abundance can vary significantly within each time point. Research indicates that during young adulthood, osteoprogenitor cells undergo notable transitions in long bones^[33]^.
3) Here, we investigated the impact of these covariates on postnatal long bones and fetal limb buds. We observed variations in cell abundance that may reflect biologically meaningful differences, suggesting that factors such as tissue location are also critical in shaping cellular composition. a) In limb bud samples, we found that the proportion of mesenchymal cells is higher in the forelimbs than in the hindlimbs, with all samples showing a high chondrocyte proportion originating from the hindlimbs (Fig 6A). This difference in cell composition between forelimbs and hindlimbs may reflect the distinct osteogenic processes occurring in the two tissues. However, the limited sample size constrains our ability to draw a definitive conclusion. b) In long bone samples, we also observed that cell proportions are significantly related to tissue location (Fig 6B). Cells in the diaphysis are enriched in LepR+ BMSCs, while chondrocytes are more prevalent in the epiphysis and growth plate. These observations align with previous research findings^33^.
4) It is important to note that in curated reference atlases, cell proportions often vary among datasets due to covariates. We have observed similar phenomena in nearly every large-scale single-cell atlas, such as the human lung atlas^[34]^ and the human endoderm-derived organoids atlas^[35]^. Despite these variations, the large number of samples enables us to derive biologically meaningful conclusions. Furthermore, since our reference atlas focuses on cell states related to osteogenesis, we consider the variation in cell abundance to be acceptable.

Fig 6 Cell abundance heatmap in (A) fetal limb bud and (B) postnatal long bone.

#### 4 Differentiation Model

##### 4.(a)

Fig. 3a was referred to quite frequently in the main text, but clear interpretation was not provided. How should we interpret, e.g., the connectivity? What does higher connectivity means biologically?

Thank you for your thorough inquiry regarding this concept. Connectivity measures continuous cell transitions at the cluster level, where higher connectivity indicates a greater probability of cellular transition. In our context, this means identifying which osteoprogenitors are more likely to directly transition into osteoblasts.

In detail, connectivity was calculated using PAGA^[36]^, a method that utilizes global topology to represent cellular transitions. This method is widely implemented in tools such as scanpy^[37]^, cellrank^[38]^, and scvelo^[39]^ to measure continuous cell transitions at the cluster level. In Fig 3A (in manuscript), nodes represent cell groups and edges represent connectivity. We focus on identifying clusters that directly transition into osteoblasts, as indicated by edges highlighted in red in Fig. 3A (in manuscript). The connectivity measurement helps us quantify the probability of cellular transitions from osteoprogenitors in our model.

We have updated Fig 3A (in manuscript) in the manuscript and revised the sentence in the first paragraph.

*Previous version:*

To achieve this, we employed coarse-grained connectivity structures to capture cell transitions^28^ between osteoprogenitors and osteoblasts (Fig 3A, S7A Fig).

*Revised version:*

To achieve this, we employed coarse-grained connectivity structures to capture cell transitions [^[40]^] between osteoprogenitors and osteoblasts, with higher connectivity indicating a greater probability of transition. (Fig 3A, S7A Fig).

Fig 7 Revised version of Fig.3A (in manuscript).

##### 4.(b)

It is not very clear to me what is the main message of Fig. 3f. What’s the implication of merged trajectories?

We are grateful for your thoughtful feedback and the opportunity to clarify the implications of the merged trajectory.

The main message is to report a merged trajectory model illustrating the diverse trajectories through which osteoprogenitors ultimately converge into a uniform cell type—osteoblasts.

1) It's well-established that various osteoprogenitors can differentiate into osteoblasts. In our OPCST model, we have identified four distinct osteoprogenitor populations that ultimately differentiate into the same cell type, which aligned with previous studies^22 31^. However, the process by which these progenitors converge into a uniform cell type remains unclear. For example, there are at least three distinct ways regarding this convergence (Fig 8). **Hypothesis 1**: All osteoprogenitors pass through common cellular states before ultimately differentiating into osteoblasts. **Hypothesis 2**: Osteoprogenitors transition directly into osteoblasts without a shared intermediate state. **Hypothesis 3**: Some osteoprogenitors converge into a pre-osteoblast state, while others directly become osteoblasts.
2) This problem is essential to understanding osteogenesis, however, due to a lack of algorithms and datasets, this question cannot be fully explored. Most algorithms focus on the lineage decision process, where one progenitor gives rise to multiple cell types. In contrast, our approach can be considered a reverse problem, known as lineage convergence. This concept has been explored in various contexts, including immune cells^[41]^, neural crest lineages^[42]^, and endodermal populations^[43]^. Here, using a new algorithm and large-scale datasets, we are able to build a model based on the similarity of cell states. Our results indicate that the mesenchyme, fibroblast, and LepR+ BMSC lineages converge at the pre-osteoblast stage, whereas the chondrocyte lineage converges at the osteoblast stage (Hypothesis 3) (Fig 8).

Fig 8 Diagram of the three hypotheses. **Hypothesis 1**: All progenitors both go through an immediate pre-osteoblast stage. **Hypothesis 2**: All progenitors both directly become osteoblast without going through a common immediate stage. **Hypothesis 3**: Some progenitors direct become osteoblasts, while others go through pre-osteoblast stage.

To clarify this, we have revised our manuscript to better convey this meaning.

*Previous version (Paragraph 2 in Differentiation Model Section):*

To build the final model, we divided the differentiation path based on osteoprogenitors and pseudotime into bins, then merged bins representing similar cell states (Fig 3F, S10A-H Fig, Methods).

*Revised version (Paragraph 2 in Differentiation Model Section):*

To build the final model, we divided the differentiation path based on osteoprogenitors and pseudotime into bins, then merged bins representing similar cell states (Fig 3F, S10A-H Fig, Methods). The merged trajectory provides an understanding of how diverse osteoprogenitors converge into a uniform cell type—osteoblasts (Fig 3F, S10A-H Fig).

*Previous version of S10 Fig*

Fig 9 Previous version of S10 Fig.

*Revised version of S10 Fig*

Fig 10 Revised version of S10 Fig.

#### 5 TrajDiff

I find this part relatively easier to understand compared to other parts.

##### 6 TRAVMap

TRAVMap (pp. 15–17). I find it hard to get my head around the difference between TrajDiff and TRAVMap. It seems that they are both designed to identify gene modules, and both are related to temporality. In addition, the description of what scPoli do on p. 16 is unclear. How TRAVMap differ from scPoli is also unclear.

Thank you for your detailed scrutiny of our findings and for raising these important questions.

1) **Difference between TrajDiff and TRAVMap.** TrajDiff aims to identify differentially expressed genes between two groups, while TRAVMap focuses on detecting conserved pseudotemporal gene modules across samples. To clarify this distinction, we created an overview (Fig 11). In Fig 11 A and B, we order cells by pseudotime along two axes: the horizontal axis represents pseudotime, while the vertical axis represents samples. TrajDiff detects expression differences between two sample groups along the pseudotime axis, while TRAVMap identifies genes that exhibit common expression patterns across multiple samples. It is worth mentioning that in TrajDiff, while we categorized genes into groups based on expression differences, the biological implications differ from those of gene modules. The “gene group” in TrajDiff refers to genes that exhibit local expression differences between two groups. This concept is similar to that proposed by the tool Lamian^[44]^. In contrast, gene modules in TRAVMap are characterized by genes that display similar expression patterns across the dataset, which was further discussed in **Section** 6.g (Reviewer #2).

Fig 11 Diagrams shows the difference between TrajDiff and TRAVMap.TrajDiff aims to identify differentially expressed genes between two groups, while TRAVMap focuses on detecting conserved pseudotemporal gene modules across samples.

2) **Function of scPoli in p. 16.** scPoli is another tool for capturing sample-to-sample variability, which we used to highlight the distinctive features of TRAVMap. Some algorithms reduce sample dimensions to help explain differences between samples and identify the covariates driving this variation. While tools like scPoli^[45]^ leverage deep learning to analyze sample-to-sample variation, our approach extracts four key attributes from the cell × sample × gene matrix: correlation, peak, expression, and TRAV. We then apply a weighted nearest neighbor (WNN) approach to generate trajectory representations. This method offers greater interpretability compared to deep learning models, as it allows us to visualize these four attributes in a low-dimensional space (Fig 12B-E) —a feature that scPoli cannot provide.

Accordingly, we have revised our manuscript to convey the intended meaning more effectively.

*Previous version:*

Furthermore, by leveraging TRAVs and gene expression data, TRAVMap learned representations of trajectories to capture heterogeneity among samples, similar to a tool called scPoli^[46]^. However, in contrast to scPoli, our approach learns from gene and gene module activities, rendering it more interpretable and enabling a detailed view of gene expression and gene module activity dynamics across population-level trajectories (S19A-E Fig, Note 7 in S1 Text).

*Revised version:*

Furthermore, by leveraging TRAVs and gene expression data, TRAVMap learns representations of trajectories to capture sample-to-sample variability, allowing us to identify the sources of variability. Unlike current deep learning approaches^[47]^, our approach incorporates gene and gene module activities, making it interpretable and providing a detailed view of gene expression dynamics and gene module activity across population-level trajectories (S19A-E Fig, Note 7 in S1 Text).

Fig 12 Characteristics of trajectory reduction. **A**, The diagram illustrates the process of trajectory reduction. **B-E**, Trajectory reductions can reveal TRAV activity, gene expression, gene correlation, and gene peak on the reduction. Gene Expression: Represents the overall expression levels. Gene Correlation: Indicates the correlation between gene expression and pseudotime. Gene Peak: Represents the maximum expression level observed over pseudotime.

#### 7 Extended atlas

What is the source of the 65 datasets being ‘projected’ onto differentiation atlas? Are they generated by the authors, as described in Methods? Does the word ‘projected’ here indicate some sort of reference mapping?It seems that the goal of this section is to showcase how we can project external data unto the atlas to help discover the biology in the external data. If so, this point should be made clearer to the reader.

Thank you for your keen observation and for bringing this discrepancy to our attention.

1) All datasets are derived from publicly available sources, and the data source for each dataset can be found in S1 Table. The reference atlas primarily collects datasets from healthy mice and a limited range of tissues, while the extended atlas includes datasets from disease and injury as well as a wider array of tissues. As you interpreted, the goal of this section is to explore the variation of osteogenesis under various conditions. We apologize for any confusion, but we did not generate any scRNA datasets for this study.
2) The projection includes the following steps: 1. Transfer learning based on a reference model generated with the Differentiation Atlas using scANVI to obtain a latent space; 2. Label transfer; and 3. Trajectory transfer (including lineage transfer and pseudotime prediction). (Fig13) The first two steps are performed using the scANVI framework. The third step is specific to our framework, and we have verified its performance in the S2 Fig.

Accordingly, we revised this part in our manuscript.

*Previous version:*

To investigate osteogenesis within diverse microenvironments, we projected data from 65 datasets encompassing 319 samples and 781397 cells onto the Differentiation Atlas (Fig 6A). This extended atlas incorporated a broader range of tissues (e.g., digit bone, rib, periodontium), encompassed more complex cellular states (injury, heterotopic ossification, disease), and included data from multiple species (human and rat) (S22A-F Fig).

*Revised version:*

Projecting new datasets onto a reference atlas has been shown to effectively detect aberrant cell states [^[48]^]. To investigate osteogenesis within diverse microenvironments, we collected public datasets that incorporated a broader range of tissues (e.g., digit bone, rib, periodontium), encompassed more complex cellular states (such as injury, heterotopic ossification, and disease), and included data from multiple species (human and rat) (S22A-F Fig). We then applied transfer learning to these datasets based on our Differentiation Atlas (Fig 6A). The final dataset comprises 319 samples and 781,397 cells, which we refer to as the extended atlas.

Fig13 The diagram shows the process of projection.

*Origin version of S22 Fig:*

Fig 14 Original version of S22 Fig

*Revised version of S22 Fig*

Fig 15 Revised version of S22 Fig

##### 8 Conclusion

As I mentioned above, the Conclusion is more clearly written than the Introcution. I could follow the flow and picked up key contributions.

##### Thank you for your feedback. We will take this into account and revise the Introduction to ensure it matches the clarity and structure of the Conclusion, making the flow of ideas more cohesive throughout the paper. Your comments will help improve the overall readability and impact of the manuscript.

##### 9 Materials and methods

##### 9.(a)

In the first round of pre-processing (p. 25), 2000 highly variable genes (HVGs) were selected. Although 2000 is the default number of HVGs in Seurat, it is a rather small number of genes and is by no means the optimal number of HVGs in any given context. Were some of the marker genes pertaining to osteogenesis being left out in this HVG selection step?

Thank you for your deep thoughts. We concur that determining the optimal number of Highly Variable Genes (HVGs) is crucial for downstream analysis. Using too few HVGs may exclude important biological variation while too many can introduce technical noise and increase processing time. To investigate whether the arbitrary HVG number affects downstream analysis, we conducted a series of experiments as illustrated below. The results show that most marker genes remain in HVGs, and it won’t significantly affect the downstream clustering.

1) **First, we assessed whether 2000 HVGs preserve biological variance in terms of osteoblast differentiation.** We used the GO term GO:0001649 (osteoblast differentiation), which includes 262 genes, as marker genes. After HVGs selection, we observed that this term was significantly enriched in every dataset’s HVGs (Fig 16 A). We then ranked genes based on standardized variance calculated by Seurat and evaluated cumulative overlap as the number of HVGs increased. Our analysis revealed that, in every dataset, genes associated with the GO term are concentrated among the most highly variable genes. As variance decreased, the slope of the cumulative overlap plot declined, indicating that the biological signal is enriched among the top HVGs. (Fig 16 B,C)
2) **Second, we examined how varying the number of HVGs affects downstream analysis.** We used the level 3 annotation in the reference atlas, which merges a large number of datasets and cells, providing a relatively accurate classification. We varied the number of HVGs from 50 to 8000 and clustered cells using Seurat's default pipeline. We then evaluated cluster reliability using two commonly used metrics: Adjusted Rand Index (ARI) and Normalized Mutual Information (NMI). Our findings indicate that in most datasets, these metrics stabilize when the number of HVGs exceeds 200. (Fig 16 D,E) This suggests that a relatively small number of HVGs is sufficient for achieving reliable clustering results.
3) **The reason we choose 2000 HVGs in the first round of pre-processing**. In the first round of pre-processing, our goal is to filter osteogenesis-related cells based on prior knowledge (clusters) and provide a coarse annotation for integration algorithms (such as scANVI) in the second round. Instead of fine-tuning every parameter as we do in the second round, we use the default Seurat pipeline and create a script to batch process all datasets. We believe this approach will not significantly impact the construction of our reference atlas.

Fig 16 Impact of 2000 HVGs on downstream analysis. **A**. Enrichment of Marker Genes in HVGs: The y-axis represents the -log(p-value) calculated using a hypergeometric test. The results demonstrate that marker genes from each dataset are significantly enriched among the top 2000 HVGs. **B-C**. Cumulative Overlap: These panels show the cumulative overlap between marker genes and HVGs. In each dataset, HVGs are notably enriched among the top HVGs. **D-E.** Evaluation Metrics: The ARI (Adjusted Rand Index) and NMI (Normalized Mutual Information) metrics were used to assess the impact of the number of HVGs on downstream clustering results.

##### 9.(b) doublets finding

A doublet finder was used in the second round of pre-processing. It seems that doublet identification was also needed in the first round for more accurate clustering?

Thank you for your astute observation and for bringing this matter to our attention.

1) **The reason we do not remove droplets in the first round of pre-processing.** As we stated in the previous problem, in the first round, we want to keep as many cells as possible. Because we can always filter it in the second-round process. So, we don’t adapt doublet identification in the first-round process.
2) **Drouplets won’t have a big impact on clustering in our atlas**. Doublets can potentially impact clustering by forming artificial clusters, which may affect our judgment. To test the extent of doublets' impact on clustering, we conducted the following experiments: We selected 66 unprocessed datasets from our atlas and performed the following tests: For each real single-cell dataset, we created three artificial datasets (Fig 17 A):

- Dataset A: Contains both singlets (number = n) and doublets(number = m).
- Dataset B: Contains only singlets (number = n).
- Dataset C: Contains singlets (number = n) and singlets (number = m).

We used the default Seurat pipeline to perform clustering on these datasets. Dataset B served as the critical control, while Dataset C acted as the sham treatment. Datasets A and C had an equal number of cells to ensure that cell count would not bias the downstream analysis. We compared the clustering metrics of A (including doublets) vs B, and C(including singlets) vs B, aiming to evaluate the influence of doublets on clustering performance. We evaluated clustering using the Adjusted Rand Index (ARI) and Normalized Mutual Information (NMI). A paired t-test revealed no significant differences in ARI or NMI between the real treatment (Dataset A) and the sham treatment (Dataset C) (Fig 17 B,C). Additionally, doublets did not form distinct clusters in any of the datasets (Fig 17 D). Therefore, we conclude that the retaining of doublets does not have a significant impact on clustering outcomes in the first-round analysis.

Fig 17 Impact of remaining doublets on the downstream analysis. **A**, Experimental Design: For each dataset, we generated three artificial datasets to evaluate the impact of remaining doublets. **B-C**, Clustering Evaluation: Clustering performance was assessed using ARI (Adjusted Rand Index) and NMI (Normalized Mutual Information) metrics. A paired t-test showed no significant differences in ARI or NMI between the real treatment (cluster A vs cluster B) and the sham treatment (cluster C vs cluster B). **D**, Doublets Clustering: In the datasets (four randomly selected for illustration), we did not observe distinct clusters corresponding to doublets.

##### 9.(c)

Batch division (p. 26) doesn’t seem to be a common step in scRNA-seq data analysis. What’s the motivation to include it here?

Thank you for your thorough inquiry regarding batch division. The motivation behind batch division is to balance the retention of biological variation while minimizing technical variation, thereby improving the accuracy of downstream analyses.

1) Batch correction algorithms have the potential to remove not only technical variation but also biological variation. In our reference atlas, distinguishing cell states with fine resolution is crucial, making it essential to differentiate between technical and biological variation^[49]^. Our atlas includes multi-level batch covariates, such as datasets, samples, age, and tissue location. Deciding which levels of variation to retain and which to correct can significantly impact the results. Although batch division is not a common step in regular single-cell RNA sequencing (scRNA-seq) data analysis, constructing comprehensive atlases often involves this step^[50]^ ^[51]^.
2) Inspired by two excellent papers^51^ ^[52]^, we adopted two methods to determine the appropriate batch covariates:

- Quantifying the Source of Variance: We conducted principal component regression to calculate the fraction of variance explained by the covariate of interest within the first 50 principal components. Our analysis revealed that, in some samples, the variance in cell type was considerably greater than the variance across samples^[53]^ (Fig 18B). Consequently, it is more appropriate to treat the entire dataset as a single batch rather than treating each of the six samples as separate batches. Conversely, in the CranioSoxc_Angelozzi dataset^[54]^, sample variance was even greater than cell type variance. Therefore, it is more appropriate to treat each of the six samples as individual batches in this case.
- Quantifying Batch Effects through kBET: We utilized kBET^52^, which is based on k-nearest neighbors, to assess whether all batches are well-mixed. In the BMSC-Specification_Kishor dataset^53^, we observed that the rejection rates were similar between the observed and expected values (Fig 18E). In contrast, for the CranioSoxc_Angelozzi dataset^54^, the rejection rates were significantly higher than expected (Fig 18 F).

By applying these methods, we meticulously evaluated each dataset to effectively minimize technical variation while preserving biological variation.

Fig 18 Batch division. **A,D**, Overview of two example datasets. **B,E** Source of variance of the two datasets. **C,F,** kBET measurement of these two datasets.

##### 9.(d)

I had some major concerns regarding the selection of n_latent for training scANVI (p. 27). This parameter is the number of the latent variable used for the variation autoencoder (VAE). Usually this term should be chosen much smaller (e.g. default is 10) than the input data dimensionality, for effective dimension reduction. However, here n_latent was selected to be 1500, the same as the input data dimensionality. This might result in serious overfitting.

Thank you for highlighting that important point. This was indeed a typo in our original manuscript. We have benchmarked the combination of variable gene numbers and latent dimensions using SciB pipeline^[55]^ (https://github.com/theislab/scib-pipeline), and it turns out that 15 latent dimensions is optimal. Therefore, we have chosen 15 latent dimensions for the subsequent training. The original code was deposited on GitHub four months ago at [this link](https://github.com/GilbertHan1011/TrajAtlasManuscript/blob/main/other/wt/hyypertune_0-8.ipynb).

Fig 19 The SciB metrics were used to select 1500 HVGs and 15 latent dimensions for final training.

##### 9.(e)

In OPC mapping (p. 28) it was claimed that ‘most EV-OPCs (24/28) were can be mapped to our differentiation atlas’. What was the criterion used for

successful mapping?

**Thank you for your detailed scrutiny of our methods and for raising these important questions. We have established three criteria for our analysis. For an osteoprogenitor (OPC) to be incorporated into a specific cluster, it must: (1) Be present in a specific tissue; (2) be present at a specific age; (3) have the marker gene differentially expressed (adjusted p < 0.01), with over 30% of the cells in the cluster expressing this gene (**Fig 21**).** For example, *Foxa2*+ chondrocytes are identified as long-term osteoprogenitors in both the limb bud and long bone, with specific expression of *Foxa2^[56]^*. In our atlas, we found a cluster named *Ihh*+ Mature Chondrocytes that highly expresses *Foxa2*+ and is present in both the long bone and limb bud. Therefore, we incorporated *Foxa2*+ chondrocytes into this cluster in our reference atlas.

However, due to factors such as the sparsity of single-cell RNA (scRNA) datasets, some genes do not exhibit distinct patterns in our atlas. For instance, although *Gli1* is widely recognized as a stem cell marker across various tissues^[57]^, we do not observe a clear pattern for *Gli1* in our atlas. This limitation is an inherent defect of scRNA technology. When we claim successful incorporation, we acknowledge that this bias is an acceptable constraint within the context of our study.

To make it more rigorous, we have revised our statement.

*Previous version (Methods):*

Most EV-OPCs (24/28) can be mapped to our differentiation atlas.

*Revised version (Methods):*

Most marker genes of EV-OPCs (24 out of 28) are specifically expressed in our atlas, allowing them to be incorporated into our differentiation atlas.

The experimentally validated osteoprogenitor information can be found in S3 Table.

Fig 20 The diagram illustrates the procedures for mapping OPCs.

Fig 21 Examples of successful incorporation and unsuccessful incorporation.

##### 9.(f)

The binning procedure used in lineage model (p. 30) needs more explanations. It is not very clear how the similarity measurement was calculated.

Thank you for your attentive review and valuable feedback on the similarity measurement. We have revised our manuscript to clarify the similarity measurement and conducted an experiment to validate the performance of our algorithm.

1) The similarity algorithm draws inspiration from Augur^[58]^, which prioritizes cell types involved in experimental perturbations. The core idea is that the more similar two cell clusters are, the harder it is to distinguish between them. In brief, the algorithm trains a RandomForest classifier on downsampled cells to predict cluster memberships. It then calculates and normalizes the predicted probabilities for each cell's classification into various clusters, generating misclassification rates that indicate the similarity between clusters based on these probabilities.

*Original version (Methods):*

The similarity measurement was conducted using a random forest model by assessing whether one bin could predict the other. In detail, we generated reference latent representations from the same cells sampled from each bin to train a random forest classifier. Utilizing misclassification rates, we computed a similarity score, reflecting the degree of resemblance between the bins.

*Revised version (Methods):*

The similarity measurement was conducted using a random forest model by assessing whether one bin could predict the other. The core idea of similarity measurement is that the more similar two cell clusters are, the harder it is to distinguish between them. In brief, the algorithm trains a RandomForest classifier on downsampled cells to predict cluster memberships. It then calculates and normalizes the predicted probabilities for each cell's classification into various clusters, generating misclassification rates that indicates the similarity between clusters based on these probabilities.

2) To validate this approach, we used artificial datasets derived from GSE205945^[59]^, focusing on clusters 10 and 12. We shuffled a percentage of the labels (ranging from 0% to 100%) and measured the similarity (Fig 22 A). When x is 0%, we expect low similarity, whereas at 100%, the clusters should appear identical. (Fig 22 b) Our results show a strong correlation between the shuffled percentage and similarity (Spearman correlation coefficient = 0.992) (Fig 22 C), demonstrating that our similarity measurement accurately reflects the true cluster relationships. The Python implementation of the similarity algorithm can be found at this [link](https://github.com/GilbertHan1011/TrajAtlasManuscript/blob/main/0_utils/similarity.py), while the R version is available at this [link](https://github.com/GilbertHan1011/TrajAtlasManuscript/blob/main/0_utils/4.26_similarity_fun.R).

Fig 22 Validation of similarity measurement. **A**,Experimental Design: We select two cell clusters and divide the data into x% unshuffled and 100-x% shuffled portions. **B**, With 0% shuffled data, the two clusters remain distinct in the UMAP plot. In contrast, with 100% shuffled data, the clusters become mixed **C**, the two cluster are mixed. c,The heatmap indicates that the similarity index, as determined by our algorithm, shows a strong correlation with the percentage of shuffled data..

##### 9.(g)

It was not clear in the main text that the authors generated their own data until p. 34.

Thank you for your keen observation and for bringing this discrepancy to our attention. We apologize for any confusion. To clarify, we have not generated our own scRNA data; the description on page 34 pertains to some experimental validation.

To address this point, we have revised the Differentiation Atlas Section and the Methods Section to emphasize that all data is derived from public datasets. The accession numbers for all datasets are available in S1 Table.

## # Review 2

Here, I am providing my expert assessment of the study by Han et al. from the biological perspective in response to the editor’s request. This study presents a novel trajectory-centric package, TrajAtlas, composed of four components of Differentiation Atlas, Differentiation Model, TrajDiff, and TRAVMap, which collectively enable single-cell level deconvolution of highly complex cell lineages such as those for bone-forming osteoblasts. The motivation behind developing a new computation approach is multifold. The osteoblast lineage is unique in that it converges to terminally differentiated cells from diverse cells-of-origins in a manner opposite to the hematopoietic lineage that presents a classical stem cell model with a single master hematopoietic stem cells diverging to numerous terminally differentiated cell types. The diverse origin of osteoblasts and contiguous and indistinctive cell states of the osteoblast lineage necessitates a different approach to model single-cell trajectories. Addressing this issue by focusing on trajectories instead of cell states represents the study's major innovation. My specific comments are listed below for the authors’ consideration.

Thank you for your attentive review and valuable feedback. Guided by a biological question^22^, we employed a range of statistical methods to explore this topic. Your review from a biological perspective has been extremely helpful in revising our manuscript, ensuring we do not get lost in technical details.

To address your concerns, we have reconsidered how our framework can better serve the field of bone research and other relevant areas. We have significantly revised the manuscript to achieve a more rigorous and clear presentation. We hope these revisions address your concerns effectively.

##### Major point

##### 1

The approach successfully models trajectories instead of cell states, providing novel insights into a highly complex converging cell lineage such as osteoblasts. However, the major weakness of the approach is the lack of consideration of spatial information. In matrix-bound cell types such as osteoblasts, cell states, and trajectories are substantially influenced by their locations within the microenvironment. The location of each cell is perhaps more important than the cell state in rendering cell fate decisions. Additionally, the bias is inevitably introduced during cell dissociation and isolation to generate scRNA-seq datasets. The authors should critically appraise the limitation of the current study, particularly regarding the lack of consideration of the spatial information, and discuss the future venue.

Thank you for your detailed critique and valuable insights, which are crucial for refining our manuscript. We think the major limitation falls in the following two aspects. 1. Lack of spatial information. 2. Fail to draw multidirectional cellular transition between cell types.

1) **Lack of spatial information.** In TrajAtlas, we meticulously recorded three levels of tissue information for all collected samples: tissue origin (such as head, limb bud, or long bone), tissue location (for example, frontal bone, tibia), and tissue histology (including diaphysis, stroma) (Fig 23A-D). This enables us to explore cell abundance and expression differences. However, some tissues may lack sufficient samples to derive statistically robust conclusions. Besides, though we collect tissue information, it lacks finer spatial resolution. This finer resolution is crucial for understanding differentiation within specific niches and for elucidating processes such as cell communication^[60]^ and angiogenesis^[61]^ that are related to differentiation. However, with the advancements in spatial omics, constructing a more detailed spatial atlas^[62]^ in the near future seems promising. We have found that TrajAtlas can effectively infer osteogenesis activity across spatial omics datasets (Fig 24). This provides evidence that TrajAtlas holds promise for detecting expression heterogeneity across spatial samples. We plan to release a newer version of TrajAtlas soon, specifically designed to work with spatial omics datasets.
2) **Fail to draw multidirectional cellular transition between cell types.** Our atlas primarily focuses on the transition of different cell states into osteoblasts. However, this transition is likely not unidirectional^[63]^. The multidirectional transitions among various osteoprogenitors are also critical aspects of osteogenesis. Given that single-cell datasets alone make it challenging to recover these multidirectional cellular transitions, incorporating sequencing-based lineage-tracing methods^[64]^ (such as CellTag) holds promise for developing more complex models that can reveal intricate differentiation dynamics soon.

To address the limitation of TrajAtlas, we have revised our manuscript to add one paragraph at the end of the discussion.

*Revised version (Discussion Section):*

While TrajAtlas offers a robust framework for modeling differentiation across large-scale datasets, there are still limitations to address. First, although our reference atlas includes datasets from a variety of tissues, it lacks finer spatial resolution. This finer resolution is crucial for understanding differentiation within specific niches and for elucidating processes such as cell communication [^[65]^] and angiogenesis [^[66]^] that are related to differentiation. However, with the advancements in spatial omics, constructing a more detailed spatial atlas [^[67]^] soon seems promising. Second, our atlas primarily focuses on the transition of different cell states into osteoblasts. However, this transition is likely not unidirectional [64]. For example, bone marrow stromal cells (BMSCs) can differentiate into chondrocytes [^[68]^], and chondrocytes can also differentiate to BMSCs [^[69]^]. The multidirectional transitions among various osteoprogenitors are also critical aspects of osteogenesis. Given that single-cell datasets alone make it challenging to recover these multidirectional cellular transitions, incorporating sequencing-based lineage-tracing methods [^[70]^] holds promise for developing more complex models that can reveal intricate differentiation dynamics soon.

Fig 23 Three levels of tissue information were recorded in TrajAtlas.

Fig 24 Apply TrajAtlas to the spatial mouse organogenesis spatiotemporal transcriptomic atlas (MOSTA) to identify the locations of osteogenesis activity in mice. The pseudotime TrajAtlas predicted aligned with marker genes (Runx2, Sp7, Bglap) expession.

##### 2

The authors state that they mapped experimentally validated osteoprogenitor cells onto their datasets. However, the authors did not clarify the levels of scientific rigor in mapping each cell type. For example, biological evidence from lineage tracing studies should not be treated as equal to that from immunostaining or in vitro culture of cells isolated using a set of cell markers. It is evident that in vivo lineage tracing studies present only reliable biological evidence regarding trajectories. All other approaches would introduce a substantial bias in introducing the outcome. The authors should emphasize the outcomes of in vivo lineage tracing studies as the most rigorous biological evidence and reframe the rationale behind the study as appropriate.

Thank you for your attentive review and valuable feedback. We understand biological evidence from different studies have different levels of scientific rigor. We have summarized the scientific evidence from the 28 studies we collected (Fig 25) and of course, the highest standard of scientific rigor is lineage tracing, with 23 out of 28 osteoprogenitors falling into this category. For example, Worthley et al. used Grem1-creERT2 mice to identify *Grem*+ BMSCs as osteoprogenitors^[71]^. The moderate scientific rigor is sorting and transplantation. For instance, Ambrosi used FACS to identify osteochondral skeletal stem cells (ocSSCs) and perivascular skeletal stem cells (pvSSCs)^[72]^; 3 out of 28 are in this category. The lowest scientific rigor is immunostaining. For example, Rui-Cong et al. identified Cd168+ skeletal stem/progenitor cells^[73]^. 1 out of 28 fitting this classification. After annotating the confidence levels, we observed that most cells in our atlas still exhibit a high confidence level compared to the moderate and low confidence levels. We have included this information in S3 Table (Fig 25). The related manuscript was revised as below:

*Previous version:*

To assess the osteogenic potential of cells within our atlas, we mapped experimentally validated osteoprogenitors based on previously described marker genes and tissue locations (Fig 2A).

*Revised version:*

We curated osteoprogenitor marker genes, tissue locations, ages, and validation methods from 28 studies on osteoprogenitors, the majority of which validate lineage tracing in vivo, providing the highest level of biological evidence (23 out of 28) (S3 Table). We then incorporated these experimentally validated osteoprogenitors based on the previously described marker genes and tissue locations (Methods).

Fig 25 Screenshot of S3 Table: We have added three new columns to this table: 1. **Validate Methods**: Specifies the methods used to validate the OPCs.2. **Driver**: Indicates the driver used if the OPCs are validated by lineage tracing. 3. **Confidence**: Reflects the level of scientific rigor associated with the validation.

Fig 26 Cell distribution by scientific rigor level in Our Atlas. Most cells have high scientific rigor.

##### 3

The abstract needs a significant rework to explain the motivation behind developing a new approach and emphasize its novelty.

Thank you for your insightful comments and suggestions for improving our manuscript. In the revised version, we have highlighted the key motivations behind our framework and emphasized the novelty of each module within it.

*Previous version (Abstract):*

Osteoblast differentiation is crucial for bone formation and maintaining skeletal integrity. Although it is now understood that this process exhibits significant heterogeneity across developmental stages and tissue microenvironments, the underlying mechanisms remain largely unexplored. In the present study, we introduce TrajAtlas, a comprehensive framework that addresses this gap in knowledge. TrajAtlas comprises four modules: a reference atlas (Differentiation Atlas), a differentiation model (Differentiation Model), a tool for differential pseudotime analysis (TrajDiff), and a method for pseudotemporal gene module detection (TRAVMap). By leveraging single-cell technologies, TrajAtlas offers a systematic approach to exploring the multi-scale heterogeneity among cells, genes, and gene modules within population-level trajectories across diverse tissues and age groups. We systematically investigate the impact of age and injury on osteogenesis, providing new insights into osteoporosis and bone regeneration. In conclusion, our comprehensive framework offers novel insights into osteogenesis and provides a valuable resource for understanding the complexities of bone formation.

*Revised version (Abstract):*

Osteoblasts are the cells responsible for bone formation, and they can originate from various types of progenitor cells. However, it is not well understood how these different progenitor cells become osteoblasts, or how factors such as age and tissue location influence this process. This knowledge gap is largely due to the lack of comprehensive tools that can map and analyze the differentiation process. In this study, we introduce TrajAtlas, a novel comprehensive framework designed to fill this gap. TrajAtlas allows us to create a detailed map of osteoblast differentiation, track changes in gene activity, and examine the impact of age and tissue location on this process. Using TrajAtlas, we also identified patterns in gene expression that are consistent across different conditions, helping us uncover new cell states involved in bone healing. This framework not only enhances our understanding of osteoblast differentiation but also offers a versatile tool that could be applied to study how other types of cells differentiate in different contexts.

#### Individual points

##### 4. Abstract:

###### 4.a

It is not clear what the authors mean by saying "heterogeneity of the osteoblast differentiation process." To my understanding, it is not the heterogeneity of the process per se but the diverse cells-of-origins feeding into osteoblasts through different routes that necessitate a new computational approach.

Thank you for your kindness and detailed suggestions.

1) We acknowledge that the diversity of cell origins is a key focus of bone biology and the driving motivation behind this project. To emphasize this point, we have revised our abstract. The full revised version of the abstract is presented in **Section** 3 .

*Previous version (Abstract):*

Although it is now understood that this process exhibits significant heterogeneity across developmental stages and tissue microenvironments, the underlying mechanisms remain largely unexplored.

*Revised version (Abstract):*

However, it is not well understood how these different progenitor cells become osteoblasts, or how factors such as age and tissue location influence this process.

2) In the original context, "heterogeneity of the osteoblast differentiation process," we aimed to emphasize that this heterogeneity arises from multiple aspects. While osteoprogenitors are important, they do not account for all sources of heterogeneity. For example, various covariates, such as age and tissue location, can significantly influence differentiation^[74]^ ^[75]^. To address this, we have developed a multi-scale framework to dissect heterogeneity from different perspectives (Fig 27): a) **What** the heterogeneity entails (e.g., genes, pathways, gene modules); b) **When** the heterogeneity occurs (e.g., early, middle, or late stages); c) **Why** the heterogeneity exists (e.g., contributions from osteoprogenitors, as well as covariates such as age and tissue locations). To clarify this concept, we have revised the relevant paragraph in the Introduction.

*Previous version (Introduction):*

During cell differentiation, trajectories with distinct gene expression and transcription factor activities emerged, leading to variations in the final cell phenotype^[76]^. We refer to this phenomenon as differentiation heterogeneity^[77]^. Several factors contribute to this heterogeneity, including age^[78]^ ^[79]^, tissue location^78 79^, and injury^7^. In this context, studies have shown that the expression of certain genes, such as *Maf*, declines with age^16^. Conversely, under injury conditions in long bones, the Wnt signaling pathway is upregulated to promote bone regeneration^7^. Differentiation heterogeneity is intricately linked to age-associated bone disorders, such as osteoporosis^[80]^, highlighting its significance in the field of bone health. A systematic exploration of this differentiation heterogeneity holds the promise of uncovering new drug targets and propelling forward the fields of drug development^[81]^ and biomedical engineering^[82]^, offering innovative solutions to bone-related ailments.

*Revised version (Introduction) :*

While cell origin may influence the differentiation process, there are many other covariates to consider, such as age [^[83]^,^[84]^], tissue [6], and injury [7], which may also contribute to heterogeneity. For example, studies have shown that the expression of certain genes, such as Maf, declines with age [^[85]^]. Conversely, under injury conditions in long bones, the Wnt signaling pathway is upregulated to promote bone regeneration [6]. The influence can occur in different aspects, including gene expression [^[86]^], transcription factors [^[87]^], and pathways [87], and these changes may vary throughout the differentiation process. For example, the early stages may differ significantly from the later stages [^[88]^]. These factors render differentiation a complex puzzle and make it difficult to fully grasp the concept.

Fig 27 Differentiation can be viewed from various perspectives. TrajAtlas is designed with four distinct modules to address these different aspects comprehensively.

###### 4.b

"Osteoporosis and bone regeneration" is somewhat an overstatement, as they did not directly test these models. It should be changed to "osteoblast differentiation."

Thank you for your thoughtful comments and advice towards improving our manuscript. We have made the corresponding modifications.

*Previous version:*

We systematically investigate the impact of age and injury on osteogenesis, providing new insights into osteoporosis and bone regeneration.

*Revised version:*

This framework not only enhances our understanding of osteoblast differentiation but also offers a versatile tool that could be applied to study how other types of cells differentiate in different contexts.

##### 5.Introduction

###### 5.a

The question needs to be better laid out at the end of the first paragraph. It is not the potential overlap of osteoprogenitors that hinders our understanding of osteoblast differentiation. It is the diversity of cells-of-origins of osteoblasts that necessitates the development of a new approach. Although relatively well-performed, the authors' new approach could only address one aspect without addressing more important spatial aspects.

Thank you for pointing this out. We have made corresponding revisions.

*Previous version (First paragraph in Introduction Section):*

There has been a long-standing debate about how much these osteoprogenitors overlap and how they differently contribute to osteogenesis^5,6^.

*Revised version (First paragraph in Introduction Section):*

The diversity of osteoprogenitor cells necessitates a systematic classification. However, the current approach based on immunophenotypic markers may be too crude to fully capture the big picture of osteoprogenitors [5,6].

###### 5.b

It is unclear what the authors mean when they say, "overlooks the true cellular origins of osteoblasts." There is no single cellular origin for osteoblasts, as demonstrated by lineage tracing studies.

Thank you for your professional suggestions.

The original phrasing is not appropriate. We do not wish to emphasize a single cellular origin for osteoblasts. Instead, we want to highlight that the conventional classification of osteogenesis predominantly relies on histological methods, which tend to oversimplify the diverse sources of osteoblasts. The distinction between endochondral and intramembranous ossification is based on the presence of a cartilaginous template. However, many instances of ossification do not conform to these typical pathways. For example, Seoyeon et al. found that in sutures, there is a distinct form of endochondral ossification that occurs without the typical hematopoietic marrow formation^[89]^. Additionally, many tissues may combine both endochondral and intramembranous ossification during osteogenesis, such as the periosteum^[90]^ and limb buds^22^. Furthermore, advancements in technologies like lineage tracing and single-cell RNA sequencing (scRNA-seq) reveal the diverse origins of osteoblasts, an aspect that cannot be captured by the histological paradigm. All these anomalies challenge the current paradigm, highlighting the need for a cellular-level paradigm to incorporate these advancements and account for the varied origins of osteoblasts. This motivates our development of a new classification system based on cellular state. We have revised the original context to better convey this point.

*Previous version (Second paragraph in Introduction Section):*

The current classification system, which dichotomizes osteogenesis into endochondral and intramembranous based on the presence of a cartilaginous template^[91]^, overlooks the true cellular origins of osteoblasts.

*Revised version (Second paragraph in Introduction Section):*

The current classification system, which dichotomizes osteogenesis into endochondral and intramembranous based on the presence of a cartilaginous template^[92]^, overlooks the diverse cellular origins of osteoblasts.

##### 6. Result

##### 6.a

Can the authors elaborate on this statement? "Diverse osteoprogenitor populations arranged radially around osteoblasts, suggesting a potential transition between osteoprogenitor cells and osteoblasts."

Thank you for your astute observation and for bringing this matter to our attention. A similar concern was raised by Reviewer #1. We want to emphasize the continuum of cellular states between osteoblasts and osteoprogenitors in the UMAP, which provides a visual intuition of cellular transitions for the reader. We have discussed this issue in detail in **Section** 3.(a**)** (Reviewer #1) and made corresponding revisions for a more rigor expression.

*Previous version: (Paragraph 2 in Differentiation Atlas Section)*

Notably, the UMAP and the force-directed graph revealed diverse osteoprogenitor populations arranged radially around osteoblasts, suggesting a potential transition between osteoprogenitor cells and osteoblasts (Fig 2A, Fig 3A).

*Revised version: (Paragraph 1 in Differentiation Model Section)*

In our Differentiation Atlas, a continuum of cellular states between osteoprogenitors and osteoblasts in both UMAP and force-directed graphs was observed, which reconstruct the cellular transitions during osteoblast differentiation (Fig 2A, Fig 3A).

##### 6.b

Figure 2c: Does this amalgamated dataset include periosteal cells? If not, this would represent a significant limitation of the current study. The authors should clarify.

Thank you for your attentive review and valuable feedback concerning the periosteal cells in our atlas. Yes, at least two datasets in the extended atlas include periosteal cells. The first dataset comes from Debnath et al.^[93]^, who sorted CTSK-mGFP positive cells from femurs of P6 mice. The second dataset is derived from Mo et al.^[94]^, who isolated periosteal cells and defined a *Ly6a*+ periosteal subcluster.

Indeed, in our analysis of periosteal cell composition using the first dataset, we observed significant heterogeneity among these cells. Our findings reveal that periosteal cells are predominantly composed of chondrocyte progenitor cells (*Acan*+, *Col2a*+), fibroblasts (*Postn*+, *S100a4*+), and *Ly6a*+ Mes (*Ly6a*+) according to our annotation system. (Fig 28B,C) These results are consistent with Mo et al.’s identification of a *Ly6a*+ periosteal subcluster and align with Duchamp et al.’s finding of a Postn+ periosteal subcluster categorized as osteoprogenitors ^94^ ^[95]^.

Fig 28 Periosteal cells in Differentiation Atlas. **A-B**, UMAP shows distribution of periosteal cells in our atlas. **C**, Cell type abundance of periosteal cells. **D**, Marker genes’ expression in periosteal cells.

##### 6.c

RE: osteogenesis in the resting zone. Osteogenesis does not occur in the resting zone. It occurs adjacent to the hypertrophic zone.

We would like to express our gratitude for your meticulous observation and for bringing this detail to our attention. We carefully re-examined the literature and made corresponding revisions.

*Original version (Third paragraph in Differentiation Atlas Section):*

Chondrocyte progenitor cells (CPCs) typically express *Grem1^6^* and *Pthlh*^[96]^, which are related to osteogenesis in the resting zone of the growth plate^5 [#^(Fig 2C, S5B Fig).

*Revised version (Third paragraph in Differentiation Atlas Section):*

Chondrocyte progenitor cells (CPCs) typically express *Grem1* [6] and *Pthlh* [^[97]^], which may located in the resting zone of the growth plate [97] (Fig 2C, S5B Fig)

###### 6.d

The authors need to treat potential markers for MSCs with more caution. For example, MSCs should not comprise the vast majority of the cell population – if it does, they should no longer be called "stem cells." The authors would need a different and more accurate nomenclature. There is no consensus that Msx2 or Ly6a can be globally used as an adult stem cell marker.

We appreciate your insightful recommendations for improving the quality of our manuscript. We have implemented a rigorous nomenclature change, replacing 'MSC' with 'mesenchymal cells' according to the following rationale, and have re-examined the markers used.

1. **Nomenclature**: The nomenclature "mesenchymal stem cells (MSCs)" is not appropriate here, as MSC is typically associated with adult stem cells found in umbilical cord tissue, endometrial polyps, and menstrual blood^[98]^ ^[99]^. In our atlas, however, "MSC" refers specifically to undifferentiated mesenchymal cells that are present only in the embryonic head mesenchyme and limb bud. Currently, there is no global consensus on the nomenclature of this group of cells. Some researchers designate them based on their multipotent abilities, referring to them as “osteodermal progenitors,”^[100]^ while others use terms based on cellular states, such as "mesenchymal cells,"^[101]^ or focus on tissue locations, like "limb bud mesenchyme."^[102]^ We have changed the term "MSC" to “mesenchymal cells (Mes)” to emphasize the cellular state.
2. **Composition of Cell Population**: The concern that “MSCs should not comprise the vast majority of the cell population” is primarily a nomenclature issue. It is widely observed that undifferentiated mesenchymal cells constitute a significant proportion of the cell population in both the limb bud^101^ ^[103]^ and head mesenchyme^100^ ^[104]^.
3. **Markers of mesenchymal cells**: This issue also largely stems from nomenclature confusion. *Prrx1* and *Twist1* are widely recognized as markers for mesenchymal cells^100^ ^[105]^. And *Msx2* we used is to define a cellular state rather than a stem cell marker^[106]^. However, the expression of “adult stem cell marker *Ly6a*” in the original text is inappropriate, as *Ly6a* is generally considered to be associated with progenitor-like cells^[107]^ ^[108]^ and should not be treated as a universal marker for adult stem cells.

Accordingly, we revised our manuscript as below:

*Original version (Fourth paragraph in Differentiation Atlas Section):*

Besides, we found an MSC-like cell state that highly expressed adult stem cell maker *Ly6a* (stem cell antigen-6) (S5E Fig).

*Revised version (Fourth paragraph in Differentiation Atlas Section):*

Besides, we found a cell state that highly expressed *Ly6a* (stem cell antigen-6), known markers of progenitor-like cells^[109]^ ^[110]^.

###### 6.e

eA significant overlap between LepR+ and Cxcl12+ BMSCs has been well-recognized in the field.

Thank you for your very kind and helpful reminders. We have made corresponding revisions.

*Previous version (Fifth paragraph in Differentiation Atlas Section):*

Interestingly, *Cxcl12*+ BMSC, *Grem1*+ BMSC, and *Cdh2*+ BMSC identified in previous studies^5 6 7^ all demonstrated significant overlap with LepR+ BMSC in our atlas (Fig 2B).

*Revised version (Fifth paragraph in Differentiation Atlas Section):*

Interestingly, *Grem1*+ BMSC, and *Cdh2*+ BMSC identified in previous studies^5 6 7^ demonstrated significant overlap with LepR+ BMSC in our atlas (Fig 2B).

###### 6.f

It should be noted that Hoxa11(+) cells only exist in the zeugopod (ulna, radius, tibia, and fibula).

Thank you for your kindness and detailed suggestions. *Hoxa11* is tissue-specific and expressed only in the embryonic limb according to our atlas (Fig 29 B), making it inappropriate to represent early mesenchyme differentiation. Therefore, we have replaced it with *Msx2*(+), which is expressed in both the limb and head (Fig 29C). Additionally, *Msx2*(+) has been reported to be related to osteogenesis in both limb bud^[111]^ and head^[112]^.

We have revised Fig 3E (in manuscript) accordingly (Fig 29 A).

Fig 29 **A**, Revised version of Fig 3E (in manuscript). **B**, *Hox11a* only expressed in limb buds. **C**, Msx2 both expressed in heads and limb buds.

###### 6.g

Is TRAV a particular class of a metagene? If so, it should be clearly stated. If not, the authors need to explain further how it relates to a metagene.

Thank you for your detailed critique and valuable insights.

Yes, TRAV is a particular class of a metagene specially designed for large-scale datasets. Metagene^[113]^ and TRAV both aim to de novo cluster genes based on similar expression patterns. However, there are two main differences: 1. Metagenes are typically detected from a single dataset, whereas TRAVs are derived from multiple samples. 2. TRAV focus more on differentiation. In TRAVMap, we utilize differentiation as a key axis that spans multiple samples. By "projecting" gene expression data from various datasets onto this axis, we aimed to identify gene programs that consistently emerge. This method enabled us to uncover pseudotemporal gene modules that recur across different samples. Given the similarity to the "Replicable Axes of Variation" (RAV) approach described by Sehyun et al., we referred to our method as Trajectory-related Replicable Axes of Variation (TRAV)^[114]^.

To highlight the novelty of TRAVMap, we have revised our manuscript to emphasize the limitations of current metagene detection methods. (Note: We use the term "gene module" to refer to metagene, as it is more frequently used in the scRNA-seq field.)

*Previous version (Paragraph 1, TRAVMAP Section) :*

During differentiation, gene modules execute functions in a specific temporal order^[115]^. However, existing gene module identification methods^115^ fail to consider this temporal information. To address this limitation, we developed **TRAVMap**, a method to identify conserved pseudotemporal gene modules across multiple differentiation trajectories (Fig 5A). **TRAVMap** utilizes matrix factorization to identify recurring axes of variation^[116]^ within these trajectories, termed Trajectory-related Replicable Axes of Variation (TRAVs) (Fig 5A). We found that genes that drive TRAVs show coordinated expression patterns, representing gene modules that function at specific stages of the differentiation process (S21D,F,H Fig). As far as we know, this is the first algorithm that identified pseudotemproal gene modules. Furthermore, we found that the identified gene modules are conserved across multiple samples, validating the effectiveness and robustness of our algorithm (S21H Fig).

*Revised version (Paragraph 1, TRAVMAP Section):*

During differentiation, groups of genes participate collectively to execute specific functions are referred to as gene modules^[117]^. However, current gene module detection methods can’t integrate multiple datasets to yield more reliable results. To address this limitation, we developed **TRAVMap**, a method for identifying conserved pseudotemporal gene modules across multiple differentiation trajectories (Fig 5A). **TRAVMap** utilizes pseudotime as an axis, and by projecting gene expression onto this axis, it identifies recurring axes of variation^[118]^ within these trajectories, termed Trajectory-related Replicable Axes of Variation (TRAVs) (Fig 5A). We found that genes that drive TRAVs show coordinated expression patterns, representing gene modules that function at specific stages of the differentiation process (S21D,F,H Fig). Additionally, these gene modules are conserved across multiple samples, demonstrating the robustness and effectiveness of our algorithm (S21H Fig). **TRAVMap** distinguishes itself from existing gene module detection algorithms in two key ways: (1) it integrates multiple samples to derive more robust gene modules, and (2) it focuses specifically on pseudotemporal dynamics, offering insights into gene function across the temporal progression of differentiation.

## Reference

1. Mizoguchi T, Ono N. The diverse origin of bone-forming osteoblasts. Journal of Bone and Mineral Research. 2021;36(8):1432–47.
2. Sivaraj KK, Jeong HW, Dharmalingam B, Zeuschner D, Adams S, Potente M, et al. Regional specialization and fate specification of bone stromal cells in skeletal development. Cell Reports [Internet]. 2021 Jul 7 [cited 2022 Oct 30];36(2). Available from: https://www.ncbi.nlm.nih.gov/pmc/articles/PMC8293626/
3. Baryawno N, Przybylski D, Kowalczyk MS, Kfoury Y, Severe N, Gustafsson K, et al. A Cellular Taxonomy of the Bone Marrow Stroma in Homeostasis and Leukemia. Cell. 2019 Jun 13;177(7):1915-1932.e16.
4. Zhong L, Yao L, Tower RJ, Wei Y, Miao Z, Park J, et al. Single cell transcriptomics identifies a unique adipose lineage cell population that regulates bone marrow environment. Crump JG, Rosen CJ, Crump JG, editors. eLife. 2020 Apr 14;9:e54695.
5. Street K, Risso D, Fletcher RB, Das D, Ngai J, Yosef N, et al. Slingshot: cell lineage and pseudotime inference for single-cell transcriptomics. BMC Genomics. 2018 Dec;19(1):1–16.
6. Van den Berge K, Roux de Bézieux H, Street K, Saelens W, Cannoodt R, Saeys Y, et al. Trajectory-based differential expression analysis for single-cell sequencing data. Nat Commun. 2020 Mar 5;11(1):1201.
7. Trapnell C. Defining cell types and states with single-cell genomics. Genome Res. 2015 Oct 1;25(10):1491–8.
8. Sikkema L, Ramírez-Suástegui C, Strobl DC, Gillett TE, Zappia L, Madissoon E, et al. An integrated cell atlas of the lung in health and disease. Nat Med. 2023 Jun;29(6):1563–77.
9. Steuernagel L, Lam BYH, Klemm P, Dowsett GKC, Bauder CA, Tadross JA, et al. HypoMap—a unified single-cell gene expression atlas of the murine hypothalamus. Nat Metab. 2022 Oct;4(10):1402–19.
10. Aibar S, González-Blas CB, Moerman T, Huynh-Thu VA, Imrichova H, Hulselmans G, et al. SCENIC: Single-cell regulatory network inference and clustering. Nat Methods. 2017 Nov;14(11):1083–6.
11. Hao Y, Stuart T, Kowalski MH, Choudhary S, Hoffman P, Hartman A, et al. Dictionary learning for integrative, multimodal and scalable single-cell analysis. Nat Biotechnol. 2024 Feb;42(2):293–304.
12. Hou W, Ji Z, Chen Z, Wherry EJ, Hicks SC, Ji H. A statistical framework for differential pseudotime analysis with multiple single-cell RNA-seq samples. Nat Commun. 2023 Nov 10;14(1):7286.
13. Van den Berge K, Roux de Bézieux H, Street K, Saelens W, Cannoodt R, Saeys Y, et al. Trajectory-based differential expression analysis for single-cell sequencing data. Nat Commun. 2020 Mar 5;11(1):1201.
14. Wolf FA, Hamey FK, Plass M, et al. PAGA: graph abstraction reconciles clustering with trajectory inference through a topology preserving map of single cells. Genome Biol. 2019;20(1):1-9. doi:10.1186/s13059-019-1663-x
15. Trapnell C. Defining cell types and states with single-cell genomics. Genome Res. 2015 Oct 1;25(10):1491–8.
16. Street K, Risso D, Fletcher RB, Das D, Ngai J, Yosef N, et al. Slingshot: cell lineage and pseudotime inference for single-cell transcriptomics. BMC Genomics. 2018 Dec;19(1):1–16.
17. Van den Berge K, Roux de Bézieux H, Street K, Saelens W, Cannoodt R, Saeys Y, et al. Trajectory-based differential expression analysis for single-cell sequencing data. Nat Commun. 2020 Mar 5;11(1):1201.
18. Keidar Haran T, Keren L. From genes to modules, from cells to ecosystems. Molecular Systems Biology. 2022 May;18(5):e10726.
19. Kotliar D, Veres A, Nagy MA, Tabrizi S, Hodis E, Melton DA, et al. Identifying gene expression programs of cell-type identity and cellular activity with single-cell RNA-Seq. Valencia A, Barkai N, Mereu E, Göttgens B, editors. eLife. 2019 Jul 8;8:e43803.
20. Morabito S, Reese F, Rahimzadeh N, Miyoshi E, Swarup V. hdWGCNA identifies co-expression networks in high-dimensional transcriptomics data. 2023 Jun 26 [cited 2024 Aug 10]; Available from: https://www.ncbi.nlm.nih.gov/pmc/articles/PMC10326379/
21. Zhou P, Wang S, Li T, Nie Q. Dissecting transition cells from single-cell transcriptome data through multiscale stochastic dynamics. Nature Communications. 2021 Sep 23;12(1):5609.
22. Mizoguchi T, Ono N. The diverse origin of bone-forming osteoblasts. Journal of Bone and Mineral Research. 2021;36(8):1432–47.
23. Chari T, Pachter L. The specious art of single-cell genomics. [cited 2024 Aug 14]; Available from: https://journals.plos.org/ploscompbiol/article?id=10.1371/journal.pcbi.1011288
24. Amid E, Warmuth MK. arXiv.org. [cited 2024 Aug 8]. TriMap: Large-scale Dimensionality Reduction Using Triplets. Available from: https://arxiv.org/abs/1910.00204v2
25. Zeng H. What is a cell type and how to define it? Cell. 2022 Jul 21;185(15):2739–55.
26. Steuernagel L, Lam BYH, Klemm P, Dowsett GKC, Bauder CA, Tadross JA, et al. HypoMap—a unified single-cell gene expression atlas of the murine hypothalamus. Nat Metab. 2022 Oct;4(10):1402–19.
27. Yao Z, van Velthoven CTJ, Kunst M, Zhang M, McMillen D, Lee C, et al. A high-resolution transcriptomic and spatial atlas of cell types in the whole mouse brain. Nature. 2023 Dec 13;624(7991):317–32.
28. Zeng H. What is a cell type and how to define it? Cell. 2022 Jul 21;185(15):2739–55.
29. Domcke S, Shendure J. A reference cell tree will serve science better than a reference cell atlas. Cell. 2023 Mar 16;186(6):1103–14.
30. Baryawno N, Przybylski D, Kowalczyk MS, Kfoury Y, Severe N, Gustafsson K, et al. A Cellular Taxonomy of the Bone Marrow Stroma in Homeostasis and Leukemia. Cell. 2019 Jun 13;177(7):1915-1932.e16.
31. Wolock SL, Krishnan I, Tenen DE, Matkins V, Camacho V, Patel S, et al. Mapping Distinct Bone Marrow Niche Populations and Their Differentiation Paths. Cell Reports. 2019 Jul 9;28(2):302-311.e5.
32. Sivaraj KK, Jeong HW, Dharmalingam B, Zeuschner D, Adams S, Potente M, et al. Regional specialization and fate specification of bone stromal cells in skeletal development. Cell Reports [Internet]. 2021 Jul 7 [cited 2022 Oct 30];36(2). Available from: https://www.ncbi.nlm.nih.gov/pmc/articles/PMC8293626/
33. Shu HS, Liu YL, Tang XT, Zhang XS, Zhou B, Zou W, et al. Tracing the skeletal progenitor transition during postnatal bone formation. Cell Stem Cell. 2021 Dec 2;28(12):2122-2136.e3.
34. Sikkema L, Ramírez-Suástegui C, Strobl DC, Gillett TE, Zappia L, Madissoon E, et al. An integrated cell atlas of the lung in health and disease. Nat Med. 2023 Jun;29(6):1563–77.
35. Xu Q, Halle L, Hediyeh-zadeh S, Kuijs M, Kilik U, Yu Q, et al. An integrated transcriptomic cell atlas of human endoderm-derived organoids [Internet]. bioRxiv; 2023 [cited 2023 Nov 21]. p. 2023.11.20.567825. Available from: https://www.biorxiv.org/content/10.1101/2023.11.20.567825v1
36. Wolf FA, Hamey FK, Plass M, Solana J, Dahlin JS, Göttgens B, et al. PAGA: graph abstraction reconciles clustering with trajectory inference through a topology preserving map of single cells. Genome Biology. 2019 Mar 19;20(1):59.
37. Wolf FA, Angerer P, Theis FJ. SCANPY: large-scale single-cell gene expression data analysis. Genome Biol. 2018 Feb 6;19:15.
38. Lange M, Bergen V, Klein M, Setty M, Reuter B, Bakhti M, et al. CellRank for directed single-cell fate mapping. Nat Methods. 2022 Feb;19(2):159–70.
39. Bergen V, Lange M, Peidli S, Wolf FA, Theis FJ. Generalizing RNA velocity to transient cell states through dynamical modeling. Nat Biotechnol. 2020 Dec;38(12):1408–14.
40. Wolf FA, Hamey FK, Plass M, Solana J, Dahlin JS, Göttgens B, et al. PAGA: graph abstraction reconciles clustering with trajectory inference through a topology preserving map of single cells. Genome Biology. 2019 Mar 19;20(1):59.
41. Alemany A, Florescu M, Baron CS, Peterson-Maduro J, van Oudenaarden A. Whole-organism clone tracing using single-cell sequencing. Nature. 2018 Apr;556(7699):108–12.
42. Wagner DE, Weinreb C, Collins ZM, Briggs JA, Megason SG, Klein AM. Single-cell mapping of gene expression landscapes and lineage in the zebrafish embryo. Science. 2018 Jun 1;360(6392):981–7.
43. Nowotschin S, Setty M, Kuo YY, Liu V, Garg V, Sharma R, et al. The emergent landscape of the mouse gut endoderm at single-cell resolution. Nature. 2019 May;569(7756):361–7.
44. Hou W, Ji Z, Chen Z, Wherry EJ, Hicks SC, Ji H. A statistical framework for differential pseudotime analysis with multiple single-cell RNA-seq samples. Nat Commun. 2023 Nov 10;14(1):7286.
45. De Donno C, Hediyeh-Zadeh S, Moinfar AA, Wagenstetter M, Zappia L, Lotfollahi M, et al. Population-level integration of single-cell datasets enables multi-scale analysis across samples. Nat Methods. 2023 Oct 9;1–10.
46. De Donno C, Hediyeh-Zadeh S, Moinfar AA, Wagenstetter M, Zappia L, Lotfollahi M, et al. Population-level integration of single-cell datasets enables multi-scale analysis across samples. Nat Methods. 2023 Oct 9;1–10.
47. De Donno C, Hediyeh-Zadeh S, Moinfar AA, Wagenstetter M, Zappia L, Lotfollahi M, et al. Population-level integration of single-cell datasets enables multi-scale analysis across samples. Nat Methods. 2023 Oct 9;1–10.
48. Sikkema L, Ramírez-Suástegui C, Strobl DC, Gillett TE, Zappia L, Madissoon E, et al. An integrated cell atlas of the lung in health and disease. Nat Med. 2023 Jun;29(6):1563–77.
49. Li H, McCarthy DJ, Shim H, Wei S. Trade-off between conservation of biological variation and batch effect removal in deep generative modeling for single-cell transcriptomics. BMC Bioinformatics. 2022 Nov 3;23(1):1–22.
50. Steuernagel L, Lam BYH, Klemm P, Dowsett GKC, Bauder CA, Tadross JA, et al. HypoMap—a unified single-cell gene expression atlas of the murine hypothalamus. Nat Metab. 2022 Oct;4(10):1402–19.
51. Sikkema L, Ramírez-Suástegui C, Strobl DC, Gillett TE, Zappia L, Madissoon E, et al. An integrated cell atlas of the lung in health and disease. Nat Med. 2023 Jun;29(6):1563–77.
52. Büttner M, Miao Z, Wolf FA, Teichmann SA, Theis FJ. A test metric for assessing single-cell RNA-seq batch correction. Nat Methods. 2019 Jan;16(1):43–9.
53. Sivaraj KK, Jeong HW, Dharmalingam B, Zeuschner D, Adams S, Potente M, et al. Regional specialization and fate specification of bone stromal cells in skeletal development. Cell Reports [Internet]. 2021 Jul 7 [cited 2022 Oct 30];36(2). Available from: https://www.ncbi.nlm.nih.gov/pmc/articles/PMC8293626/
54. Angelozzi M, Pellegrino da Silva R, Gonzalez MV, Lefebvre V. Single-cell atlas of craniogenesis uncovers SOXC-dependent, highly proliferative, and myofibroblast-like osteodermal progenitors. Cell Rep. 2022 Jul 12;40(2):111045.
55. Luecken MD, Büttner M, Chaichoompu K, Danese A, Interlandi M, Mueller MF, et al. Benchmarking atlas-level data integration in single-cell genomics. Nat Methods. 2022 Jan;19(1):41–50.
56. Muruganandan S, Pierce R, Teguh DA, Perez RF, Bell N, Nguyen B, et al. A FoxA2+ long-term stem cell population is necessary for growth plate cartilage regeneration after injury. Nat Commun. 2022 May 6;13(1):2515.
57. Shi Y, He G, Lee WC, McKenzie JA, Silva MJ, Long F. Gli1 identifies osteogenic progenitors for bone formation and fracture repair. Nat Commun. 2017 Dec 11;8(1):2043.
58. 1. Skinnider MA, Squair JW, Kathe C, Anderson MA, Gautier M, Matson KJE, et al. Cell type prioritization in single-cell data. Nat Biotechnol. 2021 Jan 1;39(1):30–4.
59. 1. Yang G, He Q, Guo X, Li RY, Lin J, Lang Y, et al. Identification of the metaphyseal skeletal stem cell building trabecular bone. Sci Adv. 10(8):eadl2238.
60. Zhu S, Chen W, Masson A, Li YP. Cell signaling and transcriptional regulation of osteoblast lineage commitment, differentiation, bone formation, and homeostasis. Cell Discovery. 2024 Jul 2;10(1):1–39.
61. Collins JM, Lang A, Parisi C, Moharrer Y, Nijsure MP, (Thomas) Kim JH, et al. YAP and TAZ couple osteoblast precursor mobilization to angiogenesis and mechanoregulation in murine bone development. Developmental Cell 2024 Feb 2; 59(2): 211-227. e5.
62. Bressan D, Battistoni G, Hannon GJ. The dawn of spatial omics. Science. 2023 Aug 4;381(6657):eabq4964.
63. Mizoguchi T, Ono N. The diverse origin of bone-forming osteoblasts. Journal of Bone and Mineral Research. 2021;36(8):1432–47.
64. Wagner DE, Klein AM. Lineage tracing meets single-cell omics: opportunities and challenges. Nature Reviews Genetics. 2020 Jul;21(7):410–27.
65. Zhu S, Chen W, Masson A, Li YP. Cell signaling and transcriptional regulation of osteoblast lineage commitment, differentiation, bone formation, and homeostasis. Cell Discovery. 2024 Jul 2;10(1):1–39.
66. Collins JM, Lang A, Parisi C, Moharrer Y, Nijsure MP, (Thomas) Kim JH, et al. YAP and TAZ couple osteoblast precursor mobilization to angiogenesis and mechanoregulation in murine bone development. Developmental Cell 2024 Feb 2; 59(2): 211-227. e5.
67. Bressan D, Battistoni G, Hannon GJ. The dawn of spatial omics. Science. 2023 Aug 4;381(6657):eabq4964.
68. Baryawno N, Przybylski D, Kowalczyk MS, Kfoury Y, Severe N, Gustafsson K, et al. A Cellular Taxonomy of the Bone Marrow Stroma in Homeostasis and Leukemia. Cell. 2019 Jun 13;177(7):1915-1932.e16.
69. Mizuhashi K, Nagata M, Matsushita Y, Ono W, Ono N. Growth Plate Borderline Chondrocytes Behave as Transient Mesenchymal Precursor Cells. Journal of Bone and Mineral Research. 2019;34(8):1387–92.
70. Wagner DE, Klein AM. Lineage tracing meets single-cell omics: opportunities and challenges. Nature Reviews Genetics. 2020 Jul;21(7):410–27.
71. Worthley DL, Churchill M, Compton JT, Tailor Y, Rao M, Si Y, et al. Gremlin 1 Identifies a Skeletal Stem Cell with Bone, Cartilage, and Reticular Stromal Potential. Cell. 2015 Jan 15;160(1):269–84.
72. Ambrosi TH, Sinha R, Steininger HM, Hoover MY, Murphy MP, Koepke LS, et al. Distinct skeletal stem cell types orchestrate long bone skeletogenesis. Ackert-Bicknell C, Cheah KSE, Sherk V, editors. eLife. 2021 Jul 19;10:e66063.
73. Hao RC, Li ZL, Wang FY, Tang J, Li PL, Yin BF, et al. Single-cell transcriptomic analysis identifies a highly replicating Cd168+ skeletal stem/progenitor cell population in mouse long bones. Journal of Genetics and Genomics. 2023 Sep 1;50(9):702–12.
74. Nishikawa K, Nakashima T, Takeda S, Isogai M, Hamada M, Kimura A, et al. Maf promotes osteoblast differentiation in mice by mediating the age-related switch in mesenchymal cell differentiation. J Clin Invest. 2010 Oct 1;120(10):3455–65.
75. Infante A, Rodríguez CI. Osteogenesis and aging: lessons from mesenchymal stem cells. Stem Cell Res Ther. 2018 Dec;9(1):1–7.
76. Kimmel JC, Penland L, Rubinstein ND, Hendrickson DG, Kelley DR, Rosenthal AZ. Murine single-cell RNA-seq reveals cell-identity- and tissue-specific trajectories of aging. Genome Res. 2019 Dec;29(12):2088–103.
77. Vattulainen M, Smits JGA, Cunha DL, Ilmarinen T, Skottman H, Zhou H. Deciphering the heterogeneity of differentiating hPSC-derived corneal limbal stem cells through single-cell RNA-sequencing. Published online November 24, 2023:2023.11.24.568553. doi:10.1101/2023.11.24.568553
78. Nishikawa K, Nakashima T, Takeda S, Isogai M, Hamada M, Kimura A, et al. Maf promotes osteoblast differentiation in mice by mediating the age-related switch in mesenchymal cell differentiation. J Clin Invest. 2010 Oct 1;120(10):3455–65.
79. Infante A, Rodríguez CI. Osteogenesis and aging: lessons from mesenchymal stem cells. Stem Cell Res Ther. 2018 Dec;9(1):1–7.
80. Hendrickx G, Boudin E, Van Hul W. A look behind the scenes: the risk and pathogenesis of primary osteoporosis. Nat Rev Rheumatol. 2015 Aug;11(8):462–74.
81. Stapleton M, Sawamoto K, Alméciga-Díaz CJ, Mackenzie WG, Mason RW, Orii T, et al. Development of Bone Targeting Drugs. International Journal of Molecular Sciences. 2017 Jul;18(7):1345.
82. Zhang X, Jiang W, Xie C, Wu X, Ren Q, Wang F, et al. Msx1+ stem cells recruited by bioactive tissue engineering graft for bone regeneration. Nat Commun. 2022 Sep 5;13:5211.
83. Nishikawa K, Nakashima T, Takeda S, Isogai M, Hamada M, Kimura A, et al. Maf promotes osteoblast differentiation in mice by mediating the age-related switch in mesenchymal cell differentiation. J Clin Invest. 2010 Oct 1;120(10):3455–65.
84. Infante A, Rodríguez CI. Osteogenesis and aging: lessons from mesenchymal stem cells. Stem Cell Res Ther. 2018 Dec;9(1):1–7.
85. Nishikawa K, Nakashima T, Takeda S, Isogai M, Hamada M, Kimura A, et al. Maf promotes osteoblast differentiation in mice by mediating the age-related switch in mesenchymal cell differentiation. J Clin Invest. 2010 Oct 1;120(10):3455–65.
86. Van den Berge K, Roux de Bézieux H, Street K, Saelens W, Cannoodt R, Saeys Y, et al. Trajectory-based differential expression analysis for single-cell sequencing data. Nat Commun. 2020 Mar 5;11(1):1201.
87. Zhu S, Chen W, Masson A, Li YP. Cell signaling and transcriptional regulation of osteoblast lineage commitment, differentiation, bone formation, and homeostasis. Cell Discovery. 2024 Jul 2;10(1):1–39.
88. Hou W, Ji Z, Chen Z, Wherry EJ, Hicks SC, Ji H. A statistical framework for differential pseudotime analysis with multiple single-cell RNA-seq samples. Nat Commun. 2023 Nov 10;14(1):7286.
89. Bok S, Yallowitz AR, Sun J, McCormick J, Cung M, Hu L, et al. A multi-stem cell basis for craniosynostosis and calvarial mineralization. Nature. 2023 Sep;621(7980):804–12.
90. Debnath S, Yallowitz AR, McCormick J, Lalani S, Zhang T, Xu R, et al. Discovery of a periosteal stem cell mediating intramembranous bone formation. Nature. 2018 Oct;562(7725):133–9.
91. Galea GL, Zein MR, Allen S, Francis‐West P. Making and shaping endochondral and intramembranous bones. Dev Dyn. 2021 Mar;250(3):414–49.
92. Galea GL, Zein MR, Allen S, Francis‐West P. Making and shaping endochondral and intramembranous bones. Dev Dyn. 2021 Mar;250(3):414–49.
93. Debnath S, Yallowitz AR, McCormick J, Lalani S, Zhang T, Xu R, et al. Discovery of a periosteal stem cell mediating intramembranous bone formation. Nature. 2018 Oct;562(7725):133–9.
94. Mo C, Guo J, Qin J, Zhang X, Sun Y, Wei H, et al. Single‐cell transcriptomics of LepR‐positive skeletal cells reveals heterogeneous stress‐dependent stem and progenitor pools. EMBO J. 2022 Feb 15;41(4):e108415.
95. Duchamp de Lageneste O, Julien A, Abou-Khalil R, Frangi G, Carvalho C, Cagnard N, et al. Periosteum contains skeletal stem cells with high bone regenerative potential controlled by Periostin. Nat Commun. 2018 Feb 22;9(1):773.
96. Mizuhashi K, Ono W, Matsushita Y, Sakagami N, Takahashi A, Saunders TL, et al. Resting zone of the growth plate houses a unique class of skeletal stem cells. Nature. 2018 Nov;563(7730):254–8.
97. Mizuhashi K, Ono W, Matsushita Y, Sakagami N, Takahashi A, Saunders TL, et al. Resting zone of the growth plate houses a unique class of skeletal stem cells. Nature. 2018 Nov;563(7730):254–8.
98. Ding DC, Shyu WC, Lin SZ. Mesenchymal Stem Cells. Cell Transplant. 2011 Feb 1;20(1):5–14.
99. Ambrosi TH, Longaker MT, Chan CKF. A Revised Perspective of Skeletal Stem Cell Biology. Frontiers in Cell and Developmental Biology [Internet]. 2019 [cited 2022 May 29];7. Available from: https://www.frontiersin.org/article/10.3389/fcell.2019.00189
100. Angelozzi M, Pellegrino da Silva R, Gonzalez MV, Lefebvre V. Single-cell atlas of craniogenesis uncovers SOXC-dependent, highly proliferative, and myofibroblast-like osteodermal progenitors. Cell Rep. 2022 Jul 12;40(2):111045.
101. He P, Williams BA, Trout D, Marinov GK, Amrhein H, Berghella L, et al. The changing mouse embryo transcriptome at whole tissue and single-cell resolution. Nature. 2020 Jul;583(7818):760–7.
102. He J, Yan J, Wang J, Zhao L, Xin Q, Zeng Y, et al. Dissecting human embryonic skeletal stem cell ontogeny by single-cell transcriptomic and functional analyses. Cell Res. 2021 Jul;31(7):742–57.
103. Matsushita Y, Manabe H, Ohyama T, Nakamura S, Nagata M, Ono W, et al. Hes1 marks peri-condensation mesenchymal cells that generate both chondrocytes and perichondrial cells in early bone development. J Biol Chem. 2023 May 11;299(6):104805.
104. Yuan Y, Loh Y hwee E, Han X, Feng J, Ho TV, He J, et al. Spatiotemporal cellular movement and fate decisions during first pharyngeal arch morphogenesis. Sci Adv. 2020 Dec 16;6(51):eabb0119.
105. Soldatov R, Kaucka M, Kastriti ME, Petersen J, Chontorotzea T, Englmaier L, et al. Spatiotemporal structure of cell fate decisions in murine neural crest. Science. 2019 Jun 7;364(6444):eaas9536.
106. Sakagami N, Matsushita Y, Syklawer-Howle S, Kronenberg HM, Ono W, Ono N. Msx2 Marks Spatially Restricted Populations of Mesenchymal Precursors. J Dent Res. 2018 Oct 1;97(11):1260–7.
107. Buechler MB, Pradhan RN, Krishnamurty AT, Cox C, Calviello AK, Wang AW, et al. Cross-tissue organization of the fibroblast lineage. Nature. 2021 May;593(7860):575–9.
108. Baryawno N, Przybylski D, Kowalczyk MS, Kfoury Y, Severe N, Gustafsson K, et al. A Cellular Taxonomy of the Bone Marrow Stroma in Homeostasis and Leukemia. Cell. 2019 Jun 13;177(7):1915-1932.e16.
109. Buechler MB, Pradhan RN, Krishnamurty AT, Cox C, Calviello AK, Wang AW, et al. Cross-tissue organization of the fibroblast lineage. Nature. 2021 May;593(7860):575–9.
110. Baryawno N, Przybylski D, Kowalczyk MS, Kfoury Y, Severe N, Gustafsson K, et al. A Cellular Taxonomy of the Bone Marrow Stroma in Homeostasis and Leukemia. Cell. 2019 Jun 13;177(7):1915-1932.e16.
111. Bensoussan-Trigano V, Lallemand Y, Saint Cloment C, Robert B. Msx1 and Msx2 in limb mesenchyme modulate digit number and identity. Developmental Dynamics. 2011;240(5):1190–202.
112. Sakagami N, Matsushita Y, Syklawer-Howle S, Kronenberg HM, Ono W, Ono N. Msx2 Marks Spatially Restricted Populations of Mesenchymal Precursors. J Dent Res. 2018 Oct 1;97(11):1260–7.
113. Lombardi O, Li R, Halim S, Choudhry H, Ratcliffe PJ, Mole DR. Pan-cancer analysis of tissue and single-cell HIF-pathway activation using a conserved gene signature. [cited 2024 Aug 18]; Available from: https://www.cell.com/cell-reports/abstract/S2211-1247(22)01523-6
114. Oh S, Geistlinger L, Ramos M, Blankenberg D, van den Beek M, Taroni JN, et al. GenomicSuperSignature facilitates interpretation of RNA-seq experiments through robust, efficient comparison to public databases. Nat Commun. 2022 Jun 27;13(1):3695.
115. Saelens W, Cannoodt R, Saeys Y. A comprehensive evaluation of module detection methods for gene expression data. Nat Commun. 2018 Mar 15;9(1):1090.
116. Oh S, Geistlinger L, Ramos M, Blankenberg D, van den Beek M, Taroni JN, et al. GenomicSuperSignature facilitates interpretation of RNA-seq experiments through robust, efficient comparison to public databases. Nat Commun. 2022 Jun 27;13(1):3695.
117. Saelens W, Cannoodt R, Saeys Y. A comprehensive evaluation of module detection methods for gene expression data. Nat Commun. 2018 Mar 15;9(1):1090.
118. Oh S, Geistlinger L, Ramos M, Blankenberg D, van den Beek M, Taroni JN, et al. GenomicSuperSignature facilitates interpretation of RNA-seq experiments through robust, efficient comparison to public databases. Nat Commun. 2022 Jun 27;13(1):3695.
