# Supplementary material for: Trajectory-centric framework TrajAtlas reveals multi-scale differentiation heterogeneity among cells, genes, and gene modules in osteogenesis
Source: PLoS Genet. 2024 Oct 22;20(10):e1011319. doi: 10.1371/journal.pgen.1011319 (PMC11530032; doi:10.1371/journal.pgen.1011319)
Supplement: Attachment — Submitted filename: 9.26_Responses_to _the_Reviewers_Second_Round.docx [file pgen.1011319.s036.docx]

Dear Editors and Reviewers:

Thank you for your letter and the detailed comments regarding our manuscript (PGENETICS-D-24-00584) titled “Trajectory-centric framework TrajAtlas reveals multi-scale differentiation heterogeneity among cells, genes, and gene modules in osteogenesis.” We greatly appreciate the insightful comments, which have been invaluable for revising and enhancing our paper, as well as providing important guidance for our research team. We have carefully considered the reviewers’ suggestions and have made corresponding modifications to the manuscript.

The point-by-point responses addressing the reviewers’ comments are listed below. Our responses and corresponding changes are highlighted in the text in both this letter and the revised manuscript.

We are looking forward to receiving your favorable reply.

Yours sincerely,

Yufeng Zhang, Ph.D., DDS

School & Hospital of Stomatology, Wuhan University

237 Luoyu Road, Hongshan District, Wuhan 430079, China

E-mail: zyf@whu.edu.cn

Huan Liu, Ph.D., DDS

School & Hospital of Stomatology, Wuhan University

237 Luoyu Road, Wuhan 430079, China

E-mail: liu.huan@whu.edu.cn

**Responses to the Reviewers’ Comments and the Corresponding Revisions**

**Summary** The resubmitted version of Han et al. is a significantly improved version, with a much clearer abstract, introduction and more explanatory details throughout the main text. Whereas my major concern with the first submission was its unclear presentation, the current version has much improved readability. The authors have also addressed all my technical questions with a lot of additional computational experiments, providing more justification to their modelling strategy.

I have some minor comments regarding the presentation of results.

Thank you for your valuable feedback. It has been incredibly helpful in improving the quality of our manuscript.

1. As I mentioned in the previous review, a merit of this paper is its carefully benchmarked computational workflow towards building the atlas. What I did not notice was that this work also included a shiny app and well-documented tutorial. These additional resources were highlighted in the authors’ response to reviewers but not sufficiently highlighted in the paper. As a paper on building atlas, the computational methods are also of significant value to the reader. I think it’s worth mentioning the computational methodology and additional resources in the introduction. In the current version, the last paragraph in Introduction is on the methodology but it’s rather vague.

Thank you for your positive feedback and critiques on the introduction. We have revised the last paragraph to enhance readability. Additionally, we’ve added the website of the [Shiny app](https://zyflab.shinyapps.io/TrajAtlas_shiny/) and the [tutorial](https://trajatlas.readthedocs.io/en/stable/index.html) at the end of the introduction. During the revision process, we have updated our Shiny app to make it more user-friendly (Figure 1) and incorporated extra features to the software based on user feedback ([feature1](https://trajatlas.readthedocs.io/en/stable/tutorial/spaital_omic.html), [feature2](https://trajatlas.readthedocs.io/en/stable/tutorial/gssaxis.html)). We hope these modifications will lead to broader usage of our framework.

*Previous version (last paragraph of Introduction):*

To address the complexity and heterogeneity inherent in osteoblast differentiation, our study introduces TrajAtlas, a trajectory-centric framework specifically designed to navigate and elucidate these challenges. TrajAtlas encompasses a reference atlas for osteoblast differentiation coupled with a sophisticated seven-layer hierarchical annotation system, allowing for the detailed modeling of differentiation pathways from diverse osteoprogenitor cells to mature osteoblasts. It features advanced tools for dissecting local variations along trajectories, facilitating the identification of differentially expressed genes during differentiation, and uncovering trajectory-related gene modules to infer their activity within these paths. By leveraging TrajAtlas for an in-depth analysis of cellular, genetic, and gene module heterogeneity in large-scale trajectories, particularly focusing on the influences of age and injury on bone formation, our framework unveils novel insights into osteoblast differentiation. Importantly, while our framework is exemplified through the study of osteogenesis, it is designed to be broadly applicable. When supplemented with single-cell transcriptome datasets from other tissues or lineages, TrajAtlas can be adapted to reveal similar insights into the differentiation processes of various cell types, making it a versatile tool for understanding cellular differentiation across different biological contexts.

*Revised version (last paragraph of Introduction):*

To address the complexity and heterogeneity inherent in osteoblast differentiation, our study introduces **TrajAtlas**, a trajectory-centric framework specifically designed to navigate and elucidate these challenges. TrajAtlas encompasses a reference atlas for osteoblast differentiation coupled with a sophisticated seven-layer hierarchical annotation system that allows for the exploration of osteoprogenitor cell heterogeneity. In-depth, a model was introduced to reveal genetic regulation and differentiation pathways from diverse osteoprogenitor cells to mature osteoblasts. It features advanced tools for dissecting cell and gene dynamics along trajectories, facilitating the identification of age and tissue-specific variations and uncovering differentiation-related gene modules to infer their activity within these paths. By leveraging TrajAtlas, with a particular focus on the influences of age and injury on bone formation, our framework unveils novel insights into osteoblast differentiation. Importantly, while our framework is exemplified through the study of osteogenesis, it is designed to be broadly applicable. When supplemented with single-cell transcriptome datasets from other tissues or lineages, TrajAtlas can be adapted to reveal similar insights into the differentiation processes of various cell types, making it a versatile tool for understanding cellular differentiation across different biological contexts. We have made the associated datasets available for interactive exploration at [https://zyflab.shinyapps.io/TrajAtlas_shiny,](https://zyflab.shinyapps.io/TrajAtlas_shiny/,) and the corresponding computational framework as open-source software, available at <https://trajatlas.readthedocs.io.>

Figure 1

2. 215–219 on p. 9: the two sentences here are repetitive.

Thank you for your astute observation and for bringing this matter to our attention.

*Previous version :*

We then incorporated these experimentally validated osteoprogenitors based on the previously described marker genes and tissue locations (Methods). To assess the osteogenic potential of cells within our atlas, we incorporated experimentally validated osteoprogenitors based on previously described marker genes and tissue locations (Fig 2A).

*Revised version :*

To assess the osteogenic potential of cells within our atlas, we incorporated experimentally validated osteoprogenitors based on previously described marker genes and tissue locations (Fig 2A).

3. Overview of TrajAtlas provided a much clearer description of the four modules of TrajAtlas. I found the short descriptions in bold font easier to understand than the original module names, such as Differentiation Model. It’s worth repeating these short descriptions in subsequent sections to remind the reader what each module is about, e.g. at p/ 11 line 279.

Thank you for your attentive review and valuable feedback on improving the readability. We have followed your advice and repeat the description in the frist paragraph of every section.

*Revised version (in Differentiation Atlas, newly added):*

With the extensive curated atlas and detailed annotations, we were able to explore the heterogeneity of osteoprogenitor cells.

*Previous version (in Differentiation Model):*

To understand how various osteoprogenitor cells differentiate into osteoblasts differentially, we established a Differentiation Model within the Differentiation Atlas.

*Revised version (in Differentiation Model):*

To understand how various osteoprogenitor cells differentiate into osteoblasts differentially, we established a Differentiation Model that focuses on the genetic regulation and pathway dynamics during osteogenesis.

*Revised version (in TrajDiff, newly added):*

This algorithm enables us to detect age and tissue-specific variations in differentiation.

*Previous version (in TRAVMap):*

To address this limitation, we developed TRAVMap, a method for identifying conserved pseudotemporal gene modules across multiple differentiation trajectories.

*Revised version (in TRAVMap):*

To address this limitation, we developed TRAVMap, a method for identifying differentiation-related gene modules across multiple trajectories (Fig 5A).

4. Line 530 p. 20: I guess the transfer learning method here meant scANVI?

Thank you for your feedback. Yes, we mapped the curated extended atlas to the model trained with scANVI. While transfer learning refers to a broader concept in machine learning, our method, scArches, is a specific application based on transfer learning^[1]^. In the revised version, we provide a more precise statement to describe this process.

*Previous version :*

We then applied transfer learning to these datasets based on our Differentiation Atlas (Fig 6A).

*Revised version :*

Then, we mapped our extended datasets to the Differentiation Atlas using scArches, a reference mapping approach (Fig 6A).

5. Lines 547–562. This paragraph singled out a subset of cells from injury datasets to analyse since they had high uncertainty when projected to the atlas. However by examining Fig S23C, it seems that this group of cells did not have the highest uncertainty? Some explanations are needed for why choosing this particular subset of cells to analyse.

Thank you for your careful review and valuable feedback. We acknowledge the need for clearer explanations regarding our choice of this subset of cells. This subset was selected using a combination of data-driven insights and prior knowledge.

1) **Data-driven methods**: We employed Leiden clustering methods for our analysis, adjusting the resolution between 0.01 and 15 with 24 gradients. We then use uncertainty, measured during the label transfer process, to identify novel cell states^[2]^. An uncertainty threshold was established at the 80th percentile deviation from the core datasets. Clusters exhibiting higher mean uncertainty than this threshold were identified as candidate novel clusters. As the resolution increased to 0.5, two clusters emerged: cluster 6 (mesenchyme subcluster) and cluster 10 (chondrocyte subcluster) (Figure 1 A). Notably, cluster 6 (Injury Mes in Manuscript) displayed higher uncertainty scores (Figure 1 B). Besides, most cells of cluster 6 are derived from the extended atlas, suggesting that it is more likely to represent a new cell state not observed in our core datasets (Figure 1 C). The Python script for this algorithm is deposited at [github](https://github.com/GilbertHan1011/TrajAtlasManuscript/blob/main/0_utils/find_new_celltype.py).
2) **Prior Knowledge**: We found that this cluster is closely related to injury, with 88.1% of the cells derived from injury states (Figure 1 D). Interestingly, various tissues appear capable of generating these cell states, suggesting a unique mechanism for responding to injury. Moreover, this cell cluster may be closely associated with mesenchymal cells, which are known for their multipotential capabilities in embryonic development, as discussed in our manuscript. This observation indicates potential dedifferentiation or reprogramming, concepts widely explored in the field of regeneration. Therefore, we believe this cluster deserves further attention.

Figure 2

We have revised our manuscript and supplementary figure correspondingly.

*Revised version (Methods, newly added):*

We employed Leiden clustering methods for our analysis, adjusting the resolution between 0.01 and 15 with 24 gradients. We then use uncertainty, measured during label transfer process, to identify novel cell state [58]. An uncertainty threshold was established at the 80th percentile deviation from the core datasets. Clusters exhibiting higher mean uncertainty than this threshold were identified as candidate novel clusters.

*Previous version of S23 Fig.*

*Revised version of S23 Fig.*

1. Lotfollahi M, Naghipourfar M, Luecken MD, Khajavi M, Büttner M, Wagenstetter M, et al. Mapping single-cell data to reference atlases by transfer learning. Nat Biotechnol. 2022 Jan;40(1):121–30.
2. Sikkema L, Ramírez-Suástegui C, Strobl DC, Gillett TE, Zappia L, Madissoon E, et al. An integrated cell atlas of the lung in health and disease. Nat Med. 2023 Jun;29(6):1563–77.
